# Robots in Healthcare: a Scoping Review

**DOI:** 10.1007/s43154-022-00095-4

**Published:** 2022-10-22

**Authors:** Ahmed Ashraf Morgan, Jordan Abdi, Mohammed A. Q. Syed, Ghita El Kohen, Phillip Barlow, Marcela P. Vizcaychipi

**Affiliations:** 1 Chelsea and Westminster Hospital NHS Foundation Trust, London, UK; 2 University of Essex, Colchester, Essex UK; 3 Queen Mary University of London, London, UK; 4 Imperial College London, London, UK

**Keywords:** Robots, Robotics, Hospital, Healthcare, Surgery, Rehabilitation

## Abstract

**Purpose of Review:**

Robots are increasingly being adopted in healthcare to carry out various tasks that enhance patient care. This scoping review aims to establish the types of robots being used in healthcare and identify where they are deployed.

**Recent Findings:**

Technological advancements have enabled robots to conduct increasingly varied and complex roles in healthcare. For instance, precision tasks such as improving dexterity following stroke or assisting with percutaneous coronary intervention.

**Summary:**

This review found that robots have played 10 main roles across a variety of clinical environments. The two predominant roles were surgical and rehabilitation and mobility. Although robots were mainly studied in the surgical theatre and rehabilitation unit, other settings ranged from the hospital ward to inpatient pharmacy. Healthcare needs are constantly evolving, as demonstrated by COVID-19, and robots may assist in adapting to these changes. The future will involve increased telepresence and infrastructure systems will have to improve to allow for this.

**Supplementary Information:**

The online version contains supplementary material available at 10.1007/s43154-022-00095-4.

## Introduction

Since the advent of the COVID-19 pandemic, the healthcare industry has been flooded with novel technologies to assist the delivery of care in unprecedented circumstances. [1, 2] Staff vacancy levels increased, [3, 4] social restrictions curtailed many traditional means of care delivery, [5••] and stringent infection control measures brought new challenges to human-delivered care [6]. Although many of the challenges that the pandemic brought onto healthcare have subsided, staff burnout, [7] an increasingly elderly population, [8] and backlog strains [9, 10] caused by the pandemic have meant that staff shortages persist across healthcare systems across the world.

Robotic systems have long been cited to be able to alleviate workforce pressures, not least in healthcare. [11] Such systems can include remote presence robots for virtual consultations or transportation robots for automated delivery of equipment within hospitals. In addition to supporting hospitals, robotic systems can offer the ability to support clinical practice in a variety of specialties. Examples include exoskeletons that assist stroke patients in mobilisation and surgical robots that allow surgeons to remotely perform operations. It is important to understand the landscape of roles that robots have in healthcare to inform the research and development of the future.

This scoping review aims to establish the types of robots being used in healthcare and identify where they are deployed by way of qualitative analysis of the literature. Through this, predictions can be made for the future of robotics.

## Methodology

The protocol for this scoping review was conducted in accordance with the principles of the Cochrane Handbook for Systematic Reviews of Interventions [12].

### Search Strategy

The following bibliographical databases were searched: CINAHL, Cochrane Library, Embase, MEDLINE, and Scopus using medical subject headings (MeSH or where appropriate, the database-specific thesaurus equivalent) or text word terms. The database search query was composed of two search concepts: the intervention (robots) and the context (clinical setting). Free text terms for the intervention included: “service robot*”, “surgical robot*” and “socially assistive robot*”; their associated MeSH term was “Robotics”. The names of specific robot systems were also searched for. The free words used for the context included the following: “Inpatient setting”, “outpatient setting”, “pharmacy”, “trauma centre”, “acute centre”, "rehabilitation hospital”, “geriatric hospital” and “field hospital”; their associated MeSH term was “Hospitals”. The use of the asterisk (*) enables the word to be treated as a prefix. For example, “elder*” will represent “elderly” and “eldercare” amongst others (Supplementary Material A). Additional studies were selected through a free search (Google Scholar) and from reference lists of selected publications and relevant reviews. The search was conducted on 11th March 2022.

### Study Selection

Two reviewers (AM and MS) independently screened the publications in a three-step assessment process: the title, abstract and full text, and selections were made in accordance with inclusion and exclusion criteria. Inclusion: physical robot, used within a healthcare setting. Exclusion: review/meta-analysis, non-English, technical report, wrong setting, wrong intervention (e.g. artificial intelligence, no robot), full manuscript not available. All publications collected during the database search, free search and reference list harvesting were scored on a 3-point scale (0, not relevant; 1, possibly relevant; 2, very relevant) and those with a combined score of 2 between the reviews would make it through to the next round of scoring. All publications with a total score of 0 were excluded. A publication with a combined score of 1 indicated a disagreement between the reviewers and would be resolved through discussion. At the end of the full-text screening round, a final set of publications to be included into the review was acquired. Cohen’s kappa coefficient was calculated to ascertain the agreement between the reviewers in the title, abstract and full-text screening phases.

### Data Extraction

The data extraction form was designed in line with the PICO approach (participants, intervention, comparator and outcomes). This process was conducted by 4 reviewers (JA, AM, GE and MPV) according to the same extraction pro forma. All clinical outcome measures reported in selected studies were extracted. Data extraction included, in addition to outcomes, the number of participants, participant age group, specific robot(s) used, study setting, study design, comparators and specialty.

Duplicate reports of the same study may be present in different journals, manuscripts or conference proceedings and may each focus on different outcome measures or include a follow up data point. The data extraction process was conducted on the most comprehensive report of a given study.

### Data Synthesis and Analysis

The identified robots were grouped in this review by their predominant role. These groupings were created by the authors and are not outwardly referenced or defined by the studies from which they are identified. Data that are not clearly defined in the studies, such as robot name, were labelled “n/a”.

## Results

### Search Results

**Fig. 1:**
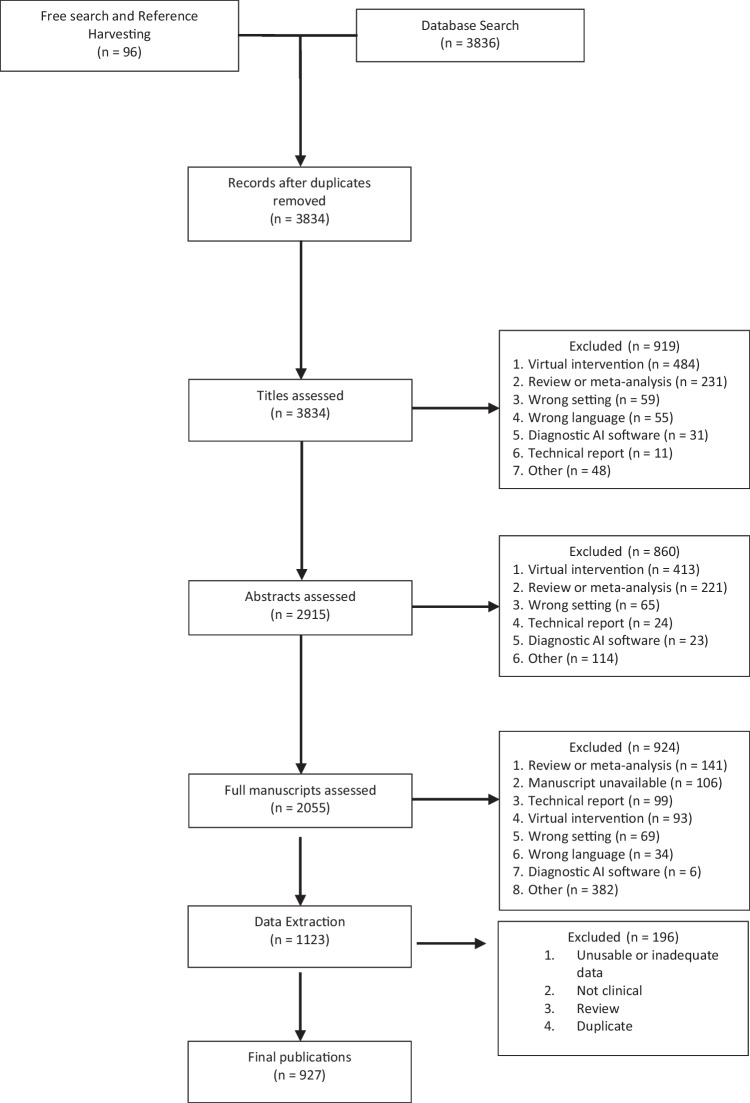
PRISMA diagram of selection process

The database search yielded 3836 publications and a further 96 were included from reference harvesting and the free search. Duplicate publications were removed (*n* = 98) and following three screening phases, 1123 publications were eligible for inclusion in the review. During data extraction, further 196 manuscripts were removed due to duplication, missing data, reviews, non-clinical evaluation with healthy participants or without enough appropriate data to extract, leaving a total of 927 original studies. The literature search is illustrated through the PRISMA flow diagram [13] in Fig. 1, which highlights the review process and reasons for exclusion.

The inter-rater agreement between the reviewers was calculated to be 0.23 for the title screen, 0.46 for the abstract screen and 0.53 the final report, demonstrating fair, moderate and moderate correlation between the reviewers respectively according to Cohen’s Kappa coefficient [14].

**Fig. 2:**
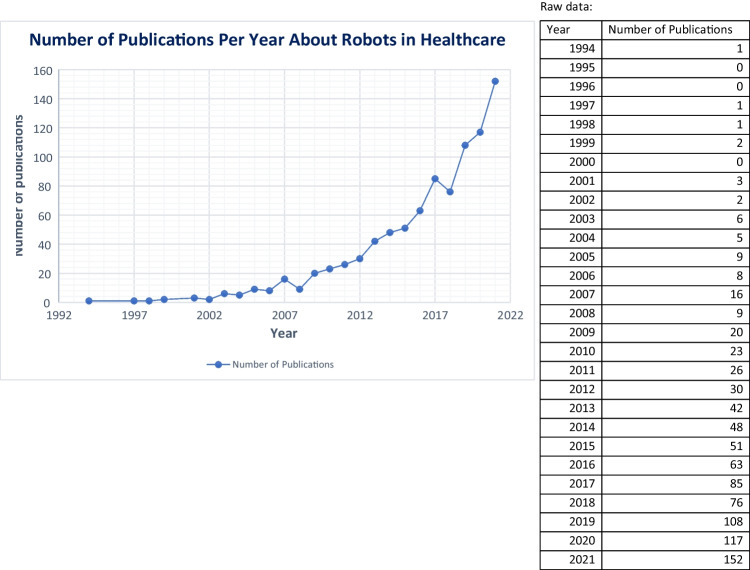
Number of publications released per year about robots in healthcare

The included studies have publication dates ranging from 1994 to 2022, with between 0 and 152 publications per year. The median number of publications per year was 16 (IQR = 46). The number of publications peaked in 2021, with the number being 585% higher than 10 years prior. The publications per year can be seen in Fig. 2. A full list of the final studies can be found in Supplementary Material B. Of the included studies, 65% were observational. The name of the robot evaluated was not clearly stated in 19% of publications. Of these, 89% were surgical robots.

### Participants and Settings

A total of 5,173,190 participants were included in the studies. Fifty-three percent of publications included fewer than 45 participants, with the larger populations generally coming from publications that analysed data from national databases. Eighty-nine percent of the manuscripts focused on adult populations, with only 7% solely including paediatrics. The specialties with most publications were stroke (*n* = 194, 21%), urology (*n* = 149, 16%) and general surgery (*n* = 137, 15%).

A range of clinical settings was used, but the two most common were the surgical theatre (*n* = 498) and the rehabilitation unit (*n* = 353). Catheterisation labs (*n* = 17), pharmacies (*n* = 16) and general wards (*n* = 10) were next in line. The remaining 4% of publications included elderly care units (*n* = 7), outpatient clinics (*n* = 6) and pathology labs (*n* = 4). Table 1 provides a further breakdown of settings.

**Table 1:** Number of publications that explored each setting

Setting	Number of publications
Theatre	498
Rehabilitation unit	353
Cath lab	17
Pharmacy	16
Ward	10
Elderly care unit	7
Clinic	6
n/a	5
Pathology lab	4
ICU	3
ED	3
General hospital	2
Stroke unit	1
Neonatal unit	1
Diagnostic imaging centre	1

Footnote: n/a refers to papers that do not clearly identify the study setting

### Identified Robots and Their Roles in Healthcare

One hundred and seventy-one named robots were identified. The da Vinci Surgical System (Intuitive Surgical, USA) was most frequently studied (*n* = 291); the Lokomat® (Hocoma, Switzerland) (*n* = 72) and Hybrid Assistive Limb (HAL) (Cyberdyne, Japan) (*n* = 46) followed. A list of all identified and named robots can be found in Supplementary Material C.

**Fig. 3:**
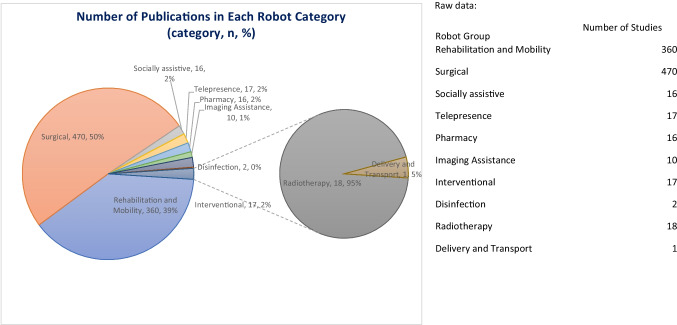
The number of studies in each of the 10 robot groups

The identified robots were categorised by their role, leading to the formation of 10 different groups. These groups represent the 10 overarching roles that robots have been found to have within healthcare. Table 2 summarises the robot groups, the number of robots found in each and the most common robot(s). Figure 3 shows the number of publications within each robot group.

**Table 2:** The 10 robot groups identified and the most common robot in each

Robot group	Description	Total number of named robots (excluding n/a)	Most frequently studied robot(s)
Rehabilitation and mobility	Robots used to physically assist or assess patients to aid in achieving a goal	102	Lokomat® (Hocoma, Switzerland)
Surgical	Robots used to assist in performing surgical procedures	19	da Vinci Surgical System (Intuitive Surgical, USA)
Telepresence	Robots that allow individuals to have a remote presence through means of the robot	10	Remote Presence (RP) (InTouch Technologies, USA)
Pharmacy	Robots that assist with the management and delivery of pharmacy services	10	APOTECA Chemo (Loccioni Humancare, Italy); ROWA Vmax (BD Rowa, Germany)
Socially assistive	Robots that take multiple forms, such as humanoid or animal, to provide support in areas classically done by humans such as companionship and service provision	9	Paro (AIST, Japan)
Interventional	Robots used to assist with interventional procedures	9	Niobe (Stereotaxis, USA)
Imaging assistance	Robots used for their ability to assist in carrying out imaging in different areas of medicine	8	Soloassist® (AKTORmed, Germany); Freehand® (Freehand, UK)
Disinfection	Robots used to disinfect clinical areas such as the ward or outpatient clinic	2	LightStrike™ (Xenex, USA); Ultra Violet Disinfection Robot® (UVD-Robot) (Clean Room Solutions)
Radiotherapy	Robots used to assist with delivery of radiotherapy	1	Cyberknife (Accuray, USA)
Delivery and transport	Robots used for the transfer of items between areas	1	TUG (Aethon, USA)

#### Surgical

Surgical robots can be used to assist in performing surgical procedures. Their specific roles within surgery are varied, ranging from instrument control to automated surgical table movement. This is a well-explored role, making up 51% of included studies and with 19 named robots identified. Most studies within this category are observational in nature (90%).

The da Vinci Surgical System is the predominant robot in use and thus has the largest literature base behind it. The system provides instruments that can be controlled by a surgeon through a console to perform minimally invasive surgery. It can be used in procedures including cholecystectomy, pancreatectomy and prostatectomy. For example, Jensen et al. [15] carried out a retrospective cohort study with 103 patients and compared robot assisted anti-reflux surgery with the da Vinci Surgical system to conventional laparoscopy and evaluated peri-operative outcomes. Other robotic systems that have been studied include the ROBODOC® Surgical System (Curexo Technology, USA) which was used in orthopaedics to plan and carry out total knee arthroplasties, [16] and Robotized Stereotactic Assistant (ROSA®) (Zimmer Biomet, France) which can assist with neurosurgical procedures such as intracranial electrode implantation [17].

Some of the identified robots can also assist with biopsy. For example, the iSR’obot™ Mona Lisa (Biobot Surgical, Singapore) can assist with visualisation and robotic needle guidance in prostate biopsy. One included publication studied this robot prospectively in a group of 86 men undergoing prostate biopsy with the researchers primarily evaluating detection of clinically significant prostate cancer [18].

#### Rehabilitation and Mobility

Rehabilitation and mobility robots are those that can physically assist or assess patients to aid in achieving goals. They can function to improve dexterity, achieve rehabilitation targets or aid in mobilisation. These robots may be used in the inpatient setting as well as in community rehabilitation centres. Rehabilitation is one of the major roles of robots in healthcare, making up 39% of reviewed manuscripts. This group of robots had the highest proportion of interventional studies, with 75% of all interventional studies originating from this group.

There are 102 named robots within this group, and they can be used for a variety of functions. Most are used for their ability to provide physical support to patients, assisting with rehabilitation. This can include single-joint or whole-body support. Others may be used for posture training through robotic tilt tables or for mobilisation through robotic wheelchairs.

The most common robot, Lokomat®, is a gait orthosis robot that can be used for rehabilitation in disorders such as stroke. Its primary role is to increase lower limb strength and range of motion. One study that evaluated this robot came from Husemann et al. [19] who carried out a randomised controlled trial with 30 acute stroke patients and compared those receiving conventional physiotherapy alone to those receiving conventional plus Lokomat therapy and evaluated outcomes such as ambulation ability. The second most studied robot, HAL, is a powered exoskeleton with multiple variants including a lower limb and single-joint version. Studies predominantly explore its use in neurological rehabilitation, but research is also present in areas such as post-operative rehabilitation.

Two studies showed robots being used to evaluate different patient parameters, such as gait speed. Hunova (Movendo Technology, Italy) is a robot that can be used for trunk and lower limb rehabilitation but can also be used for sensorimotor assessment such as limits of stability. An example of this robot being used was demonstrated by Cella et al. [20] who utilised the robot to obtain patient parameters that could be used in a fall risk assessment model within the elderly community, with the idea that robotic assessment can augment clinical evaluation and provide more robust data.

#### Radiotherapy

Radiotherapy robots can be used to assist with delivery of radiotherapy. This review identified one robot in this group: Cyberknife (Accuray, USA) (*n* = 18). This robot can assist with application of radiotherapy and image guidance to manage conditions such as liver and orbital metastases. All publications were observational with no comparator groups. One such publication was from Staehler et al. [21] who carried out a prospective case–control trial with 40 patients with renal tumour and evaluated safety and efficacy of Cyberknife use.

#### Telepresence

A core feature of the telepresence robotic group is the ability to allow individuals to have a remote presence through means of the robot. The robot may be used for activities such as remote ward rounds, remote surgical mentoring or remote assessment of histology slides. This group included 17 publications with the most common robots being remote presence (RP) (InTouch Technologies, USA) and Double (Double Robotics, USA). Double is a self-driving robot with two wheels and a video interface. Croghan et al. [22] used this robot for surgical ward rounds with a remote consultant surgeon and compared the experience to conventional ward rounds.

#### Interventional

Separate from their surgical counterpart, robots from this group are used to assist with interventional procedures. This includes procedures such as ablation in atrial fibrillation, percutaneous coronary intervention (PCI) and neuro-endovascular intervention. Their function can range from catheter guidance to stent positioning. There were 17 publications included that cover nine robots, with the most common being the Niobe System (Stereotaxis, USA) and Hansen Sensei Robotic Catheter System (Hansen Medical, USA), followed by the Corpath systems (GRX and 200) (Corindus, USA). The Niobe system uses robotically controlled magnets to allow for catheter direction. Arya et al. [23] carried out a case–control study comparing the Niobe system with conventional manual catheter navigation and evaluated effectiveness and safety in managing atrial fibrillation. The Corpath 200 system has been used for procedures such as PCI, [24] with robotic catheter guidance and the GRX system has also been reported to be used in endo-neurovascular procedures [25•].

#### Socially Assistive

Socially assistive robots can take multiple forms, such as humanoid or animal-like, and work to provide support in areas traditionally done by humans such as companionship and service provision. Nine robots across 16 studies were included with the most popular being PARO (AIST, Japan) followed by Pepper (SoftBank Robotics, Japan) and NAO (SoftBank Robotics, Japan). PARO is a robotic seal that can move and make sounds in addition to responding to stimuli. Hung et al. [26] studied dementia patient perception of PARO on the hospital ward and its potential benefits. Pepper is a humanoid robot with a touch screen, capable of interacting with people through conversation. Boumans et al. [27] explored the use of Pepper in outpatient clinics with a randomised clinical trial. They compared human and Pepper-mediated patient interviews and evaluated patient perception following this.

#### Pharmacy

There are a group of robots with the specific role of assisting with the management and delivery of pharmacy services. This includes drug storage, dispensing and compounding. For example, a robot may assist in preparation of cytotoxic drugs with the goal of reducing errors and minimising operator risk. Sixteen manuscripts with 10 robots were included. BD Rowa™ Vmax (BD Rowa, Germany) and APOTECA Chemo (Loccioni Humancare, Italy) were the most frequently studied robots. The BD Rowa™ Vmax is an automated system that allows for storage of medication and dispensing at the request of a user. Berdot et al. [28] used this system in a teaching hospital pharmacy and evaluated the return on investment including the rate of dispensing errors. The APOTECA Chemo system can be used to automate the production of chemotherapeutic treatment. Buning et al. [29] explored the environmental contamination of APOTECA Chemo compared to conventional drug compounding.

#### Imaging Assistance

Robots in this group have been specifically used for their ability to assist in carrying out imaging in different areas of medicine. Ten publications were included, with 8 robots in total. They predominantly include robotic camera holders in theatre but can also include robotic microscopes in neurosurgery and transcranial magnetic stimulation robots. Soloassist® (AKTORmed, Germany) and Freehand® (Freehand, UK), robotic camera controllers, were the most common in literature. Robotic camera holders may be controlled by various inputs such as voice and a joystick. In one publication, Soloassist was compared to a human scope assistant in colorectal cancer and safety and feasibility were assessed [30].

#### Disinfection

Robots may be used to disinfect clinical areas such as the ward or outpatient clinic. This group included 2 studies that evaluated the robotic systems LightStrike™ (Xenex, USA) and Ultra Violet Disinfection Robot® (UVD-Robot) (Clean Room Solutions). Both systems use ultraviolet (UV) light for disinfection of rooms, with the UVD-R being able to move autonomously. UVD-R was explored by Astrid et al. [31] who analysed its ability to disinfect waiting rooms in hospital outpatient clinics and compared this to conventional manual disinfection.

#### Delivery and Transport

There exists a role for robots in the transfer of items between areas. One publication was included that explored a delivery robot in the intensive care unit (ICU) [32]. The TUG Automated Delivery System (Aethon, USA) is a robot that after being loaded by an operator was used to autonomously deliver drugs from the pharmacy department to the ICU.

## Discussion

### Evaluation of Robots in Clinical Settings

There has been an explosion of publications about the use of robots in healthcare in the past few years. This coincides with the COVID-19 pandemic, which highlighted a need for robots to carry out roles in challenging environments. It can also be linked with the ongoing development of technologies and the promise of robots alleviating the healthcare works’ burden and improving patient outcomes. The successful implementation of a robotic system is multifactorial, driven by social need, regulatory approval and the financial impact of deploying the system. Once introduced into healthcare, the durability and ongoing use of the robot are difficult to predict. Certain systems may go on to see long-term use, whilst others are underutilised or removed from practice. The outcome may be related to ease of use, perceived and objective benefit or availability of a newer system. Following successful introduction, robotic systems go on to be used for a variety of roles.

Ten overarching roles for robots in healthcare were identified in this review: surgical, rehabilitation and mobility, radiotherapy, socially assistive, telepresence, pharmacy, disinfection, delivery and transport, interventional and imaging assistance. In each group, robots may have different sub-roles, such as a focus on upper limb or lower limb strengthening in the rehabilitation category or for drug compounding or dispensing within the pharmacy category. These 10 groups have been created to consolidate a variety of robots, but it should be noted that there is an overlap between them as a robot may have multiple functions. For example, the low-intensity collimated ultrasound (LICU) system is categorised as an interventional robot with the primary role of ablation in conditions such as atrial fibrillation [33•]. However, it also involves automated ultrasound (US) imaging which overlaps with the imaging assistance group. These roles allow robots to be used across a range of healthcare settings.

Certain robot groups have a well-defined area of use. For instance, the surgical group is unsurprisingly found predominantly within the hospital theatre setting. However, other robot groups are not so restricted to a well-circumscribed area. The pharmacy and socially assistive group of robots are such examples, which can be found in both inpatient and outpatient settings. Although numerous environments have been identified, most publications evaluated robots within only two: the theatre and rehabilitation unit. Robots have been less well explored in other settings, such as ED and ICU. This may be because some environments are more unpredictable, with fewer repetitive tasks that are well suited for a robot. The use of robots in more challenging and less controlled environments is a potential area for further research.

No matter the setting or role of the robot, a similar benefit is found with all robotic systems. They allow for a task to be carried out with less direct involvement of a human. The socially assistive and telepresence groups are good examples of this. This means that robots can be used in situations where services are needed but with restrictions on human presence. For instance, COVID-19 provides a clear example of where telepresence robots may be used to safely conduct remote ward rounds.

### Quality of Selected Studies

This review did not exclude publications based on quality of methodology. Most studies were observational, with the interventional design being mainly used with rehabilitation and mobility robots. Many studies included in this review are also descriptive, with retrospectively defined outcomes. This highlights a need for further high-quality interventional studies to establish the potential benefits of robots across a range of roles. Additionally, a large portion of studies, outside of those using national databases, is of a small sample size. This, combined with the observational nature, reduces the overall quality of the dataset.

### Review Strengths and Limitations

Review strengths include the large number of publications analysed and broad scope of the subject. This large dataset provides a comprehensive overview of the field of robotics in healthcare, and for synthesis of the data to establish the main robot roles in practice. As no limit was placed on date of publication, trends can also be established.

Given the broad area of exploration, there is a risk of missing relevant studies. Although many robots have been included, there will be some used in clinical practice that have not been identified by this review. However, it is unlikely that the missing robots will have a major impact on the 10 robot groups identified, given the substantial number of papers reviewed.

Several robots have multiple editions, but these were counted as singular entities, precluding more detailed analysis of each edition. Additionally, some publications did not specify the name of the robot used, and so there may be unique robots that were not identified in this review. For the same reason, some robots may be more commonly studied than described in this review. However, given the significant disparity in number of publications behind the predominant robots and those below them, the big picture is unlikely to drastically change. Finally, it should also be noted that there is a possibility of overlapping patient populations, with some studies utilising similar datasets.

### Future of Robotics

The future of robots in healthcare predominantly lies with remote presence, and the performance of tasks detached from human presence. For instance, safe disinfection of a clinical environment or ward rounds with an at home specialist. Robots will allow for people to be present with increasing flexibility. This will aid in providing consistent services that are resilient to change and easy to adapt. For instance, a well-established robotic system that allows for remote surgery or telepresence ward rounds could mean that care can continue to be provided in a consistent manner during a pandemic.

To fully realise a future of widespread robot adoption, the necessary infrastructure must be developed. The best robotic system may be foiled by a poor internet connection. Investment in the systems that allow robots to operate is vital. The adoption of certain robot groups is also more likely to be seen due to the barriers of implementation. A socially assistive robot that moves on two wheels is likely much cheaper and easier to implement, especially in areas with fewer resources, compared to a large drug dispensing or surgical robot. Therefore, these more complex robots may struggle to see widespread use. It is important to focus on robots that are more likely to be globally utilised and have far-reaching effects, especially with scarcity of human resources. This is even more important when in crisis.

With ongoing technological advancements, robots may also be developed to carry out new functions. The roles described in this review arise from robots that have been used in a current clinical setting, but there are robots in development or pre-clinical evaluation that may yet be introduced. Advancement in the areas of artificial intelligence may lead to socially assistive robots that can function more independently and perform more complex tasks. Evolving technology such as augmented reality with haptic feedback may also provide a new scope for telepresence, such as remote physical guidance during a complex procedure.

Generally, there is a need to further evaluate the financial and clinical impact of robots with high-quality studies, larger population groups and an interventional design where possible. A need also exists to evaluate the use of robots in different populations and settings.

## Conclusion

The evidence base for the use of robots in healthcare is expanding, and robots are being used across a range of specialties and settings. Ten overall roles for robots were identified, with the best explored being surgical and rehabilitation roles. However, there is a need for further high-quality research, particularly with less well-established robot roles such as disinfection. The future of robots lies in remote presence and the ability to carry out tasks in challenging environments; this will depend on the development of robust infrastructure and network capabilities to allow for successful adoption.

## Supplementary Information

Below is the link to the electronic supplementary material.

Supplementary file1 (DOCX 12 KB) **Supplement A.** Search string used.

Supplementary file2 (DOCX 138 KB) **Supplement B.** List of all included studies, organised by date of publication.

Supplementary file3 (DOCX 35 KB) **Supplement C.** List of all named robots identified, a brief description, the category of robot, and number of publications that explored the robot. Sorted by most to least commonly studied. Excluding robots that were not clearly named in the study.
