# Supplementary material for: Robots in Healthcare: a Scoping Review
Source: Curr Robot Rep. 2022 Oct 22;3(4):271–80. doi: 10.1007/s43154-022-00095-4 (PMC9589563; doi:10.1007/s43154-022-00095-4)
Supplement: Supplementary file 1 — Supplementary file1 (DOCX 12 KB) Supplement A. Search string used. [file 43154_2022_95_MOESM1_ESM.docx]

**Supplement A**

Title: Search string.

1. Service robot* [Text word]

2. Surgical robot* [Text word]

3. Da Vinci surgical system [Text word]

4. Socially assistive robot* [Text word]

5. Paro [Text word]

6. NAO [Text word]

7. Training robot* [Text word]

8. Robotics [MeSH]

9. 1 or 2 or 3 or 4 or 5 or 6 or 7 or 8

10. Inpatient setting [Text word]

11. Outpatient setting [Text word]

12. Pharmacy [Text word]

13. Trauma centre [Text word]

14. Acute centre [Text word]

15. Rehabilitation hospital* [Text word]

16. Geriatric hospital* [Text word]

17. Field hospital* [Text word]

18. Hospitals [MeSH]

19. 10 or 11 or 12 or 13 or 14 or 15 or 16 or 17 or 18

20. 9 and 19
