# Supplementary material for: Robots in Healthcare: a Scoping Review
Source: Curr Robot Rep. 2022 Oct 22;3(4):271–80. doi: 10.1007/s43154-022-00095-4 (PMC9589563; doi:10.1007/s43154-022-00095-4)
Supplement: Supplementary file 2 — Supplementary file2 (DOCX 138 KB) Supplement B. List of all included studies, organised by date of publication. [file 43154_2022_95_MOESM2_ESM.docx]

Supplement B

Title: List of all included studies, organised by date of publication.

| Publication Year | Study Title | DOI |
| --- | --- | --- |
| 1994 | A computer-aided walking rehabilitation robot | 10.1097/00002060-199406000-00011 |
| 1997 | The effect of robot-assisted therapy and rehabilitative training on motor recovery following stroke | 10.1001/ARCHNEUR.1997.00550160075019 |
| 1998 | Robot-aided neurorehabilitation | 10.1109/86.662623 |
| 1999 | Telepathology between Richmond and Beckley Veterans Affairs Hospitals: Report on the first 1000 cases | 10.1089/107830299311934 |
| 1999 | Routine surgical telepathology in the Department of Veterans Affairs: Experience-related improvements in pathologist performance in 2200 cases | 10.1089/107830299311899 |
| 2001 | Feasibility of robotic laparoscopic surgery: 146 cases | 10.1007/S00268-001-0132-2 |
| 2001 | Telerobotic laparoscopic cholecystectomy: Initial clinical experience with 25 patients | 10.1097/00000658-200107000-00001 |
| 2001 | Image-guided radiosurgery in the treatment of spinal metastases. | 10.3171/FOC.2001.11.6.7 |
| 2002 | Robotic assisted kidney transplantation: An initial experience | 10.1016/s0022-5347(05)65162-2 |
| 2002 | Feasibility of Robot-Assisted Laparoscopic Surgery | 10.1097/00129689-200202000-00007 |
| 2003 | Robotics in general surgery: Personal experience in a large community hospital | 10.1001/ARCHSURG.138.7.777 |
| 2003 | DaVinci® Robotic-Assisted Laparoscopic Bariatric Surgery: Is it Justified in a Routine Setting? | 10.1381/096089203322618632 |
| 2003 | Effects of robotic therapy on motor impairment and recovery in chronic stroke | 10.1053/APMR.2003.50110 |
| 2003 | Evaluation of CyberKnife Frameless Real-Time Image-Guided Stereotactic Radiosurgery for Spinal Lesions | 10.1159/000075109 |
| 2003 | CyberKnife frameless single-fraction stereotactic radiosurgery for benign tumors of the spine. | 10.3171/FOC.2003.14.5.17 |
| 2003 | CyberKnife frameless single-fraction stereotactic radiosurgery for tumors of the sacrum. | 10.3171/FOC.2003.15.2.7 |
| 2004 | Robotic and laparoscopic surgery for treatment of colorectal diseases | 10.1007/S10350-004-0711-Z |
| 2004 | Robotic therapy for chronic motor impairments after stroke: Follow-up results | 10.1016/j.apmr.2003.11.028 |
| 2004 | Comparison of two techniques of robot-aided upper limb exercise training after stroke | 10.1097/01.PHM.0000137313.14480.CE |
| 2004 | Robot-assisted mediastinal parathyroidectomy. | 10.1007/S00464-003-4272-3 |
| 2004 | The robotic, 2-stage, 3-field esophagolymphadenectomy | 10.1016/J.JTCVS.2004.02.014 |
| 2005 | Establishment of the world's first telerobotic remote surgical service: For provision of advanced laparoscopic surgery in a rural community | 10.1097/01.SLA.0000154456.69815.EE |
| 2005 | Novel uses of surgical robotics in head and neck surgery | 10.1089/lap.2005.15.647 |
| 2005 | Telerobotic-assisted laparoscopic cholecystectomy: Our experience on 29 patients | 10.1007/S00534-004-0932-5 |
| 2005 | Advantages and limits of robot-assisted laparoscopic surgery: Preliminary experience | 10.1007/S00464-004-9004-9 |
| 2005 | Computer-assisted laparoscopic pyeloplasty: University of Miami experience with the daVinci™ surgical system | 10.1089/END.2005.19.387 |
| 2005 | Telementoring facilitates independent hand-assisted laparoscopic living donor nephrectomy | 10.1016/j.transproceed.2005.01.065 |
| 2005 | Effectiveness of automated locomotor training in patients with chronic incomplete spinal cord injury: A multicenter trial | 10.1016/J.APMR.2004.08.004 |
| 2005 | Robot-assisted laparoscopic dismembered pyeloplasty: A combined experience | 10.1089/END.2005.19.382 |
| 2005 | Robot-aided sensorimotor arm training methods based on neurological rehabilitation principles in stroke and brain injury patients | 10.1109/IEMBS.2005.1615604 |
| 2006 | A novel robot device in rehabilitation of post-stroke hemiplegic upper limbs | 10.1007/BF03324854 |
| 2006 | Clinical characteristics of remote Zeus robot-assisted laparoscopic cholecystectomy: A report of 40 cases | 10.3748/WJG.V12.I16.2606 |
| 2006 | Evaluation of da Vinci Nissen fundoplication clinical results and cost minimization | 10.1007/S00268-005-7950-6 |
| 2006 | Changes in Motoneuron Excitability in Hemiplegic Subjects After Passive Exercise When Using a Robotic Arm | 10.1016/J.APMR.2006.05.026 |
| 2006 | Robot-assisted thoracoscopic esophagectomy with the patient in the prone position | 10.1089/LAP.2006.16.278 |
| 2006 | Cyberknife radiosurgery for basal skull plasmacytoma | 10.1111/J.1552-6569.2006.00062.X |
| 2006 | MIME robotic device for upper-limb neurorehabilitation in subacute stroke subjects: A follow-up study | 10.1682/JRRD.2005.02.0044 |
| 2006 | Robot-assisted laparoscopic and open live-donor nephrectomy: A comparison of donor morbidity and early renal allograft outcomes | 10.1093/NDT/GFI150 |
| 2007 | Remote telepresence surgery: The Canadian experience | 10.1007/S00464-006-9040-8 |
| 2007 | Robot-assisted laparoscopic myomectomy versus abdominal myomectomy: A comparison of short-term surgical outcomes and immediate costs | 10.1016/J.JMIG.2007.06.008 |
| 2007 | Laparoscopic tubal reanastomosis using robotics: Experience from a teaching institution | 10.1089/LAP.2006.0035 |
| 2007 | Robotic fundoplication in children: resident teaching and a single institutional review of our first 50 patients | 10.1016/j.jpedsurg.2007.08.022 |
| 2007 | Results of total hip replacement using the Robodoc surgical assistant system: Clinical outcome and evaluation of complications for 97 procedures | 10.1002/rcs.161 |
| 2007 | Telerobotic-assisted laparoscopic abdominoperineal resection for low rectal cancer: Report of the first case in Hong Kong and China with an updated literature review | 10.3748/WJG.V13.I17.2514 |
| 2007 | Robotic distal pancreatectomy and nephrectomy for living donor pancreas-kidney transplantation | 10.1097/01.TP.0000284585.12633.06 |
| 2007 | Computer-enhanced robotic surgery in gynecologic oncology | 10.1007/s00464-006-0894-6 |
| 2007 | Prospective, blinded, randomized crossover study of gait rehabilitation in stroke patients using the Lokomat gait orthosis | 10.1177/1545968307300697 |
| 2007 | Robotic-Assisted Rehabilitation of the Upper Limb After Acute Stroke | 10.1016/J.APMR.2006.10.032 |
| 2007 | Robotic Telepresence: Profit Analysis in Reducing Length of Stay after Laparoscopic Gastric Bypass | 10.1016/J.JAMCOLLSURG.2007.01.070 |
| 2007 | Effects of locomotion training with assistance of a robot-driven gait orthosis in hemiparetic patients after stroke: A randomized controlled pilot study | 10.1161/01.STR.0000254607.48765.CB |
| 2007 | Transoral robotic surgery: Radical tonsillectomy | 10.1001/ARCHOTOL.133.12.1220 |
| 2007 | Deployment and early experience with remote-presence patient care in a community hospital | 10.1007/S00464-005-0261-Z |
| 2007 | Curative stereotactic robotic radiotherapy treatment for extracranial, extrapulmonary, extrahepatic, and extraspinal tumors: Technique, early results, and toxicity | 10.1177/153303460700600603 |
| 2007 | Effect of a robotic prescription-filling system on pharmacy staff activities and prescription-filling time | 10.2146/ajhp060561 |
| 2008 | A pilot study of activity-based therapy in the arm motor recovery post stroke: A randomized controlled trial | 10.1177/0269215508095358 |
| 2008 | Robotic equipment malfunction during robotic prostatectomy: A multi-institutional study | 10.1089/END.2007.0407 |
| 2008 | Robotic hysterectomy and pelvic-aortic lymphadenectomy for endometrial cancer | 10.1097/AOG.0B013E31818E4416 |
| 2008 | A Comparison of Total Laparoscopic Hysterectomy to Robotically Assisted Hysterectomy: Surgical Outcomes in a Community Practice | 10.1016/J.JMIG.2008.01.008 |
| 2008 | Comparison of robot-assisted laparoscopic adrenalectomy with traditional laparoscopic adrenalectomy - 1 Year follow-up | 10.1007/S00464-007-9488-1 |
| 2008 | Remote magnetic versus manual catheter navigation for ablation of supraventricular tachycardias: A randomized, multicenter trial | 10.1111/J.1540-8159.2008.01183.X |
| 2008 | The effect of the GENTLE/s robot-mediated therapy system on arm function after stroke | 10.1177/0269215507085060 |
| 2008 | Early experiences of robotic-assisted laparoscopic liver resection | 10.3349/YMJ.2008.49.4.632 |
| 2008 | Robot-assisted laparoscopic gastrectomy with D2 dissection for adenocarcinoma: Initial experience with 17 patients | 10.1007/S11701-008-0116-4 |
| 2009 | Multicenter randomized clinical trial evaluating the effectiveness of the Lokomat in subacute stroke | 10.1177/1545968308326632 |
| 2009 | Robotic Surgery: Changing the Surgical Approach for Endometrial Cancer in a Referral Cancer Center | 10.1016/J.JMIG.2009.03.013 |
| 2009 | Improvement of walking abilities after robotic-assisted locomotion training in children with cerebral palsy | 10.1136/ADC.2008.145458 |
| 2009 | Robotic resection of intraductal neoplasm of the pancreas | 10.1089/LAP.2009.0164 |
| 2009 | The Effectiveness of Locomotor Therapy Using Robotic-Assisted Gait Training in Subacute Stroke Patients: A Randomized Controlled Trial | 10.1016/J.PMRJ.2009.03.009 |
| 2009 | Primary frozen section diagnosis by robotic microscopy and virtual slide telepathology: the University Health Network experience | 10.1016/J.HUMPATH.2009.04.012 |
| 2009 | Effects of robotic-aided rehabilitation on recovery of upper extremity function in chronic stroke: A single case study | 10.1002/OTI.280 |
| 2009 | Spinal decompression sickness presenting as partial brown-sequard syndrome and treated with robotic-assisted body-weight support treadmill training | 10.2340/16501977-0279 |
| 2009 | Robotic neurological surgery applications: Accuracy and consistency or pure fantasy? | 10.1159/000202974 |
| 2009 | The EndoAssist™ robotic camera holder as an aid to the introduction of laparoscopic colorectal surgery | 10.1308/003588409X392162 |
| 2009 | Robotic surgical telepathology between the Iron Mountain and Milwaukee Department of Veterans Affairs Medical Centers: a 12-year experience | 10.1016/J.HUMPATH.2009.04.007 |
| 2009 | Ablation of atrial fibrillation utilizing robotic catheter navigation in comparison to manual navigation and ablation: Single-center experience | 10.1111/J.1540-8167.2009.01570.X |
| 2009 | Patient outcomes in the acute recovery phase following robotic-assisted prostate surgery: A prospective study | 10.1016/J.IJNURSTU.2007.07.010 |
| 2009 | Quality of life improvement after robotically assisted coronary artery bypass grafting | 10.1159/000212115 |
| 2009 | Robotic magnetic navigation for ablation of human arrhythmias: Initial experience | 10.1016/J.ACVD.2009.02.009 |
| 2009 | Robot therapy for functional recovery of the upper limbs: A pilot study on patients after stroke | 10.2340/16501977-0402 |
| 2009 | Functional outcomes after transoral robotic surgery for head and neck cancer | 10.1016/J.OTOHNS.2009.05.014 |
| 2009 | A multiinstitutional experience with robotic-assisted hysterectomy with staging for endometrial cancer | 10.1097/AOG.0B013E3181AF2A74 |
| 2009 | Robot-mediated therapy for paretic upper limb of chronic patients following neurological injury | 10.2340/16501977-0403 |
| 2009 | Impact of robotic dispensing machines in German pharmacies on business performance indicators | 10.4176/090731 |
| 2010 | Comparing robot-assisted with conventional laparoscopic hysterectomy: Impact on cost and clinical outcomes | 10.1016/J.JMIG.2010.06.009 |
| 2010 | Transoral robotic resection of recurrent nasopharyngeal carcinoma | 10.1002/LARY.21059 |
| 2010 | Robotically assisted hysterectomy in patients with large uteri: Outcomes in five community practices | 10.1097/AOG.0B013E3181CF45AD |
| 2010 | Robotic versus standard laparoscopy for the treatment of endometriosis | 10.1016/J.FERTNSTERT.2010.04.031 |
| 2010 | Robot-Assisted Laparoscopic Presacral Neurectomy: Feasibility, Techniques, and Operative Outcomes | 10.1016/J.JMIG.2010.03.017 |
| 2010 | The effect of the approach to radical prostatectomy on the profitability of hospitals and surgeons | 10.1111/J.1464-410X.2009.08996.X |
| 2010 | Incidence of Surgical Site Infection Associated with Robotic Surgery | 10.1086/654006 |
| 2010 | Robotic Gait Training in an Adult With Cerebral Palsy: A Case Report | 10.1016/J.PMRJ.2009.10.0120 |
| 2010 | Robotic-assisted single-incision laparoscopic partial cecectomy | 10.1002/RCS.346 |
| 2010 | Enhancing robotic gait training via augmented feedback | 10.1109/IEMBS.2010.5627707 |
| 2010 | Surgery-related Complications in 1253 Robot-assisted and 485 Open Retropubic Radical Prostatectomies at the Karolinska University Hospital, Sweden | 10.1016/J.UROLOGY.2009.09.075 |
| 2010 | Outcome and quality of life in a prospective cohort of the first 100 robotic surgeries for endometrial cancer, with focus on elderly patients | 10.1111/IGC.0B013E3181F2950A |
| 2010 | The minimally invasive approach, laparoscopic and robotic, in rectal resection for cancer. A single center experience. | 10.2298/ACI1003029P |
| 2010 | A comparison between robotic-assisted and manual implantation of cementless total hip arthroplasty | 10.1007/S11999-009-1158-2 |
| 2010 | Robot-assisted complete excision of choledochal cyst type I, hepaticojejunostomy and extracorporeal Roux-en-y anastomosis: A case report and review literature | 10.1186/1477-7819-8-87 |
| 2010 | Robot-assisted vessel harvesting for penile revascularization | 10.1111/J.1743-6109.2009.01462.X |
| 2010 | Persistence of pulmonary vein isolation after robotic remote-navigated ablation for atrial fibrillation and its relation to clinical outcome | 10.1111/J.1540-8167.2010.01773.X |
| 2010 | Continence, potency and oncological outcomes after robotic-assisted radical prostatectomy: Early trifecta results of a high-volume surgeon | 10.1111/J.1464-410X.2010.09541.X |
| 2010 | Prospective Randomized Controlled Trial of Robotic versus Open Radical Cystectomy for Bladder Cancer: Perioperative and Pathologic Results | 10.1016/J.EURURO.2009.10.024 |
| 2010 | Robotic-assisted treadmill therapy improves walking and standing performance in children and adolescents with cerebral palsy | 10.1016/J.EJPN.2010.01.002 |
| 2010 | Radiosurgery of liver tumors: Value of robotic radiosurgical device to treat liver tumors | 10.1245/S10434-010-1187-9 |
| 2010 | "Orthobot, to your station!" The application of the remote presence robotic system in orthopaedic surgery in Ireland: A pilot study on patient and nursing staff satisfaction | 10.1007/S11701-010-0207-X |
| 2010 | Laparobotic duodenal diverticulectomy and choledochoduodenostomy: a case study and review of the literature. | 10.1007/s11701-009-0167-1 |
| 2011 | Robot-assisted upper-limb therapy in acute rehabilitation setting following stroke: Department of veterans affairs multisite clinical trial | 10.1682/JRRD.2010.04.0062 |
| 2011 | Combined transcranial direct current stimulation and robot-assisted gait training in patients with chronic stroke: A preliminary comparison | 10.1177/0269215510389497 |
| 2011 | Results of clinicians using a therapeutic robotic system in an inpatient stroke rehabilitation unit | 10.1186/1743-0003-8-50 |
| 2011 | Cancer control, continence, and potency after laparoscopic radical prostatectomy beyond the learning and discovery curves | 10.1089/END.2010.0451 |
| 2011 | Robotics applied in laparoscopic kidney surgery: The Yonsei University experience of 127 cases | 10.1016/J.UROLOGY.2010.02.011 |
| 2011 | Single-port robotic cholecystectomy: Results from a first human use clinical study of the new da vinci single-site surgical platform | 10.1001/ARCHSURG.2011.143 |
| 2011 | Retraining of interjoint arm coordination after stroke using robot-assisted time-independent functional training | 10.1682/JRRD.2010.04.0064 |
| 2011 | Robot-aided therapy for upper limbs in patients with stroke-related lesions. Brief report of a clinical experience | 10.1186/1743-0003-8-18 |
| 2011 | Robot-assisted gait training for patients with hemiparesis due to stroke | 10.1310/TSR1803-269 |
| 2011 | Who may benefit from robotic-assisted gait training?: A randomized clinical trial in patients with subacute stroke | 10.1177/1545968311401034 |
| 2011 | A short-term cost-effectiveness study comparing robot-assisted laparoscopic and open retropubic radical prostatectomy | 10.3111/13696998.2011.586621 |
| 2011 | Locomotor training using a robotic device in patients with subacute spinal cord injury | 10.1038/SC.2011.59 |
| 2011 | Robot-assisted laparoscopic hysterectomy in obese and morbidly obese women: Surgical technique and comparison with open surgery | 10.1111/J.1600-0412.2011.01253.X |
| 2011 | Catheter ablation of atrial fibrillation using remote magnetic catheter navigation: A casecontrol study | 10.1093/EUROPACE/EUQ344 |
| 2011 | Robotic hepatobiliary and pancreatic surgery: A cohort study | 10.1007/S00534-011-0389-2 |
| 2011 | Robotic Radical Prostatectomy at a teaching community hospital:Outcomes and safety | 10.4293/108680811X13022985131930 |
| 2011 | Effect of a robotic rehabilitation device on upper limb function in a sub-acute cervical spinal cord injury population | 10.1109/ICORR.2011.5975400 |
| 2011 | Use of pharmacy delivery robots in intensive care units | 10.2146/AJHP100012 |
| 2011 | A prospective trial comparing consecutive series of open retropubic and robot-assisted laparoscopic radical prostatectomy in a centre with a limited caseload | 10.1016/J.EURURO.2010.10.026 |
| 2011 | Exploring the impact of an automated prescription-filling device on community pharmacy technician workflow | 10.1331/JAPHA.2011.09166 |
| 2011 | Robotic-assisted laparoscopic repair of a vesicouterine fistula | 10.4293/108680811X13071180407438 |
| 2011 | Robotic ureterolysis for relief of ureteral obstruction from retroperitoneal fibrosis | 10.1016/J.UROLOGY.2010.11.025 |
| 2011 | Multimodality laparoscopic liver resection for hepatic malignancy - From conventional total laparoscopic approach to robot-assisted laparoscopic approach | 10.1016/J.IJSU.2011.02.004 |
| 2011 | Robotic image-guided reirradiation of lateral pelvic recurrences: Preliminary results | 10.1186/1748-717X-6-77 |
| 2011 | Single-institution experience on robot-assisted thoracoscopic operations for mediastinal diseases | 10.1097/IMI.0B013E318235B783 |
| 2011 | Anatomic differences after robotic-assisted radical prostatectomy and open prostatectomy: Implications for radiation field design | 10.1016/J.PRRO.2010.11.007 |
| 2012 | An observational report of intensive robotic and manual gait training in sub-acute stroke | 10.1186/1743-0003-9-13 |
| 2012 | Robot assisted surgery in gynaecologic oncology - starting a program and initial learning curve from a UK tertiary referral centre: The Guildford perspective | 10.1002/rcs.1461 |
| 2012 | Learning curve for robot-assisted Roux-en-Y gastric bypass | 10.1007/s00464-011-2008-3 |
| 2012 | Single-site robotic cholecystectomy (SSRC) versus single-incision laparoscopic cholecystectomy (SILC): Comparison of learning curves. First European experience | 10.1007/S00464-011-2087-1 |
| 2012 | Learning experience using the double-console da Vinci surgical system in gynecology: A prospective cohort study in a University hospital | 10.1007/S00404-011-2005-8 |
| 2012 | Worldwide experience with the robotic navigation system in catheter ablation of atrial fibrillation: Methodology, efficacy and safety | 10.1111/j.1540-8167.2012.02316.x |
| 2012 | Vaginal cuff dehiscence in robotic-assisted total hysterectomy | 10.4293/108680812X13462882736817 |
| 2012 | Robotic thyroidectomy: An initial experience with the gasless transaxillary approach | 10.1089/LAP.2010.0380 |
| 2012 | Robot-assisted Laparoscopic Partial Nephrectomy: Step-by-step Contemporary Technique and Surgical Outcomes at a Single High-volume Institution | 10.1016/j.eururo.2012.05.021 |
| 2012 | Uterine preservation in pelvic organ prolapse using robot assisted laparoscopic sacrohysteropexy: Quality of life and technique | 10.1016/J.EJOGRB.2012.07.025 |
| 2012 | Qualitative and quantitative differences between 2 robotic thyroidectomy techniques | 10.1177/0194599812439283 |
| 2012 | Comparative analysis of outcomes and costs following open radical cystectomy versus robot-assisted laparoscopic radical cystectomy: Results from the US Nationwide Inpatient Sample | 10.1016/J.EURURO.2012.03.032 |
| 2012 | Individual finger synchronized robot-assisted hand rehabilitation in subacute to chronic stroke: A prospective randomized clinical trial of efficacy | 10.1177/0269215511431473 |
| 2012 | Comparative effectiveness of robotic versus laparoscopic hysterectomy for endometrial cancer | 10.1200/JCO.2011.36.7508 |
| 2012 | Single-incision robotic-assisted living donor nephrectomy: Case report and description of surgical technique | 10.1111/J.1432-2277.2012.01493.X |
| 2012 | Use of robotics in spinal cord injury: A case report | 10.5014/AJOT.2012.000943 |
| 2012 | Transvaginal hybrid natural orifice transluminal surgery robotic donor nephrectomy: First clinical application | 10.1016/J.UROLOGY.2012.08.061 |
| 2012 | Off-clamp robot-assisted partial nephrectomy: Initial Washington University experience | 10.1089/END.2012.0094 |
| 2012 | Robotic resistance treadmill training improves locomotor function in human spinal cord injury: A pilot study | 10.1016/j.apmr.2011.12.018 |
| 2012 | Comparative hospital cost-analysis of open and robotic-assisted radical prostatectomy | 10.1016/J.UROLOGY.2012.03.020 |
| 2012 | Robotic versus conventional laparoscopic surgery for rectal cancer: A cost analysis from a single institute in Korea | 10.1007/S00268-012-1728-4 |
| 2012 | Novel robotic catheter manipulation system integrated with remote magnetic navigation for fully remote ablation of atrial tachyarrhythmias: A two-centre evaluation | 10.1093/EUROPACE/EUS169 |
| 2012 | Robot-assisted laparoscopic hemi-hepatectomy: Technique and surgical outcomes | 10.1016/J.IJSU.2011.10.005 |
| 2012 | Impact of robotic antineoplastic preparation on safety, workflow, and costs | 10.1200/JOP.2012.000600 |
| 2012 | Lokomat Robotic-Assisted Versus Overground Training Within 3 to 6 Months of Incomplete Spinal Cord Lesion: Randomized Controlled Trial | 10.1177/1545968312448232 |
| 2012 | Biochemical recurrence after robot-assisted radical prostatectomy in a european single-centre cohort with a minimum follow-up time of 5 years | 10.1016/J.EURURO.2012.05.024 |
| 2012 | Robot-assisted gait training in multiple sclerosis patients: A randomized trial | 10.1177/1352458511431075 |
| 2012 | Peri-operative outcomes of patients with stage IV endometriosis undergoing robotic-assisted laparoscopic surgery | 10.1007/S11701-011-0314-3 |
| 2012 | Surgical telepresence: The usability of a robotic communication platform | 10.1186/1749-7922-7-S1-S11 |
| 2012 | Retroperitoneal robotic-assisted laparoscopic reimplantation of a ureter into an ileal conduit | 10.1007/S11701-011-0286-3 |
| 2013 | Impact of robotic operative efficiency on profitability | 10.1016/J.AJOG.2013.03.030 |
| 2013 | Operative outcomes of robot-assisted transaxillary thyroid surgery for benign thyroid disease: Early experience in 50 patients | 10.1007/S00423-013-1085-2 |
| 2013 | Robotic versus laparoscopic adrenalectomy: A comparative study in a high-volume center | 10.1007/S00464-012-2496-9 |
| 2013 | Comparison of robotic, laparoscopic, and abdominal myomectomy in a community hospital | 10.4293/108680812X13517013317473 |
| 2013 | Robot-assisted versus open radical prostatectomy: The differential effect of regionalization, procedure volume and operative approach | 10.1016/J.JURO.2012.10.028 |
| 2013 | Hand-assisted laparoscopic versus robot-assisted laparoscopic partial nephrectomy: Comparison of short-term outcomes and cost | 10.1089/end.2012.0210 |
| 2013 | Impact of the introduction of a robotic training programme on prostate cancer stage migration at a single tertiary referral centre | 10.1111/j.1464-410X.2012.11464.x |
| 2013 | Unilateral versus bilateral robot-assisted rehabilitation on arm-trunk control and functions post stroke: A randomized controlled trial | 10.1186/1743-0003-10-35 |
| 2013 | Robot-assisted reconstructive surgery of the distal ureter: Single institution experience in 16 patients | 10.1111/J.1464-410X.2012.11673.X |
| 2013 | Robotic Assisted Surgery in Pediatric Gynecology: Promising Innovation in Mini Invasive Surgical Procedures | 10.1016/J.JPAG.2012.09.009 |
| 2013 | Feasibility of rehabilitation training with a newly developed wearable robot for patients with limited mobility | 10.1016/J.APMR.2012.12.020 |
| 2013 | Leg surface electromyography patterns in children with neuro-orthopedic disorders walking on a treadmill unassisted and assisted by a robot with and without encouragement | 10.1186/1743-0003-10-78 |
| 2013 | Atypical autonomic dysreflexia during robotic-assisted body weight supported treadmill training in an individual with motor incomplete spinal cord injury | 10.1179/2045772312Y.0000000033 |
| 2013 | Effects of robotic treadmill training on functional mobility, walking capacity, motor symptoms and quality of life in ambulatory patients with Parkinson's disease: A preliminary prospective longitudinal study | 10.3233/NRE-130962 |
| 2013 | Robotic-assisted minimally invasive vs. Thoracoscopic lung lobectomy: Comparison of perioperative results in a learning curve setting | 10.1007/S00423-013-1090-5 |
| 2013 | Short-term outcomes for robotic colorectal surgery by provider volume | 10.1016/J.JAMCOLLSURG.2013.07.390 |
| 2013 | Oncological outcomes: Open vs robotic prostatectomy | 10.1111/J.1464-410X.2012.11638.X |
| 2013 | Robotic-assisted Roux-en-Y gastric bypass: Update from 2 high-volume centers | 10.1016/J.SOARD.2011.11.022 |
| 2013 | Robotic-assisted colorectal surgery in the United States: A nationwide analysis of trends and outcomes | 10.1007/S00268-013-2024-7 |
| 2013 | Does robot-assisted gait training ameliorate gait abnormalities in multiple sclerosis? A pilot randomized-control trial | 10.3233/NRE-130990 |
| 2013 | Pilot study of a robotic protocol to treat shoulder subluxation in patients with chronic stroke | 10.1186/1743-0003-10-88 |
| 2013 | Clinical effects of using HEXORR (Hand Exoskeleton Rehabilitation Robot) for movement therapy in stroke rehabilitation | 10.1097/PHM.0B013E31829E7A07 |
| 2013 | Single institutional cost analysis of 325 robotic, laparoscopic, and open partial nephrectomies | 10.1016/J.UROLOGY.2012.07.104 |
| 2013 | Combination of robot-assisted and conventional body-weight-supported treadmill training improves gait in persons with multiple sclerosis: A pilot study | 10.1097/NPT.0000000000000018 |
| 2013 | Assessment of an automatic robotic arm for dispensing of chemotherapy in a 2500-bed medical center | 10.1016/J.JFMA.2011.11.026 |
| 2013 | Perioperative complications of robot-assisted partial nephrectomy: Analysis of 886 patients at 5 United States centers | 10.1016/J.UROLOGY.2012.10.067 |
| 2013 | Outcomes and costs associated with robotic colectomy in the minimally invasive era | 10.1097/DCR.0B013E31827085EC |
| 2013 | Hospitalization costs for radical prostatectomy attributable to robotic surgery | 10.1016/J.EURURO.2012.08.012 |
| 2013 | Parapharyngeal space surgery via a transoral approach using a robotic surgical system: Transoral robotic surgery | 10.1089/LAP.2012.0197 |
| 2013 | Implementation of an i.v.-compounding robot in a hospital-based cancer center pharmacy | 10.2146/AJHP120649 |
| 2013 | The Psychosocial Effects of a Companion Robot: A Randomized Controlled Trial | 10.1016/J.JAMDA.2013.02.007 |
| 2013 | Preliminary trial of postural strategy training using a personal transport assistance robot for patients with central nervous system disorder | 10.1016/J.APMR.2012.08.208 |
| 2013 | Risk and prevention of acute urinary retention after robotic prostatectomy | 10.1016/J.JURO.2012.09.097 |
| 2013 | Trends in immediate perioperative morbidity and delay in discharge after open and minimally invasive radical prostatectomy (RP): A 20-year institutional experience | 10.1111/J.1464-410X.2012.11767.X |
| 2013 | Oncologic, functional, and complications outcomes of robot-assisted radical cystectomy with totally intracorporeal neobladder diversion | 10.1016/J.EURURO.2013.05.050 |
| 2013 | Robotic versus laparoscopic coloanal anastomosis with or without intersphincteric resection for rectal cancer | 10.1007/s00464-013-3014-4 |
| 2013 | Investigation of robotic-assisted tilt-table therapy for early-stage spinal cord injury rehabilitation | 10.1682/JRRD.2012.02.0027 |
| 2013 | Intravenous tissue plasminogen activator administration in community hospitals facilitated by telestroke service | 10.1227/NEU.0000000000000073 |
| 2013 | Application of arm support training in sub-acute stroke rehabilitation: First results on effectiveness and user experiences | 10.1109/ICORR.2013.6650470 |
| 2013 | A Newly Developed Robot Suit Hybrid Assistive Limb Facilitated Walking Rehabilitation after Spinal Surgery for Thoracic Ossification of the Posterior Longitudinal Ligament: A Case Report | 10.1155/2013/621405 |
| 2013 | A single-center study of 100 consecutive patients with localized prostate cancer treated with stereotactic body radiotherapy | 10.1186/1471-2490-13-49 |
| 2013 | The impact of automation on pharmacy staff experience of workplace stressors | 10.1111/J.2042-7174.2012.00231.X |
| 2014 | Reliability of robotic system during general surgical procedures in a university hospital | 10.1016/J.AMJSURG.2013.06.007 |
| 2014 | Learning curve for robot-assisted neck dissection in head and neck cancer: A 3-year prospective case study and analysis | 10.1001/jamaoto.2014.2830 |
| 2014 | First report on joint use of a Da Vinci® surgical system with transfer of surgical know-how between two public hospitals | 10.1159/000360301 |
| 2014 | Learning curve assessment of robot-assisted radical prostatectomy compared with open-surgery controls from the premier perspective database | 10.1089/END.2013.0534 |
| 2014 | Robotic Retroperitoneal Paraaortic Lymphadenectomy at Donostia University Hospital | 10.1016/J.JMIG.2013.10.004 |
| 2014 | Robotic upper limb rehabilitation after acute stroke by NeReBot: Evaluation of treatment costs | 10.1155/2014/265634 |
| 2014 | Three-dimensional, task-specific robot therapy of the arm after stroke: A multicentre, parallel-group randomised trial | 10.1016/S1474-4422(13)70305-3 |
| 2014 | Robotic therapy provides a stimulus for upper limb motor recovery after stroke that is complementary to and distinct from conventional therapy | 10.1177/1545968313510974 |
| 2014 | Robotic-assisted partial nephrectomy in duplicated collecting systems in the pediatric population: Techniques and outcomes | 10.1016/J.JPUROL.2013.10.014 |
| 2014 | Short-term and long-term outcomes of serial robotic training for improving upper limb function in chronic stroke | 10.1097/MRR.0000000000000036 |
| 2014 | Application of a robot for critical care rounding in small rural hospitals | 10.1016/J.CCELL.2014.08.006 |
| 2014 | A multinational, multi-institutional study comparing positive surgical margin rates among 22 393 open, laparoscopic, and robot-assisted radical prostatectomy patients | 10.1016/J.EURURO.2013.11.018 |
| 2014 | A pilot study of post-total knee replacement gait rehabilitation using lower limbs robot-assisted training system | 10.1007/S00590-012-1159-9 |
| 2014 | The association between experience and proficiency with robotic-enhanced coronary intervention-insights from the PRECISE multi-center study | 10.3109/17482941.2014.889314 |
| 2014 | Evolution of robotic nephrectomy for living donation: From hand-assisted to totally robotic technique | 10.1002/RCS.1576 |
| 2014 | Strength training versus robot-assisted gait training after incomplete spinal cord injury: A randomized pilot study in patients depending on walking assistance | 10.1186/1743-0003-11-4 |
| 2014 | Modular ankle robotics training in early subacute stroke: A randomized controlled pilot study | 10.1177/1545968314521004 |
| 2014 | Propensity-matched comparison of morbidity and costs of open and robot-assisted radical cystectomies: A contemporary population-based analysis in the United States | 10.1016/J.EURURO.2014.01.029 |
| 2014 | Therapeutic synergism in the treatment of post-stroke arm paresis utilizing botulinum toxin, robotic therapy, and constraint-induced movement therapy | 10.1016/J.PMRJ.2014.04.014 |
| 2014 | Isometric hip and knee torque measurements as an outcome measure in robot assisted gait training | 10.3233/NRE-131042 |
| 2014 | Gait training with a robotic leg brace after stroke: A randomized controlled pilot study | 10.1097/PHM.0000000000000119 |
| 2014 | Patient-specific determinants of responsiveness to robot-enhanced treadmill therapy in children and adolescents with cerebral palsy | 10.1111/DMCN.12564 |
| 2014 | Locomotion improvement using a hybrid assistive limb in recovery phase stroke patients: A randomized controlled pilot study | 10.1016/J.APMR.2014.07.002 |
| 2014 | Robot-assisted gait training is not superior to balance training for improving postural instability in patients with mild to moderate Parkinson's disease: A single-blind randomized controlled trial | 10.1177/0269215514544041 |
| 2014 | Predicting clinically significant changes in motor and functional outcomes after robot-assisted stroke rehabilitation | 10.1016/J.APMR.2013.09.018 |
| 2014 | Robotic resection of huge presacral tumors: Case series and comparison with an open resection | 10.1097/BSD.0B013E318299C5FD |
| 2014 | An economic analysis of robotically assisted hysterectomy | 10.1097/AOG.0000000000000244 |
| 2014 | bilateral robotic priming before task-oriented approach in subacute stroke rehabilitation: A pilot randomized controlled trial | 10.1016/j.ejpn.2014.04.012 |
| 2014 | Radical prostatectomy: Initial experience with robot-assisted laparoscopic procedures at a large university hospital | 10.3109/21681805.2013.868514 |
| 2014 | Is minimally invasive colon resection better than traditional approaches? First comprehensive national examination with propensity score matching | 10.1001/JAMASURG.2013.3660 |
| 2014 | Does robotic distal pancreatectomy surgery offer similar results as laparoscopic and open approach? A comparative study from a single medical center | 10.1002/RCS.1569 |
| 2014 | Applications for transoral robotic surgery in the pediatric airway | 10.1002/LARY.24753 |
| 2014 | Robotic ablation of atrial fibrillation with a new remote catheter system | 10.1007/S10840-014-9895-X |
| 2014 | Comparing robot-assisted thoracic surgical lobectomy with conventional video-assisted thoracic surgical lobectomy and wedge resection: Results from a multihospital database (Premier) | 10.1016/J.JTCVS.2013.09.046 |
| 2014 | Robotic Coronary artery bypass grafting decreases 30-day complication rate, length of stay, and acute care facility discharge rate compared with conventional surgery | 10.1097/IMI.0000000000000095 |
| 2014 | Concomitant robotic mitral and tricuspid valve repair: Technique and early experience | 10.1016/J.ATHORACSUR.2013.09.049 |
| 2014 | Effect on arm function and cost of robot-assisted group therapy in subacute patients with stroke and a moderately to severely affected arm: A randomized controlled trial | 10.1177/0269215513516967 |
| 2014 | Transoral robotic surgery and the unknown primary: A cost-effectiveness analysis | 10.1177/0194599814525746 |
| 2014 | Complex robotic-enhanced percutaneous coronary intervention | 10.1002/CCD.25271 |
| 2014 | Robotic gastrectomy versus laparoscopic gastrectomy for gastric cancer: Comparison of surgical performance and short-term outcomes | 10.1007/S00464-013-3385-6 |
| 2014 | Robotic vs open simple enucleation for the treatment of T1a-T1b renal cell carcinoma: A single center matched-pair comparison | 10.1016/J.UROLOGY.2013.08.080 |
| 2014 | Initial results of robot-assisted thoracoscopic surgery in Japan | 10.1007/S11748-014-0441-7 |
| 2014 | Recovery of hand function with robot-assisted therapy in acute stroke patients: A randomized-controlled trial | 10.1097/MRR.0000000000000059 |
| 2014 | Training current and future robotic surgeons simultaneously: Initial experiences with safety and efficiency | 10.1007/S11701-014-0455-2 |
| 2014 | Beginning robotic assisted colorectal surgery – it’s harder than it looks! | 10.5114/WIITM.2014.45494 |
| 2014 | Laparoscopic robotic liver surgery: The Henri Mondor initial experience of 20 cases | 10.1007/S11701-013-0437-9 |
| 2014 | Robot-assisted vs. sensory integration training in treating gait and balance dysfunctions in patients with multiple sclerosis: A randomized controlled trial | 10.3389/FNHUM.2014.00318 |
| 2014 | Robotic versus traditional laparoscopic partial nephrectomy: Comparison of outcomes with a transition of techniques | 10.1007/S11701-013-0447-7 |
| 2015 | Modifications of transaxillary approach in endoscopic da Vinci-assisted thyroid and parathyroid gland surgery | 10.1007/S11701-014-0486-8 |
| 2015 | Robot-assisted Simple Prostatectomy for Treatment of Lower Urinary Tract Symptoms Secondary to Benign Prostatic Enlargement: Surgical Technique and Outcomes in a High-volume Robotic Centre | 10.1016/J.EURURO.2015.03.003 |
| 2015 | Implementation of a robotic surgical program in gynaecological oncology and comparison with prior laparoscopic series | 10.1155/2015/814315 |
| 2015 | Single- versus dual-console robot-assisted radical prostatectomy: impact on intraoperative and postoperative outcomes in a teaching institution | 10.1007/S00345-014-1349-7 |
| 2015 | Implementing robotic surgery to gynecologic oncology: The first 300 operations performed at a tertiary hospital | 10.1111/AOGS.12620 |
| 2015 | Robot-Assisted Training Early after Cardiac Surgery | 10.1111/JOCS.12576 |
| 2015 | The learning curve for robotic distal pancreatectomy: An analysis of outcomes of the first 100 consecutive cases at a high-volume pancreatic centre | 10.1111/hpb.12412 |
| 2015 | Transaxillary thyroidectomies: A comparative learning experience of robotic vs endoscopic thyroidectomies | 10.1177/0194599815573003 |
| 2015 | Robotic assisted Roux-en-Y hepaticojejunostomy in a post-cholecystectomy type E2 bile duct injury | 10.3748/WJG.V21.I6.1703 |
| 2015 | Transoral robotic surgery in management of oropharyngeal cancers: a preliminary experience at a tertiary cancer centre in India | 10.1007/S10147-014-0774-3 |
| 2015 | Robotic distal pancreatectomy versus conventional laparoscopic distal pancreatectomy: a comparative study for short-term outcomes | 10.1007/S11684-015-0404-0 |
| 2015 | Robotic gait training improves motor skills and quality of life in hereditary spastic paraplegia | 10.3233/NRE-141196 |
| 2015 | Comparing open radical cystectomy and robot-assisted laparoscopic radical cystectomy: A randomized clinical trial | 10.1016/j.eururo.2014.11.043 |
| 2015 | Robotic liver surgery: preliminary experience in a tertiary hepato-biliary unit | 10.1007/S13304-015-0285-4 |
| 2015 | Utilization and costs associated with robotic surgery in children | 10.1016/J.JSS.2015.04.087 |
| 2015 | Standardized analysis of complications after robot-assisted radical cystectomy: Korea university hospital experience | 10.4111/KJU.2015.56.1.48 |
| 2015 | Utilizing telemedicine in the trauma intensive care unit: Does it impact teamwork? | 10.1089/TMJ.2014.0074 |
| 2015 | A comparison of robotic surgery in children weighing above and below 15.0 kg: size does not affect surgery success | 10.1007/S00464-014-3982-Z |
| 2015 | Robotic therapy for chronic stroke: General recovery of impairment or improved task-specific skill? | 10.1152/JN.00336.2015 |
| 2015 | Critical outcomes in nonrobotic vs robotic-assisted cardiac surgery | 10.1001/JAMASURG.2015.1098 |
| 2015 | Do post-stroke patients benefit from robotic verticalization? A pilot-study focusing on a novel neurophysiological approach | 10.3233/RNN-140475 |
| 2015 | A Randomized Controlled Trial of EEG-Based Motor Imagery Brain-Computer Interface Robotic Rehabilitation for Stroke | 10.1177/1550059414522229 |
| 2015 | Improved walking ability with wearable robot-assisted training in patients suffering chronic stroke1 | 10.3233/BME-151320 |
| 2015 | Use of a robotic device for the rehabilitation of severe upper limb paresis in subacute stroke: Exploration of patient/robot interactions and the motor recovery process | 10.1155/2015/482389 |
| 2015 | Robot-assisted surgery in cervical cancer patients reduces the time to normal activities of daily living | 10.1111/AOGS.12561 |
| 2015 | Concurrent use of a robotic uterine manipulator and a robotic laparoscope holder to achieve assistant-less solo laparoscopy: the double ViKY | 10.1007/S11701-015-0518-Z |
| 2015 | Evolution from laparoscopic to robotic nephron sparing surgery: a high-volume laparoscopic center experience on achieving ‘trifecta’ outcomes | 10.1007/S00345-015-1552-1 |
| 2015 | Transperitoneal versus extraperitoneal robot-assisted laparoscopic radical prostatectomy: A prospective single surgeon randomized comparative study | 10.1111/IJU.12854 |
| 2015 | Outcomes of open, laparoscopic, and robotic abdominoperineal resections in patients with rectal cancer | 10.1097/DCR.0000000000000475 |
| 2015 | Wrist Rehabilitation Assisted by an Electromyography-Driven Neuromuscular Electrical Stimulation Robot after Stroke | 10.1177/1545968314565510 |
| 2015 | Functional outcomes, feasibility, and safety of resection of transoral robotic surgery: Single-institution series of 35 consecutive cases of transoral robotic surgery for oropharyngeal squamous cell carcinoma | 10.1002/HED.23809 |
| 2015 | In-hospital mortality and morbidity after robotic coronary artery surgery | 10.1053/J.JVCA.2014.03.009 |
| 2015 | Non-randomised comparison of acute and long-term outcomes of robotic versus manual ventricular tachycardia ablation in a single centre ischemic cohort | 10.1007/S10840-015-9992-5 |
| 2015 | Impact of catheter ablation with remote magnetic navigation on procedural outcomes in patients with persistent and long-standing persistent atrial fibrillation | 10.1007/S10840-015-0037-X |
| 2015 | Trends in Robotic Thyroid Surgery in the United States from 2009 Through 2013 | 10.1089/THY.2015.0066 |
| 2015 | Robot-Assisted Gait Training in a Patient With Hereditary Spastic Paraplegia | 10.1016/J.PMRJ.2014.09.008 |
| 2015 | Robotic colorectal surgery for laparoscopic surgeons with limited experience: Preliminary experiences for 40 consecutive cases at a single medical center Visceral and general surgery | 10.1186/S12893-015-0057-6 |
| 2015 | Robotic tilt table reduces the occurrence of orthostatic hypotension over time in vegetative states | 10.1097/MRR.0000000000000104 |
| 2015 | Comparison of complications of robot-assisted laparoscopic and open appendicovesicostomy in children | 10.1016/J.JURO.2015.02.2942 |
| 2015 | Robotic mitral valve repair for simple and complex degenerative disease | 10.1161/CIRCULATIONAHA.115.017792 |
| 2015 | Robotic total gastrectomy with intracorporeal robot-sewn anastomosis: A novel approach adopting the double-loop reconstruction method | 10.1097/MD.0000000000001922 |
| 2015 | Oncologic Long-Term Results of Robot-Assisted Minimally Invasive Thoraco-Laparoscopic Esophagectomy with Two-Field Lymphadenectomy for Esophageal Cancer | 10.1245/s10434-015-4544-x |
| 2015 | Physiological effects of a companion robot on blood pressure of older people in residential care facility: A pilot study | 10.1111/AJAG.12099 |
| 2015 | Minimally invasive kidney transplantation: Perioperative considerations and key 6-month outcomes | 10.1097/TP.0000000000000590 |
| 2015 | Variation in pelvic lymph node dissection among patients undergoing radical prostatectomy by hospital characteristics and surgical approach: Results from the national cancer database | 10.1016/J.JURO.2014.09.019 |
| 2015 | Cribriform adenocarcinoma of the tongue and minor salivary gland: Transoral robotic surgical resection | 10.1159/000375535 |
| 2015 | HAL® exoskeleton training improves walking parameters and normalizes cortical excitability in primary somatosensory cortex in spinal cord injury patients | 10.1186/S12984-015-0058-9 |
| 2015 | Robotic telepresence versus standardly supervised stroke alert team assessments | 10.1089/tmj.2014.0064 |
| 2015 | Reduced knee hyperextension after wearing a robotic knee orthosis during gait training - a case study | 10.3233/BME-151326 |
| 2015 | Single fraction radiosurgery for the treatment of renal tumors | 10.1016/J.JURO.2014.08.044 |
| 2015 | Effects of robot-assisted gait training on the balance and gait of chronic stroke patients: Focus on dependent ambulators | 10.1589/JPTS.27.3053 |
| 2016 | Outcomes and Complications of Robotic-assisted Laparoscopic Prostatectomy in a Community Hospital Setting | 10.1016/J.UROLOGY.2016.05.060 |
| 2016 | Non-invasive brain stimulation and robot-assisted gait training after incomplete spinal cord injury: A randomized pilot study | 10.3233/NRE-151291 |
| 2016 | Total robotic radical rectal resection with da Vinci Xi system: single docking, single phase technique | 10.1002/RCS.1734 |
| 2016 | A comparison of robotic single-incision and traditional single-incision laparoscopic cholecystectomy | 10.1007/S00464-015-4223-9 |
| 2016 | Patient exposure to extremely low-frequency magnetic fields during laparoscopic and robotic surgeries | 10.1002/RCS.1686 |
| 2016 | Robotic Retroperitoneal Lymph Node Dissection in Advanced Stage Disease | 10.1159/000450924 |
| 2016 | The impact of robotic cholecystectomy on private practice in a community teaching hospital | 10.1016/J.AMJSURG.2015.11.010 |
| 2016 | Robotically assisted treadmill exercise training for improving peak fitness in chronic motor incomplete spinal cord injury: A randomized controlled trial | 10.1179/2045772314Y.0000000281 |
| 2016 | Introducing robotic surgery into an endometrial cancer service-a prospective evaluation of clinical and economic outcomes in a UK institution | 10.1002/RCS.1651 |
| 2016 | Effects on mobility training and de-adaptations in subjects with Spinal Cord Injury due to a Wearable Robot: A preliminary report | 10.1186/S12883-016-0536-0 |
| 2016 | Environmental contamination by cyclophosphamide preparation: Comparison of conventional manual production in biological safety cabinet and robot-assisted production by APOTECAchemo | 10.1177/1078155214551316 |
| 2016 | Artificial urinary sphincter implantation in women with stress urinary incontinence: preliminary comparison of robot-assisted and open approaches | 10.1007/s00192-015-2858-7 |
| 2016 | Sequencing bilateral robot-assisted arm therapy and constraint-induced therapy improves reach to press and trunk kinematics in patients with stroke | 10.1186/S12984-016-0138-5 |
| 2016 | Conflicting results of robot-assisted versus usual gait training during postacute rehabilitation of stroke patients: A randomized clinical trial | 10.1097/MRR.0000000000000137 |
| 2016 | The effects of robot-assisted gait training in progressive multiple sclerosis: A randomized controlled trial | 10.1177/1352458515620933 |
| 2016 | Return to work and normal daily life activity after open and robot-assisted radical prostatectomy - A single surgeon analysis | 10.1159/000437335 |
| 2016 | Laparoscopic versus robotic-assisted Roux-en-Y gastric bypass: a retrospective, single-center study of early perioperative outcomes at a community hospital | 10.1007/S00464-015-4675-Y |
| 2016 | Locomotor training through a novel robotic platform for gait rehabilitation in pediatric population: short report | 10.1186/S12984-016-0206-X |
| 2016 | Tailor-made rehabilitation approach using multiple types of hybrid assistive limb robots for acute stroke patients: A pilot study | 10.1080/10400435.2015.1080768 |
| 2016 | Robotic-assisted radical prostatectomy learning curve for experienced laparoscopic surgeons: Does it really exist? | 10.1590/S1677-5538.IBJU.2014.0485 |
| 2016 | Robot-assisted Versus Open Radical Prostatectomy: A Contemporary Analysis of an All-payer Discharge Database | 10.1016/j.eururo.2016.01.044 |
| 2016 | Transvertebral direct current stimulation paired with locomotor training in chronic spinal cord injury: A case study | 10.3233/NRE-151292 |
| 2016 | Robotic Enucleation for Benign or Borderline Tumours of the Pancreas: A Retrospective Analysis and Comparison from a High-Volume Centre in Asia | 10.1007/S00268-016-3655-2 |
| 2016 | tDCS and Robotics on Upper Limb Stroke Rehabilitation: Effect Modification by Stroke Duration and Type of Stroke | 10.1155/2016/5068127 |
| 2016 | Surgeon and Hospital Level Variation in the Costs of Robot-Assisted Radical Prostatectomy | 10.1016/J.JURO.2016.04.087 |
| 2016 | Efficacy of Upper Extremity Robotic Therapy in Subacute Poststroke Hemiplegia: An Exploratory Randomized Trial | 10.1161/STROKEAHA.115.012520 |
| 2016 | Robotic video-assisted thoracoscopic lung resection for lung tumors: a community tertiary care center experience over four years | 10.1007/s00464-015-4249-z |
| 2016 | Robotic and open distal pancreatectomy with celiac axis resection for locally advanced pancreatic body tumors: a single institutional assessment of perioperative outcomes and survival | 10.1016/J.HPB.2016.05.003 |
| 2016 | Outcomes of high-complexity renal tumours with a Preoperative Aspects and Dimensions Used for an Anatomical (PADUA) score of ≥10 after robot-assisted partial nephrectomy with a median 46.5-month follow-up: a tertiary centre experience | 10.1111/BJU.13501 |
| 2016 | Comparison of clinical and economic outcomes between robotic, laparoscopic, and open rectal cancer surgery: early experience at a tertiary care center | 10.1007/S00464-015-4390-8 |
| 2016 | Totally robotic rectal resection: an experience of the first 100 consecutive cases | 10.1007/S00384-016-2503-Z |
| 2016 | Perioperative Outcomes, Health Care Costs, and Survival After Robotic-assisted Versus Open Radical Cystectomy: A National Comparative Effectiveness Study [figure presented] | 10.1016/J.EURURO.2016.03.028 |
| 2016 | Robotic surgery with high dissection and low ligation technique for consecutive patients with rectal cancer following preoperative concurrent chemoradiotherapy | 10.1007/S00384-016-2581-Y |
| 2016 | Neural correlates of motor recovery after robot-assisted stroke rehabilitation: a case series study | 10.1080/13554794.2016.1215469 |
| 2016 | Advances in Robotic Vena Cava Tumor Thrombectomy: Intracaval Balloon Occlusion, Patch Grafting, and Vena Cavoscopy | 10.1016/J.EURURO.2016.06.024 |
| 2016 | Is outpatient robotic pyeloplasty feasible? | 10.1007/S11701-016-0577-9 |
| 2016 | Robot-assisted gait training improves brachial-ankle pulse wave velocity and peak aerobic capacity in subacute stroke patients with totally dependent ambulation Randomized controlled trial | 10.1097/MD.0000000000005078 |
| 2016 | Group activity with Paro in nursing homes: Systematic investigation of behaviors in participants | 10.1017/S1041610216000120 |
| 2016 | The role of intraoperative ultrasound in small renal mass robotic enucleation | 10.4081/AIUA.2016.4.311 |
| 2016 | Complications of the first 500 extra-peritoneal robot-assisted radical prostatectomy (EP-RARP) cases in an Italian medium volume centre | 10.5301/URO.5000172 |
| 2016 | Utilization and impact of surgical technique on the performance of pelvic lymph node dissection at radical prostatectomy: Results from the Shared Equal Access Regional Cancer Hospital database | 10.1111/IJU.13027 |
| 2016 | Robot-assisted transaxillary thyroid surgery—retrospective analysis of anthropometric features | 10.1007/S00423-016-1505-1 |
| 2016 | Initial experience with robotic pancreatic surgery: Technical feasibility and oncological implications | 10.1097/SLE.0000000000000232 |
| 2016 | Comparison of the morbidity and mortality of cystectomy and ileal conduit urinary diversion for neurogenic lower urinary tract dysfunction according to the approach: Laparotomy, laparoscopy or robotic | 10.1111/iju.13166 |
| 2016 | Robot-assisted gait training might be beneficial for more severely affected children with cerebral palsy | 10.3109/17518423.2015.1017661 |
| 2016 | Open vs robotic radical gastrectomy for locally advanced gastric cancer | 10.1002/RCS.1674 |
| 2016 | Kinematic measures for upper limb motor assessment during robot-mediated training in patients with severe sub-acute stroke | 10.3233/RNN-150565 |
| 2016 | Robotic cystogastrostomy and debridement of walled-off pancreatic necrosis | 10.1007/S11701-016-0581-0 |
| 2016 | Outcomes of complex robot-assisted extravesical ureteral reimplantation in the pediatric population | 10.1016/J.JPUROL.2015.11.007 |
| 2016 | Oncologic Outcomes of Robot-Assisted Radical Cystectomy: Results of a High-Volume Robotic Center | 10.1089/END.2015.0482 |
| 2016 | Robotic approaches may offer benefit in colorectal procedures, more controversial in other areas: a review of 168,248 cases | 10.1007/S00464-015-4327-2 |
| 2016 | Characterization of unexpected postural changes during robot-assisted gait training in paraplegic patients | 10.1038/SC.2015.138 |
| 2016 | Acceptability of Robot Assistant in Management of Type 1 Diabetes in Children | 10.1089/DIA.2015.0428 |
| 2016 | Comparison of acute kidney injury after robot-assisted laparoscopic radical prostatectomy versus retropubic radical prostatectomy a propensity score matching analysis | 10.1097/MD.0000000000002650 |
| 2016 | Robotic Partial Nephrectomy in the Treatment of Renal Angiomyolipoma | 10.1089/END.2015.0624 |
| 2016 | Total Anatomical Reconstruction during Robot-assisted Radical Prostatectomy: Implications on Early Recovery of Urinary Continence | 10.1016/J.EURURO.2015.08.005 |
| 2016 | Test-retest reliability and four-week changes in cardiopulmonary fitness in stroke patients: Evaluation using a robotics-assisted tilt table | 10.1186/S12883-016-0686-0 |
| 2016 | Oncological results at 2 years after robotic radical prostatectomy – The Romanian experience | 10.5173/CEJU.2016.640 |
| 2016 | Robot-assisted rehabilitation of ankle plantar flexors spasticity: A 3month study with proprioceptive neuromuscular facilitation | 10.3389/FNBOT.2016.00016 |
| 2016 | Long-term interventions effects of robotic training on patients after anterior cruciate ligament reconstruction | 10.1589/JPTS.28.2196 |
| 2016 | Self-Paced Reaching after Stroke: A Quantitative Assessment of Longitudinal and Directional Sensitivity Using the H-Man Planar Robot for Upper Limb Neurorehabilitation. | 10.3389/fnins.2016.00477 |
| 2016 | Effects of training with the ReWalk exoskeleton on quality of life in incomplete spinal cord injury: a single case study | 10.1038/SCSANDC.2015.25 |
| 2016 | Simultaneous Retroperitoneal Robotic Partial Nephrectomy and Hepatectomy for Synchronous Renal-Cell Carcinoma and Hepatocellular Carcinoma in a Cirrhotic Patient | 10.1089/CREN.2016.0096 |
| 2017 | Robotic Resistance Treadmill Training Improves Locomotor Function in Children With Cerebral Palsy: A Randomized Controlled Pilot Study | 10.1016/J.APMR.2017.04.022 |
| 2017 | Does training of fellows affect peri-operative outcomes of robot-assisted partial nephrectomy? | 10.1111/BJU.13901 |
| 2017 | Da Vinci robotic surgery in a pediatric hospital | 10.1089/LAP.2016.0390 |
| 2017 | Role of robotic-assisted pancreatic surgery: lessons learned from our initial experience | 10.1016/S1499-3872(17)60054-7 |
| 2017 | Evolving application of minimally invasive cancer operations at a tertiary cancer center | 10.1002/JSO.24526 |
| 2017 | Robotic-assisted minimally invasive esophagectomy for treatment of esophageal carcinoma | 10.1007/S11701-016-0644-2 |
| 2017 | Slow Versus Fast Robot-Assisted Locomotor Training After Severe Stroke: A Randomized Controlled Trial | 10.1097/PHM.0000000000000810 |
| 2017 | Early assessment of feasibility and technical specificities of transoral robotic surgery using the da Vinci Xi | 10.1007/S11701-017-0679-Z |
| 2017 | Feasibility and efficacy of a robotic device for hand rehabilitation in hemiplegic stroke patients: A randomized pilot controlled study | 10.1177/0269215516642606 |
| 2017 | The features of Gait Exercise Assist Robot: Precise assist control and enriched feedback | 10.3233/NRE-171459 |
| 2017 | Does assist-as-needed upper limb robotic therapy promote participation in repetitive activity-based motor training in sub-acute stroke patients with severe paresis? | 10.3233/NRE-171454 |
| 2017 | Robot-assisted internal mammary lymph chain excision for breast cancer | 10.1097/MD.0000000000007894 |
| 2017 | A Comparative Study of Outcomes Between Single-Site Robotic and Multi-port Laparoscopic Cholecystectomy: An Experience from a Tertiary Care Center | 10.1007/S00268-016-3799-0 |
| 2017 | Pattern of improvement in upper limb pointing task kinematics after a 3-month training program with robotic assistance in stroke | 10.1186/S12984-017-0315-1 |
| 2017 | Robotic management of gastric stromal tumors (GIST): a single Middle Eastern center experience | 10.1002/rcs.1729 |
| 2017 | Is the Use of a Robotic Camera Holder Economically Viable? A Cost Comparison of Surgical Assistant Versus the Use of a Robotic Camera Holder in Laparoscopic Liver Resections | 10.1097/SLE.0000000000000452 |
| 2017 | Laparoscopic Radical Prostatectomy with a Remote Controlled Robot | 10.1016/J.JURO.2016.10.107 |
| 2017 | A comparison of trends in operative approach and postoperative outcomes for colorectal cancer surgery | 10.1016/J.JSS.2016.09.019 |
| 2017 | Preliminary experience in laparoscopic resection of hepatic hydatidectocyst with the da Vinci Surgical System (DVSS): A case report | 10.1186/S12893-017-0294-Y |
| 2017 | Robotic Rectal Resection with a Single-docking Technique Thanks to the Rotation of the R3 Arm | 10.1097/SLE.0000000000000383 |
| 2017 | Decrease of spasticity after hybrid assistive limb® training for a patient with C4 quadriplegia due to chronic SCI | 10.1080/10790268.2016.1225913 |
| 2017 | Gait training with Hybrid Assistive Limb enhances the gait functions in subacute stroke patients: A pilot study | 10.3233/NRE-161393 |
| 2017 | Laparoscopy vs. Robotic Surgery for Endometriosis (LAROSE): a multicenter, randomized, controlled trial | 10.1016/J.FERTNSTERT.2016.12.033 |
| 2017 | Hepatobilio-pancreatic robotic surgery: initial experience from a single center institute | 10.1007/S11701-016-0663-Z |
| 2017 | Da Vinci Xi and Si platforms have equivalent perioperative outcomes during robot-assisted partial nephrectomy: preliminary experience | 10.1007/s11701-016-0612-x |
| 2017 | Robotic surgery rapidly and successfully implemented in a high volume laparoscopic center on living kidney donation | 10.1002/RCS.1743 |
| 2017 | Combined transcranial direct current stimulation and robotic upper limb therapy improves upper limb function in an adult with cerebral palsy | 10.3233/NRE-171455 |
| 2017 | Robot-Assisted Ventral Mesh Rectopexy for Rectal Prolapse: A 5-Year Experience at a Tertiary Referral Center | 10.1097/DCR.0000000000000895 |
| 2017 | Can Lokomat therapy with children and adolescents be improved? An adaptive clinical pilot trial comparing Guidance force, Path control, and FreeD | 10.1186/s12984-017-0287-1 |
| 2017 | Introduction of Robotic Surgery into a Community Hospital Setting: A Prospective Comparison of Robotic and Open Colorectal Resection for Cancer | 10.1159/000456085 |
| 2017 | When Partial Nephrectomy is Unsuccessful: Understanding the Reasons for Conversion from Robotic Partial to Radical Nephrectomy at a Tertiary Referral Center | 10.1016/J.JURO.2017.01.019 |
| 2017 | Prospective evaluation of vesicourethral anastomosis outcomes in robotic radical prostatectomy during early experience in a university hospital | 10.1590/S1677-5538.IBJU.2016.0466 |
| 2017 | Outcomes of robot-assisted simple enucleation of renal masses: A single European center experience | 10.1097/MD.0000000000006771 |
| 2017 | Against the odds: What to expect in rehabilitation of chronic spinal cord injury with a neurologically controlled Hybrid Assistive Limb exoskeleton. A subgroup analysis of 55 patients according to age and lesion level | 10.3171/2017.2.FOCUS171 |
| 2017 | The Impact of Surgeon Volume on Perioperative Outcomes and Cost for Patients Receiving Robotic Partial Nephrectomy | 10.1089/END.2017.0207 |
| 2017 | Cognitive-Motor Interference on Upper Extremity Motor Performance in a Robot-Assisted Planar Reaching Task Among Patients With Stroke | 10.1016/J.APMR.2016.12.004 |
| 2017 | Nationwide Assessment of Robotic Lobectomy for Non-Small Cell Lung Cancer | 10.1016/J.ATHORACSUR.2016.09.108 |
| 2017 | Combined treatment of botulinumtoxin and Robot-Assisted rehabilitation therapy on poststroke, upper limb spasticity | 10.1097/MD.0000000000009468 |
| 2017 | Two years of experience with robot-assisted anti-reflux surgery: A retrospective cohort study | 10.1016/J.IJSU.2017.02.014 |
| 2017 | Hospital Volume and Outcomes of Robot-Assisted Lobectomies | 10.1016/J.CHEST.2016.09.008 |
| 2017 | The affordability of minimally invasive procedures in major lung resection: A prospective study | 10.1093/ICVTS/IVX149 |
| 2017 | Robotic versus conventional laparoscopic cholecystectomy: A comparative study of medical resource utilization and clinical outcomes | 10.1016/J.KJMS.2017.01.010 |
| 2017 | Real-time intraprocedural 18F-FDG PeT/CT-guided biopsy using automated robopsy arm (ARA) in the diagnostic evaluation of thoracic lesions with prior inconclusive biopsy results: Initial experience from a tertiary health care centre | 10.1259/BJR.20170258 |
| 2017 | Robot-assisted Surgery for Benign Ureteral Strictures: Experience and Outcomes from Four Tertiary Care Institutions | 10.1016/J.EURURO.2016.07.022 |
| 2017 | Outcomes after pediatric open, laparoscopic, and robotic pyeloplasty at academic institutions | 10.1016/J.JPUROL.2016.08.029 |
| 2017 | Transcutaneous Vagus Nerve Stimulation Combined with Robotic Rehabilitation Improves Upper Limb Function after Stroke | 10.1155/2017/7876507 |
| 2017 | Outcome one year after robot-assisted rectal cancer surgery: a consecutive cohort study | 10.1007/S00384-017-2880-Y |
| 2017 | Bilateral robotic priming before task-oriented approach in subacute stroke rehabilitation: A pilot randomized controlled trial | 10.1177/0269215516633275 |
| 2017 | Results of the first interim analysis of the RAPPER II trial in patients with spinal cord injury: Ambulation and functional exercise programs in the REX powered walking aid | 10.1186/S12984-017-0274-6 |
| 2017 | Robotic splenectomy with ex vivo bench surgery and hemi-spleen autotransplant: the first report | 10.1007/s11701-016-0635-3 |
| 2017 | Robot-Assisted Harvesting of Kidneys for Transplantation and Global Complications for the Donor | 10.1016/J.TRANSPROCEED.2017.02.038 |
| 2017 | The robotic approach for enucleation of a giant esophageal lipoma | 10.1007/S11701-016-0668-7 |
| 2017 | Robot-assisted complete thymectomy for mediastinal ectopic parathyroid adenomas in primary hyperparathyroidism | 10.1007/S11701-016-0637-1 |
| 2017 | A nationwide evaluation of robotic ventral hernia surgery | 10.1016/J.AMJSURG.2017.08.022 |
| 2017 | Bilateral Axillo-Breast Approach Robotic Thyroidectomy (BABA RT) Does Not Interfere with Breast Image Follow-Up | 10.1007/S00268-017-3997-4 |
| 2017 | Anorectal complications after robotic intersphincteric resection for low rectal cancer | 10.1007/s00464-017-5499-8 |
| 2017 | A Pragmatic Randomized Controlled Trial Examining the Impact of the Retzius-sparing Approach on Early Urinary Continence Recovery After Robot-assisted Radical Prostatectomy | 10.1016/J.EURURO.2017.04.029 |
| 2017 | Is the future of coronary arterial revascularization a hybrid approach?: The Canadian experience across three centers | 10.1097/IMI.0000000000000355 |
| 2017 | Wearable robotic exoskeleton for overground gait training in sub-acute and chronic hemiparetic stroke patients: preliminary results | 10.23736/S1973-9087.17.04591-9 |
| 2017 | Robot-assisted procedures in pediatric neurosurgery | 10.3171/2017.2.FOCUS16579 |
| 2017 | Robot-assisted Salvage Lymph Node Dissection for Clinically Recurrent Prostate Cancer | 10.1016/J.EURURO.2016.08.051 |
| 2017 | Robotic-assisted total mesorectal excision with the single-docking technique for patients with rectal cancer | 10.1186/S12893-017-0315-X |
| 2017 | Rehabilitation in progressive supranuclear palsy: Effectiveness of two multidisciplinary treatments | 10.1371/JOURNAL.PONE.0170927 |
| 2017 | Oncologic outcomes and predictive factors for recurrence following robot-assisted radical cystectomy for urothelial carcinoma: Multicenter study from Korea | 10.3346/JKMS.2017.32.10.1662 |
| 2017 | Gait training of subacute stroke patients using a hybrid assistive limb: a pilot study | 10.3109/17483107.2015.1129455 |
| 2017 | Transoral robotic surgery base of tongue mucosectomy for head and neck cancer of unknown primary | 10.1111/ANS.13741 |
| 2017 | Effects of Robot-Assisted Training for the Unaffected Arm in Patients with Hemiparetic Cerebral Palsy: A Proof-of-Concept Pilot Study | 10.1155/2017/8349242 |
| 2017 | Robot assisted retroperitoneal lymph-node dissection after adjuvant therapy: Different indications | 10.23736/S0393-2249.16.02740-5 |
| 2017 | Lower extremity robotic exoskeleton training: Case studies for complete spinal cord injury walking | 10.3233/NRE-171461 |
| 2017 | Robotic gait assistive technology as means to aggressive mobilization strategy in acute rehabilitation following severe diffuse axonal injury: a case study | 10.3109/17483107.2016.1139633 |
| 2017 | Kinematic and muscle demand similarities between motor-assisted elliptical training and walking: Implications for pediatric gait rehabilitation | 10.1016/J.GAITPOST.2016.10.018 |
| 2017 | Effects of gait training using the Hybrid Assistive Limb® in recovery-phase stroke patients: A 2-month follow-up, randomized, controlled study | 10.3233/NRE-161424 |
| 2017 | A Comparison of Locomotor Therapy Interventions: Partial-Body Weight−Supported Treadmill, Lokomat, and G-EO Training in People With Traumatic Brain Injury | 10.1016/J.PMRJ.2016.12.010 |
| 2017 | Robotic-assisted gait training combined with transcranial direct current stimulation in chronic stroke patients: A pilot double-blind, randomized controlled trial | 10.3233/RNN-170745 |
| 2017 | Effectiveness of Automated Locomotor Training in Patients with Acute Incomplete Spinal Cord Injury: A Randomized, Controlled, Multicenter Trial | 10.1089/NEU.2016.4643 |
| 2017 | Using robot fully assisted functional movements in upper-limb rehabilitation of chronic stroke patients: Preliminary results | 10.23736/S1973-9087.16.04407-5 |
| 2017 | Cost analysis of minimally invasive radical hysterectomy for cervical cancer performed by a single surgeon in an Italian center: an update in gynecologic oncological field | 10.1007/S13304-017-0462-8 |
| 2017 | Salvage robotic prostatectomy following whole gland high-intensity focused ultrasound with a Sonablate 500 device: technical feasibility and safety | 10.1007/S11701-016-0649-X |
| 2017 | Effectiveness of robotic-assisted gait training in stroke rehabilitation: A retrospective matched control study | 10.1016/J.HKPJ.2016.09.001 |
| 2017 | The effect of obesity on clinical and economic outcomes in robotic endometrial cancer surgery | 10.2147/RSRR.S123108 |
| 2017 | Use of an arm weight-bearing combined with upper-limb reaching apparatus to facilitate motor paralysis recovery in an incomplete spinal cord injury patient: A single case report | 10.1589/JPTS.29.176 |
| 2017 | Early stroke rehabilitation of the upper limb assisted with an electromyography-driven neuromuscular electrical stimulation-robotic arm | 10.3389/FNEUR.2017.00447 |
| 2017 | White matter changes in corticospinal tract associated with improvement in arm and hand functions in incomplete cervical spinal cord injury: pilot case series | 10.1038/SCSANDC.2017.28 |
| 2017 | Improved gait speed after robot-assisted gait training in patients with motor incomplete spinal cord injury: A preliminary study | 10.5535/ARM.2017.41.1.34 |
| 2017 | Modified exposure method for gastric mobilization in robotassisted esophagectomy | 10.21037/JTD.2017.11.48 |
| 2018 | Higher robotic colorectal surgery volume improves outcomes | 10.1016/J.AMJSURG.2018.01.042 |
| 2018 | Robotic surgery in public hospitals of Latin-America: a castle of sand? | 10.1007/S00345-018-2227-5 |
| 2018 | Usefulness of robot-assisted thoracoscopic esophagectomy | 10.1007/S11748-018-0897-Y |
| 2018 | A robot-based gait training therapy for pediatric population with cerebral palsy: Goal setting, proposal and preliminary clinical implementation | 10.1186/S12984-018-0412-9 |
| 2018 | Robot Assisted Surgical Ward Rounds: Virtually Always There | 10.14236/JHI.V25I1.982 |
| 2018 | Gynaecological robotic surgery at a state hospital - our own experience | 10.5603/GP.A2018.0084 |
| 2018 | Cost analysis of open radical cystectomy versus robot-assisted radical cystectomy | 10.1111/BJU.14044 |
| 2018 | Motor and psychosocial impact of robotassisted gait training in a real-world rehabilitation setting: A pilot study | 10.1371/JOURNAL.PONE.0191894 |
| 2018 | First 100 consecutive robotic inguinal hernia repairs at a Veterans Affairs hospital | 10.1007/S11701-018-0812-7 |
| 2018 | Single-site robotic cholecystectomy: Comparison of clinical outcome and the learning curves in relation to surgeon experience in a community teaching hospital | 10.1186/S12893-018-0373-8 |
| 2018 | Upper limb robot-assisted therapy in subacute and chronic stroke patients using an innovative end-effector haptic device: A pilot study | 10.3233/NRE-172166 |
| 2018 | Robotic TAPP ventral hernia repair: Early lessons learned at an inner city safety net hospital | 10.4293/JSLS.2017.00070 |
| 2018 | The Hybrid Assistive Limb® intervention for a postoperative patient with spinal dural arteriovenous fistula and chronic spinal cord injury: A case study | 10.1080/10790268.2017.1329916 |
| 2018 | Robotic pancreatoduodenectomy at an experienced institution is not associated with an increased risk of post-pancreatic hemorrhage | 10.1016/J.HPB.2017.11.005 |
| 2018 | Early robot-assisted gait retraining in non-ambulatory patients with stroke: A single blind randomized controlled trial | 10.23736/S1973-9087.18.04832-3 |
| 2018 | Health resource utilization and costs during the first 90 days following robot-assisted hysterectomy | 10.1007/S00192-017-3432-2 |
| 2018 | Outcomes in women undergoing robotic-assisted laparoscopic hysterectomy compared to conventional laparoscopic hysterectomy at a tertiary hospital in Western Australia | 10.1111/AJO.12749 |
| 2018 | Transoral robotic surgery for oncocytic ductal cyst of hypopharynx: a lesion with a high probability of recurrence | 10.1007/S11701-017-0762-5 |
| 2018 | Robotically-driven orthoses exert proximal-to-distal differential recovery on the lower limbs in children with hemiplegia, early after acquired brain injury | 10.1016/J.EJPN.2018.03.002 |
| 2018 | Reliability, validity and discriminant ability of the instrumental indices provided by a novel planar robotic device for upper limb rehabilitation | 10.1186/S12984-018-0385-8 |
| 2018 | Comparative effects of robotic-assisted gait training combined with conventional physical therapy on paretic hip joint stiffness and kinematics between subacute and chronic hemiparetic stroke | 10.3233/NRE-172234 |
| 2018 | Hospital volume and outcomes of robot-assisted partial nephrectomy | 10.1111/BJU.14099 |
| 2018 | Technologically-advanced assessment of upper-limb spasticity: A pilot study | 10.23736/S1973-9087.17.04815-8 |
| 2018 | Robotic-Assisted Versus Thoracoscopic Lobectomy Outcomes From High-Volume Thoracic Surgeons | 10.1016/J.ATHORACSUR.2018.03.048 |
| 2018 | Evolution of upper limb kinematics four years after subacute robot-assisted rehabilitation in stroke patients | 10.1080/00207454.2018.1461626 |
| 2018 | Movement Velocity and Fluidity Improve after Armeo®Spring Rehabilitation in Children Affected by Acquired and Congenital Brain Diseases: An Observational Study | 10.1155/2018/1537170 |
| 2018 | Early experience with ambulatory robotic ventral rectopexy | 10.1016/J.JVISCSURG.2017.05.005 |
| 2018 | An exploration of physiotherapists’ experiences of robotic therapy in upper limb rehabilitation within a stroke rehabilitation centre | 10.1080/17483107.2017.1306593 |
| 2018 | Robot-assisted training using Hybrid Assistive Limb® for cerebral palsy | 10.1016/J.BRAINDEV.2018.04.004 |
| 2018 | Comparison of proximal versus distal upper-limb robotic rehabilitation on motor performance after stroke: A cluster controlled trial | 10.1038/S41598-018-20330-3 |
| 2018 | Reconstruction/Repair of Iatrogenic Biliary Injuries | 10.1097/SLA.0000000000002343 |
| 2018 | Quantification of upper limb motor recovery and EEG power changes after robot-assisted bilateral arm training in chronic stroke patients: A prospective pilot study | 10.1155/2018/8105480 |
| 2018 | Transoral robotic thyroidectomy: lessons learned from an initial consecutive series of 24 patients | 10.1007/S00464-017-5724-5 |
| 2018 | Ankle passive and active movement training in children with acute brain injury using a wearable robot | 10.2340/16501977-2285 |
| 2018 | Robot-assisted gait training effectively improved lateropulsion in subacute stroke patients: A single-blinded randomized controlled trial | 10.23736/S1973-9087.18.05077-3 |
| 2018 | Robot-assisted enucleation of large dumbbell-shaped esophageal schwannoma: A case report | 10.1186/S12893-018-0370-Y |
| 2018 | Short-term Outcomes and Costs Following Partial Nephrectomy in England: A Population-based Study | 10.1016/J.EUF.2017.03.010 |
| 2018 | Robotic Mitral Valve Repair in Older Individuals: An Analysis of The Society of Thoracic Surgeons Database | 10.1016/J.ATHORACSUR.2018.05.074 |
| 2018 | Effects of robot-assisted gait training in chronic stroke patients treated by botulinum toxin-a: A pivotal study | 10.1002/PRI.1718 |
| 2018 | Experience of robotic exoskeleton use at four spinal cord injury model systems centers | 10.1097/NPT.0000000000000235 |
| 2018 | Implementation and evaluation of a sterile compounding robot in a satellite oncology pharmacy | 10.2146/AJHP170461 |
| 2018 | Improving well-being in patients with major neurodegenerative disorders: Differential efficacy of brief social robot-based intervention for 3 neuropsychiatric profiles | 10.2147/CIA.S152561 |
| 2018 | Collaborating with our adult colleagues: A case series of robotic surgery for suspicious and cancerous lesions in children and young adults performed in a free-standing children's hospital | 10.1016/J.JPUROL.2018.01.003 |
| 2018 | Robot-assisted partial nephrectomy for large renal masses: a multi-institutional series | 10.1111/BJU.14139 |
| 2018 | Patency and Incontinence Rates After Robotic Bladder Neck Reconstruction for Vesicourethral Anastomotic Stenosis and Recalcitrant Bladder Neck Contractures: The Trauma and Urologic Reconstructive Network of Surgeons Experience | 10.1016/J.UROLOGY.2018.05.007 |
| 2018 | Robotic-arm assisted total knee arthroplasty is associated with improved early functional recovery and reduced time to hospital discharge compared with conventional jig-based total knee arthroplasty | 10.1302/0301-620X.100B7.BJJ-2017-1449.R1 |
| 2018 | Robotic-assisted versus laparoscopic pancreaticoduodenectomy: oncological outcomes | 10.1007/S00464-017-6002-2 |
| 2018 | Robotic Versus Video-Assisted Thoracoscopic Lung Resection During Early Program Development | 10.1016/j.athoracsur.2017.11.013 |
| 2018 | User-Oriented Evaluation of a Robotic Rollator That Provides Navigation Assistance in Frail Older Adults with and without Cognitive Impairment | 10.1159/000484663 |
| 2018 | Robotic-assisted approach to Median Arcuate Ligament Syndrome with left gastric artery originating directly from the aorta. Report of a case and review of the current mini-invasive treatment modalities | 10.1002/RCS.1919 |
| 2018 | The utilization of fluorescent cholangiography during robotic cholecystectomy at an inner-city academic medical center | 10.1007/S11701-017-0769-Y |
| 2018 | Risk-adapted robotic stereotactic body radiation therapy for inoperable early-stage non-small-cell lung cancer | 10.1007/S00066-017-1194-X |
| 2018 | Robot-assisted laparoscopic partial nephrectomy versus laparoscopic partial nephrectomy: A propensity score-matched comparative analysis of surgical outcomes and preserved renal parenchymal volume | 10.1111/IJU.13529 |
| 2018 | Randomized controlled trial of Amigo® robotically controlled versus manually controlled ablation of the cavo-tricuspid isthmus using a contact force ablation catheter | 10.1007/S10840-018-0319-1 |
| 2018 | Robotic rehabilitation training with a newly developed upper limb single-joint Hybrid Assistive Limb (HAL-SJ) for elbow flexor reconstruction after brachial plexus injury: A report of two cases | 10.1177/2309499018777887 |
| 2018 | Training with Hybrid Assistive Limb for walking function after total knee arthroplasty | 10.1186/S13018-018-0875-1 |
| 2018 | Physiological Responses and Perceived Exertion During Robot-Assisted and Body Weight–Supported Gait After Stroke | 10.1177/1545968318810810 |
| 2018 | Gait training using a hybrid assistive limb (HAL) attenuates head drop: A case report | 10.1016/J.JOCN.2018.03.010 |
| 2018 | Robot-assisted laparoscopic Y-V plasty in 12 patients with refractory bladder neck contracture | 10.1007/S11701-017-0708-Y |
| 2018 | Efficacy and safety outcomes of robotic radical hysterectomy in Chinese older women with cervical cancer compared with laparoscopic radical hysterectomy | 10.1186/S12905-018-0544-X |
| 2018 | A prospective, single-arm study on the use of the da Vinci® Table Motion with the Trumpf TS7000dV operating table | 10.1007/S00464-018-6161-9 |
| 2018 | Initial outcomes from a multicenter study utilizing the indego powered exoskeleton in spinal cord injury | 10.1310/SCI17-00014 |
| 2018 | Use of Hybrid Assistive Limb (HAL®) for a postoperative patient with cerebral palsy: A case report | 10.1186/S13104-018-3311-Z |
| 2018 | The effects of gait training using powered lower limb exoskeleton robot on individuals with complete spinal cord injury | 10.1186/S12984-018-0355-1 |
| 2018 | Overground walking with a robotic exoskeleton elicits trunk muscle activity in people with high-thoracic motor-complete spinal cord injury | 10.1186/S12984-018-0453-0 |
| 2018 | Pilot testing of the spring operated wearable enhancer for arm rehabilitation (SpringWear) | 10.1186/S12984-018-0352-4 |
| 2018 | Using an upper extremity exoskeleton for semi-autonomous exercise during inpatient neurological rehabilitation- a pilot study | 10.1186/S12984-018-0415-6 |
| 2018 | Gait training using a stationary, one-leg gait exercise assist robot for chronic stroke hemiplegia: a case report | 10.1589/JPTS.30.1046 |
| 2018 | Reshaping of gait coordination by robotic intervention in myelopathy patients after surgery | 10.3389/FNINS.2018.00099 |
| 2018 | Effects of high-intensity Robot-assisted hand training on upper limb recovery and muscle activity in individuals with multiple sclerosis: A randomized, controlled, single-blinded trial | 10.3389/FNEUR.2018.00905 |
| 2018 | Electromyography assessment during gait in a robotic exoskeleton for acute stroke | 10.3389/FNEUR.2018.00630 |
| 2018 | Exoskeleton-assisted gait training to improve gait in individuals with spinal cord injury: A pilot randomized study | 10.1186/S40814-018-0247-Y |
| 2018 | Preliminary results of using a voice-controlled robotic camera driver during 3D laparoscopic radical prostatectomy | 10.5173/CEJU.2018.1800 |
| 2018 | Exoskeleton-Robot Assisted Therapy in Stroke Patients: A Lesion Mapping Study | 10.3389/FNINF.2018.00044 |
| 2018 | Outcomes of robotic-assisted radical prostatectomy for patients in two extreme age-groups ( < 50 Years Vs > 65 Years) | 10.15386/CJMED-825 |
| 2018 | Feasibility and Usefulness of a Joystick-Guided Robotic Scope Holder (Soloassist) in Laparoscopic Surgery. | 10.1159/000485524 |
| 2019 | Robot assisted training for the upper limb after stroke (RATULS): a multicentre randomised controlled trial | 10.1016/S0140-6736(19)31055-4 |
| 2019 | Effectiveness of upper-limb robotic-assisted therapy in the early rehabilitation phase after stroke: A single-blind, randomised, controlled trial | 10.1016/J.REHAB.2019.04.002 |
| 2019 | Acute stroke rehabilitation for gait training with cyborg type robot Hybrid Assistive Limb: A pilot study | 10.1016/J.JNS.2019.07.012 |
| 2019 | Effects of a robot intervention on visuospatial hemineglect in postacute stroke patients: a randomized controlled trial | 10.1177/0269215519865993 |
| 2019 | Effectiveness of Robotic Lobectomy-Outcome and Learning Curve in a High Volume Center | 10.1055/S-0038-1639477 |
| 2019 | Robotic correction of iatrogenic ureteral stricture: preliminary experience from a tertiary referral centre | 10.1080/21681805.2019.1651390 |
| 2019 | Robotic-assisted therapy with bilateral practice improves task and motor performance in the upper extremities of chronic stroke patients: A randomised controlled trial | 10.1111/1440-1630.12602 |
| 2019 | Comparative effectiveness of human scope assistant versus robotic scope holder in laparoscopic resection for colorectal cancer | 10.1007/s00464-018-6506-4 |
| 2019 | The learning curve for a surgeon in robot-assisted laparoscopic pancreaticoduodenectomy: a retrospective study in a high-volume pancreatic center | 10.1007/S00464-018-6595-0 |
| 2019 | Robotic-assisted surgery for choledochal cyst in children: early experience at Vietnam National Children’s Hospital | 10.1007/S00383-019-04518-W |
| 2019 | A single-subject study of robotic upper limb training in the subacute phase for four persons with cervical spinal cord injury | 10.1038/S41394-019-0170-3 |
| 2019 | Adjusting assistance commensurates with patient effort during robot-assisted upper limb training for a patient with spasticity after cervical spinal cord injury: A case report | 10.3390/medicina55080404 |
| 2019 | Walking ability following hybrid assistive limb treatment for a patient with chronic myelopathy after surgery for cervical ossification of the posterior longitudinal ligament | 10.1080/10790268.2017.1313932 |
| 2019 | Robotic radical prostatectomy after aborted prostatectomy: still feasible? The experience from a tertiary care center | 10.1007/s11701-018-0870-x |
| 2019 | Robotic-assisted stereotactic real-time navigation: initial clinical experience and feasibility for rectal cancer surgery | 10.1007/S10151-018-1914-Y |
| 2019 | Intensive upper limb therapy including a robotic device after surgically repaired brachial plexus injury: A case study | 10.23736/S1973-9087.19.05415-7 |
| 2019 | Variation in prostate surgery costs and outcomes in the USA: Robot-assisted versus open radical prostatectomy | 10.2217/cer-2018-0109 |
| 2019 | Robotic-assisted versus laparoscopic left pancreatectomy at a high-volume, minimally invasive center | 10.1007/s00464-018-6565-6 |
| 2019 | Salvage robotic-assisted radical prostatectomy: oncologic and functional outcomes from two high-volume institutions | 10.1007/s00345-018-2406-4 |
| 2019 | The effectiveness of a novel cable-driven gait trainer (Robowalk) combined with conventional physiotherapy compared to conventional physiotherapy alone following stroke: a randomised controlled trial | 10.1097/MRR.0000000000000375 |
| 2019 | Comparing Upper Airway Stimulation to Transoral Robotic Base of Tongue Resection for Treatment of Obstructive Sleep Apnea | 10.1002/LARY.27484 |
| 2019 | Safety and effectiveness of the da Vinci robot with the "3+2" mode for distal pancreatectomy | 10.1002/CAM4.2353 |
| 2019 | Randomized Controlled Trial of Gait Training Using Gait Exercise Assist Robot (GEAR) in Stroke Patients with Hemiplegia | 10.1016/J.JSTROKECEREBROVASDIS.2019.06.030 |
| 2019 | A centralized automated-dispensing system in a French teaching hospital: Return on investment and quality improvement | 10.1093/INTQHC/MZY152 |
| 2019 | Oncological and functional outcomes of robotassisted radical cystectomy in bladder cancer patients in a single tertiary center: Can these be preserved throughout the learning curve? | 10.4111/ICU.2019.60.6.463 |
| 2019 | Influence of new technologies on post-stroke rehabilitation: A comparison of Armeo spring to the kinect system | 10.3390/MEDICINA55040098 |
| 2019 | Impact of drug storage systems: A quasi-experimental study with and without an automated-drug dispensing cabinet | 10.1093/INTQHC/MZY155 |
| 2019 | Robot-assisted pyeloplasty for ureteropelvic junction obstruction: Experience from a tertiary referral center | 10.23736/S0393-2249.19.03328-9 |
| 2019 | Unilateral vs Bilateral Hybrid Approaches for Upper Limb Rehabilitation in Chronic Stroke: A Randomized Controlled Trial | 10.1016/J.APMR.2019.06.021 |
| 2019 | Experimental Study on Upper-Limb Rehabilitation Training of Stroke Patients Based on Adaptive Task Level: A Preliminary Study | 10.1155/2019/2742595 |
| 2019 | Robot-assisted gait training for balance and lower extremity function in patients with infratentorial stroke: A single-blinded randomized controlled trial | 10.1186/s12984-019-0553-5 |
| 2019 | Initial Experiences With Robotic Single-Site Thoracic Surgery for Mediastinal Masses | 10.1016/J.ATHORACSUR.2018.08.016 |
| 2019 | Robotic-assisted pedicle screw placement fails to reduce overall postoperative complications in fusion surgery | 10.1016/J.SPINEE.2018.07.004 |
| 2019 | Effect of Stride Management Assist Gait Training for Poststroke Hemiplegia: A Single Center, Open-Label, Randomized Controlled Trial | 10.1016/J.JSTROKECEREBROVASDIS.2018.10.025 |
| 2019 | Recovery and compensation after robotic assisted gait training in chronic stroke survivors | 10.1080/17483107.2018.1466926 |
| 2019 | Comparative effects of different assistance force during robot-assisted gait training on locomotor functions in patients with subacute stroke: An assessor-blind, randomized controlled trial | 10.1097/PHM.0000000000001027 |
| 2019 | Combination of Exoskeletal Upper Limb Robot and Occupational Therapy Improve Activities of Daily Living Function in Acute Stroke Patients | 10.1016/J.JSTROKECEREBROVASDIS.2019.03.006 |
| 2019 | Clinical efficacy of upper limb robotic therapy in people with tetraplegia: a pilot randomized controlled trial | 10.1038/S41393-018-0190-Z |
| 2019 | Preliminary experience with a digital robotic exoscope in cranial and spinal surgery: a review of the Synaptive Modus V system | 10.1007/S00701-019-03953-X |
| 2019 | Effects of robot-(Morning Walk ® ) assisted gait training for patients after stroke: a randomized controlled trial | 10.1177/0269215518806563 |
| 2019 | Comparison of peak oxygen consumption response to aquatic and robotic therapy in individuals with chronic motor incomplete spinal cord injury: a randomized controlled trial | 10.1038/S41393-019-0239-7 |
| 2019 | Secondary Management for Recurrent Ureteropelvic Junction Obstruction after Pyeloplasty: A Comparison of Re-Do Robot-Assisted Laparoscopic Pyeloplasty and Conventional Laparoscopic Pyeloplasty | 10.1159/000503156 |
| 2019 | Safety and immediate effects of Hybrid Assistive Limb in children with cerebral palsy: A pilot study | 10.1016/J.BRAINDEV.2019.10.003 |
| 2019 | Is Robotic Gait Training Feasible in Adults with Disorders of Consciousness? | 10.1097/HTR.0000000000000523 |
| 2019 | Spatiotemporal gait characteristic changes with gait training using the hybrid assistive limb for chronic stroke patients | 10.1016/J.GAITPOST.2019.05.003 |
| 2019 | Robotic Thyroidectomy Decreases Postoperative Pain Compared with Conventional Thyroidectomy | 10.1097/SLE.0000000000000689 |
| 2019 | Effect of EMG-biofeedback robotic-assisted body weight supported treadmill training on walking ability and cardiopulmonary function on people with subacute spinal cord injuries - A randomized controlled trial | 10.1186/S12883-019-1361-Z |
| 2019 | Robot-assisted Partial Nephrectomy: 5-yr Oncological Outcomes at a Single European Tertiary Cancer Center | 10.1016/J.EUF.2017.10.005 |
| 2019 | A follow-up study of the effect of training using the Hybrid Assistive Limb on Gait ability in chronic stroke patients | 10.1080/10749357.2019.1640001 |
| 2019 | The financial burden of setting up a pediatric robotic surgery program | 10.3390/MEDICINA55110739 |
| 2019 | Oncological outcome following TORS in HPV negative supraglottic carcinoma | 10.4103/IJC.IJC_172_18 |
| 2019 | Robot-assisted locomotor training did not improve walking function in patients with chronic incomplete spinal cord injury: A randomized clinical trial | 10.2340/16501977-2547 |
| 2019 | Clinical effects of robot-assisted gait training and treadmill training for Parkinson's disease. A randomized controlled trial | 10.1016/J.REHAB.2019.06.016 |
| 2019 | Robotic-Assisted Shoulder Rehabilitation Therapy Effectively Improved Poststroke Hemiplegic Shoulder Pain: A Randomized Controlled Trial | 10.1016/J.APMR.2019.02.003 |
| 2019 | Robot-assisted surgery for the management of apical prolapse: a bi-centre prospective cohort study | 10.1111/1471-0528.15696 |
| 2019 | Pilot Testing a Robot for Reducing Pain in Hospitalized Preterm Infants | 10.1177/1539449218825436 |
| 2019 | Comparison of perioperative outcomes of robot-assisted partial nephrectomy without renorrhaphy: Comparative outcomes of cT1a versus cT1b renal tumors | 10.1111/IJU.14046 |
| 2019 | Robot for health data acquisition among older adults: A pilot randomised controlled cross-over trial | 10.1136/BMJQS-2018-008977 |
| 2019 | Assistive powered exoskeleton for complete spinal cord injury: Correlations between walking ability and exoskeleton control | 10.23736/S1973-9087.18.05308-X |
| 2019 | Reduced port minimally invasive distal pancreatectomy: single-port laparoscopic versus robotic single-site plus one-port distal pancreatectomy | 10.1007/S00464-018-6361-3 |
| 2019 | Cross-sectional study of patient-reported fatigue, physical activity and cardiovascular status in men after robotic-assisted radical prostatectomy | 10.1007/S00520-019-04794-1 |
| 2019 | Ultrasound-Guided Robotic Enucleation of Pancreatic Neuroendocrine Tumors | 10.1177/1553350618790711 |
| 2019 | Comparison of Open Versus Robotically Assisted Cytoreductive Radical Prostatectomy for Metastatic Prostate Cancer | 10.1016/J.CLGC.2019.05.022 |
| 2019 | In-bore biopsies of the prostate assisted by a remote-controlled manipulator at 1.5 T | 10.1007/S10334-019-00751-5 |
| 2019 | Ergonomics in handheld and robot-assisted camera control: a randomized controlled trial | 10.1007/S00464-019-06678-1 |
| 2019 | Outcomes following TORS for HPV-positive oropharyngeal carcinoma: PEGs, tracheostomies, and beyond | 10.1016/J.AMJOTO.2019.07.003 |
| 2019 | Results of Robotic Thymectomy Performed in Myasthenia Gravis Patients Older Than 60 Years at Onset | 10.1016/J.ATHORACSUR.2019.02.016 |
| 2019 | Minimally Invasive Lobectomy Modality and Other Predictors of Conversion to Thoracotomy | 10.1177/1556984519849037 |
| 2019 | Remote vs. conventional navigation for catheter ablation of atrial fibrillation: insights from prospective registry data | 10.1007/S00392-018-1356-6 |
| 2019 | Sexual, bladder, bowel and ovarian function 1 year after robot-assisted radical hysterectomy for early-stage cervical cancer | 10.1111/AOGS.13680 |
| 2019 | Robotic-assisted laparoscopic surgery for complex hepatolithiasis: a propensity score matching analysis | 10.1007/S00464-018-6547-8 |
| 2019 | Distal versus proximal - An investigation on different supportive strategies by robots for upper limb rehabilitation after stroke: A randomized controlled trial | 10.1186/S12984-019-0537-5 |
| 2019 | Predictors of renal function after open and robot-assisted partial nephrectomy: A propensity score-matched study | 10.1111/IJU.13879 |
| 2019 | Robotic dispensing improves patient safety, inventory management, and staff satisfaction in an outpatient hospital pharmacy | 10.1111/JEP.13014 |
| 2019 | Gait training using the honda walking assistive device® in a patient who underwent total hip arthroplasty: A single-subject study | 10.3390/MEDICINA55030069 |
| 2019 | Oncologic outcomes in patients treated with endoscopic robot assisted simple enucleation (ERASE) for renal cell carcinoma: Results from a tertiary referral center | 10.1016/J.EJSO.2019.03.045 |
| 2019 | Social robots for hospitalized children | 10.1542/PEDS.2018-1511 |
| 2019 | An assessment of early functional rehabilitation and hospital discharge in conventional versus robotic-arm assisted unicompartmental knee arthroplasty | 10.1302/0301-620X.101B1.BJJ-2018-0564.R2 |
| 2019 | Early term effects of robotic assisted gait training on ambulation and functional capacity in patients with spinal cord injury | 10.3906/SAG-1809-7 |
| 2019 | Effect of verticalization with Erigo® in the acute rehabilitation of severe acquired brain injury | 10.1007/S10072-019-03917-0 |
| 2019 | Robotic stereotactic radiotherapy for liver oligometastases from colorectal cancer: a single-center experience | 10.1007/s11547-019-01042-8 |
| 2019 | Long-term survival in patients treated with a robotic radiosurgical device for liver metastases | 10.4143/CRT.2017.594 |
| 2019 | Efficacy and tolerability of cyberknife stereotactic robotic radiotherapy for primary or secondary orbital lesions: A single-center retrospective experience | 10.1177/1533033818818561 |
| 2019 | Robot-assisted trans-gastric drainage and debridement of walled-off pancreatic necrosis using the EndoWrist stapler for the da Vinci Xi: A case report | 10.12998/WJCC.V7.I12.1461 |
| 2019 | Long-term oncologic and functional outcomes after robot-assisted partial nephrectomy in elderly patients | 10.23736/S0393-2249.18.03006-0 |
| 2019 | Cardiopulmonary function after robotic exoskeleton-assisted over-ground walking training of a patient with an incomplete spinal cord injury: Case report | 10.1097/MD.0000000000018286 |
| 2019 | Robotic Assessment of Motor, Sensory, and Cognitive Function in Acute Sport-Related Concussion and Recovery | 10.1089/END.2005.19.382 |
| 2019 | Hybrid assistive limb (HAL) treatment for patients with severe thoracic myelopathy due to ossification of the posterior longitudinal ligament (OPLL) in the postoperative acute/subacute phase: A clinical trial | 10.1080/10790268.2018.1525975 |
| 2019 | Differences in muscle activity and fatigue of the upper limb between Task-Specific training and robot assisted training among individuals post stroke | 10.1016/J.JBIOMECH.2019.04.005 |
| 2019 | Postoperative bladder dysfunction and outcomes after minimally invasive extravesical ureteric reimplantation in children using a laparoscopic and a robot-assisted approach: results of a multicentre international survey | 10.1111/BJU.14785 |
| 2019 | A consecutive 25-week program of gait training, using the alternating hybrid assistive limb (HAL®) robot and conventional training, and its effects on the walking ability of a patient with chronic thoracic spinal cord injury: A single case reversal design | 10.3390/MEDICINA55110746 |
| 2019 | Robot-assisted gallbladder-preserving hepatectomy for treating S5 hepatoblastoma in a child: A case report and review of the literature | 10.12998/WJCC.V7.I7.872 |
| 2019 | Immediate muscle strengthening by an end-effector type gait robot with reduced real-time use of leg muscles: A case series and review of literature | 10.12998/WJCC.V7.I19.2976 |
| 2019 | A paradigm shift: Rehabilitation robotics, cognitive skills training, and function after stroke | 10.3389/FNEUR.2019.01088 |
| 2019 | Comparisons of short-term outcomes between robot-assisted and thoraco-laparoscopic esophagectomy with extended two-field lymph node dissection for resectable thoracic esophageal squamous cell carcinoma | 10.21037/JTD.2019.09.05 |
| 2019 | Kinematic Parameters for Tracking Patient Progress during Upper Limb Robot-Assisted Rehabilitation: An Observational Study on Subacute Stroke Subjects | 10.1155/2019/4251089 |
| 2019 | Long-term follow up data on difficult to treat intracranial arteriovenous malformations treated with the CyberKnife | 10.1016/J.JOCN.2018.10.109 |
| 2019 | Robot-Assisted Laparoscopic Adrenalectomy for Rare Myxoid Adrenocortical Carcinoma | 10.1155/2019/9794345 |
| 2019 | Effectiveness of robot-assisted upper limb training on spasticity, function and muscle activity in chronic stroke patients treated with botulinum toxin: A randomized single-blinded controlled trial | 10.3389/FNEUR.2019.00041 |
| 2019 | A Randomized and Controlled Crossover Study Investigating the Improvement of Walking and Posture Functions in Chronic Stroke Patients Using HAL Exoskeleton – The HALESTRO Study (HAL-Exoskeleton STROke Study) | 10.3389/FNINS.2019.00259 |
| 2019 | Safety and efficacy of robotic elbow training using the upper limb single-joint hybrid assistive limb combined with conventional rehabilitation for bilateral obstetric brachial plexus injury with co-contraction: a case report | 10.1589/JPTS.31.206 |
| 2019 | Robot-assisted stair climbing training on postural control and sensory integration processes in chronic post-stroke patients: A randomized controlled clinical trial | 10.3389/FNINS.2019.01143 |
| 2019 | Work with me, not for me: Relationship between robotic assistance and performance in subacute and chronic stroke patients | 10.1177/2055668319881583 |
| 2019 | Voluntary elbow extension-flexion using single joint hybrid assistive limb (HAL) for patients of spastic cerebral palsy: Two cases report | 10.3389/FNEUR.2019.00002 |
| 2019 | Upper limb motor improvement in chronic stroke after combining botulinum toxin A injection and multi-joints robot-assisted therapy: A case report | 10.1093/OMCR/OMZ097 |
| 2019 | Interaction with social robots: Improving gaze toward face but not necessarily joint attention in children with autism spectrum disorder | 10.3389/FPSYG.2019.01503 |
| 2019 | Dysphagia after Primary Transoral Robotic Surgery with Neck Dissection vs Nonsurgical Therapy in Patients with Low- to Intermediate-Risk Oropharyngeal Cancer | 10.1001/jamaoto.2019.2725 |
| 2019 | Extended thoracic lymph node dissection in robotic-assisted minimal invasive esophagectomy (RAMIE) for patients with superior mediastinal lymph node metastasis. | 10.21037/acs.2019.01.04 |
| 2020 | Factors in the feasibility and safety of outpatient robotic-assisted hysterectomy for endometrial or cervical carcinoma | 10.1016/J.YGYNO.2020.01.028 |
| 2020 | Feasibility of supplemental robot-assisted knee flexion exercise following total knee arthroplasty | 10.3233/BMR-181482 |
| 2020 | Perioperative outcomes and cost of robotic-assisted versus laparoscopic inguinal hernia repair | 10.1007/S00464-019-07128-8 |
| 2020 | Medico-economic impact of robot-assisted lung segmentectomy: What is the cost of the learning curve? | 10.1093/icvts/ivz246 |
| 2020 | The association of robotic lobectomy volume and nodal upstaging in non-small cell lung cancer | 10.1007/S11701-020-01044-Z |
| 2020 | Upper limb robot-assisted rehabilitation versus physical therapy on subacute stroke patients: A follow-up study | 10.1016/J.JBMT.2019.03.016 |
| 2020 | Impact of smart force feedback rehabilitation robot training on upper limb motor function in the subacute stage of stroke | 10.3233/NRE-203130 |
| 2020 | Robotic- versus laparoscopic-assisted distal gastrectomy with D2 lymphadenectomy for advanced gastric cancer based on propensity score matching: short-term outcomes at a high-capacity center | 10.1038/S41598-020-63616-1 |
| 2020 | A review of clinical and oncological outcomes following the introduction of the first robotic colorectal surgery programme to a university teaching hospital in Ireland using a dual console training platform | 10.1007/s11701-020-01073-8 |
| 2020 | Choosing the most appropriate minimally invasive approach to treat gynecologic cancers in the context of an enhanced recovery program: Insights from a comprehensive cancer center | 10.1371/JOURNAL.PONE.0231793 |
| 2020 | Five-day rehabilitation of patients undergoing total knee arthroplasty using an end-effector gait robot as a neuromodulation blending tool for deafferentation, weight offloading and stereotyped movement: Interim analysis | 10.1371/JOURNAL.PONE.0241117 |
| 2020 | Implementation of robotic gynecological surgery in a German University Hospital: patient safety after 110 procedures | 10.1007/S00404-020-05751-8 |
| 2020 | The paradox of the robotic approach to inguinal hernia repair in the inpatient setting | 10.1016/J.AMJSURG.2019.09.012 |
| 2020 | Robotic technology in pediatric neurorehabilitation. A pilot study of human factors in an italian pediatric hospital | 10.3390/IJERPH17103503 |
| 2020 | Robot-assisted Partial Nephrectomy for Complex (PADUA Score ≥10) Tumors: Techniques and Results from a Multicenter Experience at Four High-volume Centers | 10.1016/J.EURURO.2019.03.006 |
| 2020 | Robotic adrenalectomy in the pediatric population: Initial experience case series from a tertiary center | 10.1186/S12894-020-00727-X |
| 2020 | Advances in the rehabilitation of intensive care unit acquired weakness: A case report on the promising use of robotics and virtual reality coupled to physiotherapy | 10.1097/MD.0000000000020939 |
| 2020 | Effects of robot therapy on upper body kinematics and arm function in persons post stroke: a pilot randomized controlled trial | 10.1186/S12984-020-0646-1 |
| 2020 | Neurocognitive robot-assisted rehabilitation of hand function: A randomized control trial on motor recovery in subacute stroke | 10.1186/S12984-020-00746-7 |
| 2020 | Does robot-assisted gait training improve mobility, activities of daily living and quality of life in stroke? A single-blinded, randomized controlled trial | 10.1007/S13760-020-01276-8 |
| 2020 | Robotic therapy for the hemiplegic shoulder pain: A pilot study | 10.1186/S12984-020-00674-6 |
| 2020 | Results and Lessons Learned on Robotic Assisted Kidney Transplantation | 10.1155/2020/8687907 |
| 2020 | Robotic-assisted hand therapy for improvement of hand function in children with cerebral palsy: A case series study | 10.23736/S1973-9087.20.05926-2 |
| 2020 | The ReWalk ReStore™ soft robotic exosuit: A multi-site clinical trial of the safety, reliability, and feasibility of exosuit-augmented post-stroke gait rehabilitation | 10.1186/s12984-020-00702-5 |
| 2020 | Effects of trunk stabilization training robot on postural control and gait in patients with chronic stroke: A randomized controlled trial | 10.1097/MRR.0000000000000399 |
| 2020 | Effects of gait exercise assist robot (GEAR) on subjects with chronic stroke: A randomized controlled pilot trial | 10.1016/J.JSTROKECEREBROVASDIS.2020.104886 |
| 2020 | Robotic-assisted skin sparing mastectomy and immediate reconstruction using latissimus dorsi flap a new effective and safe technique: A comparative study | 10.1016/J.SURONC.2020.09.022 |
| 2020 | Safety and feasibility of exoskeleton-assisted walking during acute/sub-acute SCI in an inpatient rehabilitation facility: A single-group preliminary study | 10.1080/10790268.2019.1671076 |
| 2020 | Impacts of robot implementation on care personnel and clients in elderly-care institutions | 10.1016/J.IJMEDINF.2019.104041 |
| 2020 | Utilization of robotics for retroperitoneal lymph-node dissection in pediatric and non-pediatric hospitals | 10.1007/S11701-020-01066-7 |
| 2020 | Combined robotic surgery for double renal masses and prostate cancer: Myth or reality? | 10.3390/MEDICINA56060318 |
| 2020 | Can kinematic parameters of 3D reach-to-target movements be used as a proxy for clinical outcome measures in chronic stroke rehabilitation? An exploratory study | 10.1186/S12984-020-00730-1 |
| 2020 | A new lower limb portable exoskeleton for gait assistance in neurological patients: A proof of concept study | 10.1186/S12984-020-00690-6 |
| 2020 | Perspectives of child life specialists after many years of working with a humanoid robot in a pediatric hospital: Narrative design | 10.2196/23496 |
| 2020 | Effects of Exoskeletal Lower Limb Robot Training on the Activities of Daily Living in Stroke Patients: Retrospective Pre-Post Comparison Using Propensity Score Matched Analysis | 10.1016/J.JSTROKECEREBROVASDIS.2020.105176 |
| 2020 | Essure removal: comparison of two minimally invasive approaches | 10.1016/j.fertnstert.2020.03.025 |
| 2020 | Learning curve of robot-assisted middle pancreatectomy (RMP): experience of the first 100 cases from a high-volume pancreatic center in China | 10.1007/s00464-019-07133-x |
| 2020 | Kinematic parameters obtained with the ArmeoSpring for upper-limb assessment after stroke: A reliability and learning effect study for guiding parameter use | 10.1186/S12984-020-00759-2 |
| 2020 | Robot-assisted training compared with an enhanced upper limb therapy programme and with usual care for upper limb functional limitation after stroke: The ratuls three-group RCT | 10.3310/HTA24540 |
| 2020 | Robotic iliopubic tract (r-IPT) repair: technique and preliminary outcomes of a minimally invasive tissue repair for inguinal hernia | 10.1007/S10029-020-02259-7 |
| 2020 | A randomized controlled study incorporating an electromechanical gait machine, the Hybrid Assistive Limb, in gait training of patients with severe limitations in walking in the subacute phase after stroke | 10.1371/JOURNAL.PONE.0229707 |
| 2020 | Transoral robotic surgery hypopharyngectomy (TORSH): feasibility and outcomes | 10.1007/S00405-020-05984-Y |
| 2020 | Pure Robotic-assisted Level IV Inferior Vena Cava Thrombectomy for Angiomyolipoma Without Cardiopulmonary Bypass: A First Report | 10.1016/J.UROLOGY.2020.04.068 |
| 2020 | Effects of interactive robot-enhanced hand rehabilitation in treatment of paediatric hand-burns: A randomized, controlled trial with 3-months follow-up | 10.1016/J.BURNS.2020.01.015 |
| 2020 | Combined robotic approach and enhanced recovery after surgery pathway for optimization of costs in patients undergoing proctectomy | 10.1002/BJS5.50281 |
| 2020 | Robot-assisted Gait Training Using Welwalk in Hemiparetic Stroke Patients: An Effectiveness Study with Matched Control | 10.1016/J.JSTROKECEREBROVASDIS.2020.105377 |
| 2020 | Use of a robotic camera holder (FreeHand®) for laparoscopic appendicectomy | 10.1080/13645706.2019.1576052 |
| 2020 | Lessons learned from conducting a pragmatic, randomized, crossover trial on robot-assisted gait training in children with cerebral palsy (PeLoGAIT) | 10.3233/PRM-190614 |
| 2020 | The effect of using Gait Exercise Assist Robot (GEAR) on gait pattern in stroke patients: a cross-sectional pilot study | 10.1080/10749357.2019.1660080 |
| 2020 | Laser ablation of abnormal neurological tissue using robotic neuroblate system (laantern): Procedural safety and hospitalization | 10.1093/NEUROS/NYZ141 |
| 2020 | Relationship between robotic-assisted radical prostatectomy and retropubic radical prostatectomy in the learning curve of a single surgeon as a novice in radical prostatectomy: A retrospective cohort study | 10.1016/J.IJSU.2020.07.006 |
| 2020 | Total oesophago-gastric dissociation in neurologically impaired children: Laparoscopic vs robotic approach | 10.1002/RCS.2048 |
| 2020 | A case report on intensive, robot-assisted rehabilitation program for brainstem radionecrosis | 10.1097/MD.0000000000019517 |
| 2020 | Soft tissue necrosis in patients treated with transoral robotic surgery and postoperative radiotherapy: preliminary results | 10.1177/0300891619900920 |
| 2020 | Robot-assisted gait training is not superior to intensive overground walking in multiple sclerosis with severe disability (the RAGTIME study): A randomized controlled trial | 10.1177/1352458519833901 |
| 2020 | Assessing National Utilization Trends and Outcomes of Robotic and Endoscopic Thyroidectomy in the United States | 10.1177/0194599820927699 |
| 2020 | Surgical outcomes of robotic transanal minimally invasive surgery for selected rectal neoplasms: A single-hospital experience | 10.1016/J.ASJSUR.2019.04.007 |
| 2020 | The Association of Robot-assisted Versus Pure Laparoscopic Radical Nephrectomy with Perioperative Outcomes and Hospital Costs | 10.1016/J.EUF.2018.10.004 |
| 2020 | Robot-assisted orthopedic surgery in the treatment of adult degenerative scoliosis: A preliminary clinical report | 10.1186/S13018-020-01796-2 |
| 2020 | Robotic hepatectomy for benign and malignant liver tumors | 10.1007/S11701-019-00935-0 |
| 2020 | Quality of care perceived by older patients and caregivers in integrated care pathways with interviewing assistance from a social robot: Noninferiority randomized controlled trial | 10.2196/18787 |
| 2020 | First-in-human, robotic-assisted neuroendovascular intervention | 10.1136/NEURINTSURG-2019-015671.REP |
| 2020 | Robotic vs. open surgery in obese women with low-grade endometrial cancer: comparison of costs and quality of life measures | 10.1186/S13584-020-00412-2 |
| 2020 | Transoral robotic surgery for the benefit of patients with head and neck cancer of unknown primary: Our experience at St George's University Hospital, London | 10.1308/RCSANN.2020.0071 |
| 2020 | The long-term oncological outcomes of the 140 robotic sphincter-saving total mesorectal excision for rectal cancer: a single surgeon experience | 10.1007/S11701-019-01037-7 |
| 2020 | Robot-assisted gait training using a very small-sized Hybrid Assistive Limb® for pediatric cerebral palsy: A case report | 10.1016/j.braindev.2019.12.009 |
| 2020 | A prospective analysis of robotic targeted MRI-US fusion prostate biopsy using the centroid targeting approach | 10.1007/S11701-019-00929-Y |
| 2020 | The dose of robot-Assisted gait therapy may influence functional recovery in a multidisciplinary rehabilitation program: An exploratory retrospective study | 10.1097/MRR.0000000000000407 |
| 2020 | An observational study of patho-oncological outcomes of various surgical methods in total mesorectal excision for rectal cancer: A single center analysis | 10.1186/S12893-020-0687-1 |
| 2020 | Gait training using the Honda Walking Assist Device® for individuals with transfemoral amputation: A report of two cases | 10.3233/BMR-191726 |
| 2020 | Comparison of Open and Robot Assisted Radical Nephrectomy With Level I and II Inferior Vena Cava Tumor Thrombus: The Mayo Clinic Experience | 10.1016/J.UROLOGY.2019.11.002 |
| 2020 | Rare and special robotic surgery indications in the pediatric population: ectopic organs and differences of sexual development | 10.1007/S00345-019-02913-5 |
| 2020 | Robotic-assisted computed tomography-guided 18F-FDG PET/computed tomography-directed biopsy for diagnosis of intra thoracic lesions: An experience from a tertiary care centre in North India. | 10.1097/MNM.0000000000001148 |
| 2020 | Impact of previous abdominal surgery on robotic-assisted rectal surgery in patients with locally advanced rectal adenocarcinoma: a propensity score matching study | 10.1186/S12957-020-02086-1 |
| 2020 | Effectiveness of robot-assisted gait training on patients with burns: a preliminary study | 10.1080/10255842.2020.1769080 |
| 2020 | Gait rehabilitation in persons with spinal cord injury using innovative technologies: an observational study | 10.1038/S41393-020-0454-2 |
| 2020 | Para-aortic lymph node surgical staging in locally-advanced cervical cancer: Comparison between robotic versus conventional laparoscopy | 10.1136/IJGC-2019-000961 |
| 2020 | Financial impact of adapting robotics to a thoracic practice in an academic institution | 10.21037/JTD.2019.12.140 |
| 2020 | Robot-assisted partial nephrectomy with a standard laparoscopic ultrasound probe in treating endophytic renal tumor | 10.1016/J.ASJSUR.2019.07.005 |
| 2020 | Early Postoperative Morbidity of Robotic Versus Open Radical Cystectomy in Obese Patients | 10.1089/END.2019.0560 |
| 2020 | Robotic kidney transplantation in the obese patient: 10-year experience from a single center | 10.1111/AJT.15626 |
| 2020 | Assessment of bilateral motor skills and visuospatial attention in children with perinatal stroke using a robotic object hitting task | 10.1186/S12984-020-0654-1 |
| 2020 | The assessment of environmental and external cross-contamination in preparing ready-to-administer cytotoxic drugs: a comparison between a robotic system and conventional manual production | 10.1111/IJPP.12575 |
| 2020 | Survival outcomes for robotic-assisted laparoscopy versus traditional laparoscopy in clinical stage I epithelial ovarian cancer | 10.1016/J.AJOG.2019.10.104 |
| 2020 | Initial report of safety and procedure duration of robotic-assisted chronic total occlusion coronary intervention | 10.1002/CCD.28477 |
| 2020 | Microstructural white matter changes following gait training with Hybrid Assistive Limb initiated within 1 week of stroke onset | 10.1016/J.JNS.2020.116939 |
| 2020 | Factors influencing acceptance of robotics in hospital pharmacy: a longitudinal study using the Extended Technology Acceptance Model | 10.1111/IJPP.12637 |
| 2020 | Cost-effectiveness of four living-donor nephrectomy techniques from a hospital perspective | 10.1093/NDT/GFZ143 |
| 2020 | Time interval between the completion of radiotherapy and robotic-assisted surgery among patients with stage I-III rectal cancer undergoing preoperative chemoradiotherapy | 10.1371/JOURNAL.PONE.0240742 |
| 2020 | Effects of robot-assisted gait training combined with virtual reality on motor and cognitive functions in patients with multiple sclerosis: A pilot, single-blind, randomized controlled trial | 10.3233/RNN-190974 |
| 2020 | Haptic vs sensorimotor training in the treatment of upper limb dysfunction in multiple sclerosis: A multi-center, randomised controlled trial | 10.1016/J.JNS.2020.116743 |
| 2020 | Development and validation of a robotic multifactorial fall-risk predictive model: A one-year prospective study in community-dwelling older adults | 10.1371/JOURNAL.PONE.0234904 |
| 2020 | Impact of initial flexor synergy pattern scores on improving upper extremity function in stroke patients treated with adjunct robotic rehabilitation: A randomized clinical trial | 10.1080/10749357.2020.1738660 |
| 2020 | Evaluation of Insertion Forces and Cochlea Trauma following Robotics-Assisted Cochlear Implant Electrode Array Insertion | 10.1097/MAO.0000000000002608 |
| 2020 | Dynamic Stability and Trunk Control Improvements Following Robotic Balance and Core Stability Training in Chronic Stroke Survivors: A Pilot Study | 10.3389/FNEUR.2020.00494 |
| 2020 | Robotic Exoskeleton Gait Training During Acute Stroke Inpatient Rehabilitation | 10.3389/FNBOT.2020.581815 |
| 2020 | Automated Noncontact Ultrasound Imaging and Ablation System for the Treatment of Atrial Fibrillation: Outcomes of the First-in-Human VALUE Trial | 10.1161/CIRCEP.119.007917 |
| 2020 | Implementation of Stereotactic Accelerated Partial Breast Irradiation Using Cyber-Knife – Technical Considerations and Early Experiences of a Phase II Clinical Study | 10.1007/S12253-020-00821-3 |
| 2020 | Persistent Effect of Gait Exercise Assist Robot Training on Gait Ability and Lower Limb Function of Patients With Subacute Stroke: A Matched Case–Control Study With Three-Dimensional Gait Analysis | 10.3389/FNBOT.2020.00042 |
| 2020 | Effects of robot-assisted gait training in patients with burn injury on lower extremity: A single-blind, randomized controlled trial | 10.3390/JCM9092813 |
| 2020 | Effects of robot-assisted gait training on lower extremity strength, functional independence, and walking function in men with incomplete traumatic spinal cord injury | 10.5606/TFTRD.2020.3316 |
| 2020 | Rehabilitation Improves Mitochondrial Energetics in Progressive Multiple Sclerosis: The Significant Role of Robot-Assisted Gait Training and of the Personalized Intensity. | 10.3390/diagnostics10100834 |
| 2020 | Mobility Skills With Exoskeletal-Assisted Walking in Persons With SCI: Results From a Three Center Randomized Clinical Trial. | 10.3389/frobt.2020.00093 |
| 2020 | Physiotherapists' Experiences Using the Ekso Bionic Exoskeleton with Patients in a Neurological Rehabilitation Hospital: A Qualitative Study | 10.1155/2020/2939573 |
| 2020 | Kinetic Gait Changes after Robotic Exoskeleton Training in Adolescents and Young Adults with Acquired Brain Injury | 10.1155/2020/8845772 |
| 2020 | The Effect of Robot-Assisted Gait Training on Locomotor Function and Functional Capability for Daily Activities in Children with Cerebral Palsy: A Single-Blinded, Randomized Cross-Over Trial. | 10.3390/brainsci10110801 |
| 2020 | Changes in balance, gait and electroencephalography oscillations after robot-assisted gait training: An exploratory study in people with chronic stroke | 10.3390/BRAINSCI10110821 |
| 2020 | Robotic myomectomy for a non-pregnant reproductive age woman with severe acute urinary retention: A case report | 10.1016/J.EUCR.2020.101423 |
| 2020 | Repetitions, duration and intensity of upper limb practice following the implementation of robot assisted therapy with sub-acute stroke survivors: an observational study | 10.1080/17483107.2020.1807621 |
| 2020 | Maximizing efficiency in a high occupancy setting to utilize ultraviolet disinfection for isolation rooms | 10.1016/J.AJIC.2020.05.004 |
| 2020 | Shoulder training using shoulder assistive robot in a patient with shoulder elevation dysfunction: A case report | 10.1016/J.JOS.2019.12.011 |
| 2020 | Differences in Muscle Synergy Symmetry Between Subacute Post-stroke Patients With Bioelectrically-Controlled Exoskeleton Gait Training and Conventional Gait Training | 10.3389/FBIOE.2020.00770 |
| 2020 | Hybrid Assistive Limb improves restricted hip extension after total hip arthroplasty | 10.1080/10400435.2020.1712498 |
| 2020 | Muscular Activity Modulation During Post-operative Walking With Hybrid Assistive Limb (HAL) in a Patient With Thoracic Myelopathy Due to Ossification of Posterior Longitudinal Ligament: A Case Report | 10.3389/FNEUR.2020.00102 |
| 2020 | Robot-assisted laparoscopic partial nephrectomy is a safe and effective option for clinical T2 renal cell carcinoma: a case-series from single-institution | 10.21037/TCR-20-2324 |
| 2020 | Open versus robot-assisted laparoscopic ureteral reimplantation: Hospital charges analysis and outcomes at a single institution | 10.1016/J.JPEDSURG.2019.12.016 |
| 2020 | Robotic-assisted versus open left pancreatectomy for cystic tumours: A single-centre experience | 10.4103/JMAS.JMAS_158_18 |
| 2021 | Locomotor and robotic assistive gait training for children with cerebral palsy | 10.1111/DMCN.14746 |
| 2021 | Outsourcing robotic-assisted operations to private hospitals: cost analysis of a retrospective cohort | 10.1111/ANS.17040 |
| 2021 | Association of surgeon and hospital volume with short-term outcomes after robotassisted radical prostatectomy: Nationwide, population-based study | 10.1371/JOURNAL.PONE.0253081 |
| 2021 | Robotic rehabilitation for end-effector device and botulinum toxin in upper limb rehabilitation in chronic post-stroke patients: an integrated rehabilitative approach | 10.1007/S10072-021-05185-3 |
| 2021 | Managing Patients with Prostate Cancer during COVID-19 Pandemic: The Experience of a High-Volume Robotic Surgery Center | 10.1089/END.2020.0751 |
| 2021 | Effects of wearable ankle robotics for stair and over-ground training on sub-acute stroke: a randomized controlled trial | 10.1186/S12984-021-00814-6 |
| 2021 | An Audit of Robot-Assisted Minimally Invasive Surgeries in Children: Early Experience from a Tertiary Care Center in India | 10.1089/lap.2021.0183 |
| 2021 | Learning curves of minimally invasive donor nephrectomy in a high-volume center: A cohort study of 1895 consecutive living donors | 10.1016/J.IJSU.2020.12.011 |
| 2021 | Energy cost and psychological impact of robotic-assisted gait training in people with spinal cord injury: effect of two different types of devices | 10.1007/S10072-020-04954-W |
| 2021 | Effect of robotic exoskeleton gait training during acute stroke on functional ambulation | 10.3233/NRE-210010 |
| 2021 | Effects of gait training with a voluntary-driven wearable cyborg, Hybrid Assistive Limb (HAL), on quality of life in patients with neuromuscular disease, able to walk independently with aids | 10.1016/J.JOCN.2021.04.038 |
| 2021 | Economic assessment of starting robot-assisted laparoscopic inguinal hernia repair in a single-centre retrospective comparative study: the EASTER study | 10.1093/BJSOPEN/ZRAA046 |
| 2021 | Uptake and outcomes of robotic gynaecological surgery in England (2006–2018): an account of Hospital Episodes Statistics (HES) | 10.1007/S11701-021-01197-5 |
| 2021 | Outcomes of robotic esophagectomies for esophageal cancer by hospital volume: an analysis of the national cancer database | 10.1007/S00464-020-07875-Z |
| 2021 | Learning Curve from 450 Cases of Robot-Assisted Pancreaticoduocectomy in a High-Volume Pancreatic Center Optimization of Operative Procedure and a Retrospective Study | 10.1097/SLA.0000000000003664 |
| 2021 | The fourier M2 robotic machine combined with occupational therapy on post-stroke upper limb function and independence-related quality of life: A randomized clinical trial | 10.1080/10749357.2020.1755815 |
| 2021 | Robotic rectal cancer surgery with single side-docking technique: experience of a tertiary care university hospital | 10.1007/S11701-020-01087-2 |
| 2021 | Robotic Locomotor Training Leads to Cardiovascular Changes in Individuals With Incomplete Spinal Cord Injury Over a 24-Week Rehabilitation Period: A Randomized Controlled Pilot Study | 10.1016/J.APMR.2021.03.018 |
| 2021 | Robotic-assisted unicompartmental knee arthroplasty is associated with earlier discharge from physiotherapy and reduced length-of-stay compared to conventional navigated techniques | 10.1007/s00402-021-04207-1 |
| 2021 | Minimal access rectal cancer surgery: an observational study of patient outcomes from a district general hospital with over a decade of experience with robotic rectal cancer surgery | 10.1111/CODI.15776 |
| 2021 | Case report of pure single-port robotic left lateral sectionectomy using the da Vinci SP system | 10.1097/MD.0000000000028248 |
| 2021 | Robotic-assisted repair of complex ventral hernia: can it pay off? | 10.1007/S11701-020-01078-3 |
| 2021 | Implementation of robot-assisted total mesorectal excision by multiple surgeons in a large teaching hospital: Morbidity, long-term oncological and functional outcome | 10.1002/RCS.2227 |
| 2021 | Beneficial effects of robot-assisted gait training on functional recovery in women after stroke: A cohort study | 10.3390/MEDICINA57111200 |
| 2021 | Effects of robot-assisted rehabilitation on hand function of people with stroke: A randomized, crossover-controlled, assessor-blinded study | 10.5014/AJOT.2021.038232 |
| 2021 | Robot-assisted training after proximal humeral fracture: A randomised controlled multicentre intervention trial | 10.1177/0269215520961654 |
| 2021 | Robotic glove port technique for the endowristed rigid instruments in robotic single-site transabdominal and transvaginal surgery | 10.1007/S11701-020-01093-4 |
| 2021 | Exploring the perceptions of people with dementia about the social robot PARO in a hospital setting | 10.1177/1471301219894141 |
| 2021 | Robot-Assisted Gait Training Plan for Patients in Poststroke Recovery Period: A Single Blind Randomized Controlled Trial | 10.1155/2021/5820304 |
| 2021 | Effect of robotic-assisted gait training on functional status, walking and quality of life in complete spinal cord injury | 10.1097/MRR.0000000000000486 |
| 2021 | Effects of a Brain-Computer Interface-Operated Lower Limb Rehabilitation Robot on Motor Function Recovery in Patients with Stroke | 10.1155/2021/4710044 |
| 2021 | A randomized controlled trial on the effects induced by robot-assisted and usual-care rehabilitation on upper limb muscle synergies in post-stroke subjects | 10.1038/S41598-021-84536-8 |
| 2021 | The use of a UV-C disinfection robot in the routine cleaning process: a field study in an Academic hospital | 10.1186/S13756-021-00945-4 |
| 2021 | Transcranial direct current stimulation combined with robotic training in incomplete spinal cord injury: a randomized, sham-controlled clinical trial | 10.1038/S41394-021-00448-9 |
| 2021 | Effect of robot-assisted gait training on gait automaticity in Parkinson disease: A prospective, open-label, single-arm, pilot study | 10.1097/MD.0000000000024348 |
| 2021 | Exoskeleton-assisted walking improves pulmonary function and walking parameters among individuals with spinal cord injury: a randomized controlled pilot study | 10.1186/S12984-021-00880-W |
| 2021 | Robotic Roux en Y gastric bypass can be safe and cost-effective in a rural setting: clinical outcomes from a community hospital bariatric program | 10.1007/S11701-021-01193-9 |
| 2021 | Robotic and open pancreaticoduodenectomy: results from Taipei Veterans General Hospital in Taiwan | 10.1007/S13304-020-00899-Z |
| 2021 | Effect of Robot Assisted Gait Training on Motor and Walking Function in Patients with Subacute Stroke: A Random Controlled Study | 10.1016/J.JSTROKECEREBROVASDIS.2021.105807 |
| 2021 | Caregiver burden in stroke inpatients: a randomized study comparing robot-assisted gait training and conventional therapy | 10.1007/S13760-020-01465-5 |
| 2021 | Active robotic technologies for total knee arthroplasty | 10.1007/S00402-021-04044-2 |
| 2021 | Robot‐assisted gait training in patients with multiple sclerosis: A randomized controlled crossover trial | 10.3390/MEDICINA57070713 |
| 2021 | The Effect of Applying Robot-Assisted Task-Oriented Training Using Human-Robot Collaborative Interaction Force Control Technology on Upper Limb Function in Stroke Patients: Preliminary Findings | 10.1155/2021/9916492 |
| 2021 | Incisional Lumbodorsal Hernias following Retroperitoneal Robotic Partial Nephrectomies for Small Renal Masses at a High-Volume Tertiary Referral Center | 10.1089/END.2020.0726 |
| 2021 | Is robotic-assisted sacrocolpo(hystero)pexy safe and effective in women over 65 years of age? | 10.1007/S00192-021-04677-2 |
| 2021 | Abnormal synergistic gait mitigation in acute stroke using an innovative ankle–knee–hip interlimb humanoid robot: a preliminary randomized controlled trial | 10.1038/S41598-021-01959-Z |
| 2021 | Outcomes of robotic and laparoscopic cholecystectomy for benign gallbladder disease in Veteran patients | 10.1007/S11701-020-01183-3 |
| 2021 | A Comparison of Colectomy Outcomes Utilizing Open, Laparoscopic, and Robotic Techniques | 10.1177/0003134820973384 |
| 2021 | Evaluation of the enhanced upper limb therapy programme within the Robot-Assisted Training for the Upper Limb after Stroke trial: descriptive analysis of intervention fidelity, goal selection and goal achievement | 10.1177/0269215520953833 |
| 2021 | Myoelectric analysis of upper-extremity muscles during robot-assisted bilateral wrist flexion-extension in subjects with poststroke hemiplegia | 10.1016/J.CLINBIOMECH.2021.105412 |
| 2021 | Patterns of adoption of robotic radical prostatectomy in the United States and England | 10.1111/1475-6773.13706 |
| 2021 | Days alive and out of hospital following transoral robotic surgery: Cohort study of 262 patients with head and neck cancer | 10.1002/HED.26880 |
| 2021 | Clinical validation of kinematic assessments of post-stroke upper limb movements with a multi-joint arm exoskeleton | 10.1186/S12984-021-00875-7 |
| 2021 | Can occupational therapy manpower be replaced with social robots in a singing group during COVID-19? | 10.3233/WOR-205096 |
| 2021 | Outcome after single-site robotic cholecystectomy: An initial single center's experience | 10.1111/ASES.12903 |
| 2021 | The effects of over-ground robot-assisted gait training for children with ataxic cerebral palsy: A case report | 10.3390/S21237875 |
| 2021 | Postoperative quality of life and cosmetic outcome between minimally invasive video-assisted thyroidectomy and bilateral axillo-breast approach robotic thyroidectomy: a single center retrospective cohort study | 10.1007/S13304-021-01035-1 |
| 2021 | Therapeutic effect of AiWalker on balance and walking ability in patients with stroke: A pilot study | 10.1080/10749357.2020.1802969 |
| 2021 | Incidence of acute postoperative robotic port-site hernias: results from a high-volume multispecialty center | 10.1007/S11701-020-01128-W |
| 2021 | Salvage Robotic-assisted Laparoscopic Radical Prostatectomy Following Focal High-Intensity Focused Ultrasound for ISUP 2/3 Cancer | 10.1016/J.UROLOGY.2021.04.059 |
| 2021 | Surgical and oncological outcomes of transoral robotic total laryngectomy: A case series | 10.1016/J.ORALONCOLOGY.2021.105511 |
| 2021 | Robotic-assisted locomotor treadmill therapy does not change gait pattern in children with cerebral palsy | 10.1097/MRR.0000000000000451 |
| 2021 | Comparison of 1-Year Health Care Costs and Use Associated with Open vs Robotic-Assisted Radical Prostatectomy | 10.1001/jamanetworkopen.2021.2265 |
| 2021 | The Cost of Robot-assisted Total Hip Arthroplasty: Comparing Safety and Hospital Charges to Conventional Total Hip Arthroplasty | 10.5435/JAAOS-D-20-00715 |
| 2021 | Clinical utility of an exoskeleton robot using three-dimensional scanner modeling in burn patient: A case report | 10.1093/JBCR/IRAB060 |
| 2021 | Outcomes of robotic coronary artery bypass versus nonrobotic coronary artery bypass | 10.1111/JOCS.15710 |
| 2021 | Comparison of active-assisted and active-unassisted robot-mediated upper limb therapy in subacute stroke | 10.3233/RNN-201010 |
| 2021 | Economic evaluation of robot-assisted training versus an enhanced upper limb therapy programme or usual care for patients with moderate or severe upper limb functional limitation due to stroke: Results from the RATULS randomised controlled trial | 10.1136/BMJOPEN-2020-042081 |
| 2021 | Preliminary clinical experience of robot-assisted surgery in treatment with genioplasty | 10.1038/S41598-021-85889-W |
| 2021 | Ambulatory robot-assisted laparoscopic radical prostatectomy with extended recovery by total extraperitoneal approach | 10.1111/IJU.14509 |
| 2021 | Robotic-Assisted Versus Conventional Posterior Lumbar Fusion—An Analysis of 90-Day Complications and Readmissions | 10.1016/J.WNEU.2021.05.072 |
| 2021 | State-Level Examination of Clinical Outcomes and Costs for Robotic and Laparoscopic Approach to Diaphragmatic Hernia Repair | 10.1016/J.JAMCOLLSURG.2021.05.003 |
| 2021 | Preoperative Bladder Bowel Dysfunction Is the Most Important Predictive Factor for Postoperative Urinary Retention after Robot-Assisted Laparoscopic Ureteral Reimplantation via An Extravesical Approach: A Multi-Center Study | 10.1089/END.2020.0158 |
| 2021 | Comparison of Acute and Chronic Surgical Complications following Robot-Assisted, Laparoscopic, and Traditional Open Radical Prostatectomy among Men in Taiwan | 10.1001/JAMANETWORKOPEN.2021.20156 |
| 2021 | Robot-assisted kidney transplantation: update from the European Robotic Urology Section (ERUS) series | 10.1111/BJU.15199 |
| 2021 | Robotic Inguinal Hernia Repair for Incarcerated Hernias | 10.1089/LAP.2020.0607 |
| 2021 | Patient satisfaction with a pharmacist-led best possible medication discharge plan via tele-robot in a remote and rural community hospital | 10.4103/CJRM.CJRM_74_20 |
| 2021 | Robotic “Double Loop” Roux-en-Y gastric bypass reduces the risk of postoperative internal hernias: a prospective observational study | 10.1007/S00464-020-07901-0 |
| 2021 | Comparison of Sleeve Lobectomy for Lung Cancer Using Mini-Thoracotomy and an Optimized Robot-Assisted Technique | 10.1177/15330338211051547 |
| 2021 | Trends in risk-group distribution and Pentafecta outcomes in patients treated with nerve-sparing, robot-assisted radical prostatectomy: a 10-year low-intermediate volume single-center experience | 10.1007/S00345-020-03206-Y |
| 2021 | Ninety-day morbidity of robot-assisted redo surgery for recurrent rectal prolapse, mesh erosion and pelvic pain: lessons learned from 9 years’ experience in a tertiary referral centre | 10.1111/CODI.15979 |
| 2021 | Early postoperative clinical recovery of robotic arm-assisted vs. image-based navigated Total hip Arthroplasty | 10.1186/S12891-021-04162-3 |
| 2021 | Long-term outcomes of 170 brain arteriovenous malformations treated by frameless image-guided robotic stereotactic radiosurgery: Ramathibodi hospital experience | 10.1097/MD.0000000000025752 |
| 2021 | Gait training with a wearable curara® robot for cerebellar ataxia: a single-arm study | 10.1186/S12938-021-00929-W |
| 2021 | Robotic-Assisted Surgery Results in a Shorter Hospital Stay Following Revisional Bariatric Surgery | 10.1007/s11695-020-05022-0 |
| 2021 | Exoskeleton-Assisted Anthropomorphic Movement Training (EAMT) for Poststroke Upper Limb Rehabilitation: A Pilot Randomized Controlled Trial | 10.1016/J.APMR.2021.06.001 |
| 2021 | Perioperative Outcomes of Robotic Pancreaticoduodenectomy: a Propensity-Matched Analysis to Open and Laparoscopic Pancreaticoduodenectomy | 10.1007/S11605-020-04869-Z |
| 2021 | Pilot experience of simultaneous robotic-assisted partial nephrectomy for bilateral renal tumors-single center analysis | 10.1111/ASES.12831 |
| 2021 | Long-term oncological outcome in patients with cervical cancer after 3 trimodality treatment (radiotherapy, platinum-based chemotherapy, and robotic surgery) | 10.1097/MD.0000000000025271 |
| 2021 | The effects of virtual reality augmented robot-assisted gait training on dual-task performance and functional measures in chronic stroke: A randomized controlled single-blind trial | 10.23736/S1973-9087.21.06441-8 |
| 2021 | Enhancing quality of life in progressive multiple sclerosis with powered robotic exoskeleton | 10.1177/1352458520943080 |
| 2021 | Risk of Upper-body Adverse Events in Robot-assisted Total Laparoscopic Hysterectomy for Benign Gynecologic Disease | 10.1016/J.JMIG.2021.01.017 |
| 2021 | Comparisons between Locomat and Walkbot robotic gait training regarding balance and lower extremity function among non-ambulatory chronic acquired brain injury survivors | 10.1097/MD.0000000000025125 |
| 2021 | Lower local recurrence rate after robot-assisted thoracoscopic esophagectomy than conventional thoracoscopic surgery for esophageal cancer | 10.1038/S41598-021-86420-X |
| 2021 | Overground robot‐assisted gait training for pediatric cerebral palsy | 10.3390/S21062087 |
| 2021 | Effectiveness of robotic exoskeleton-assisted gait training in spinocerebellar ataxia: A case report | 10.3390/S21144874 |
| 2021 | Robotically assisted laparoscopic radical prostatectomy induces lower tissue trauma than radical retropubic prostatectomy | 10.1007/S11701-020-01150-Y |
| 2021 | Reducing loneliness in stationary geriatric care with robots and virtual encounters—a contribution to the covid-19 pandemic | 10.3390/IJERPH18094846 |
| 2021 | Effectiveness and Safety of Moxibustion Robots on Primary Dysmenorrhea: A Randomized Controlled Pilot Trial | 10.1007/S11655-021-3287-8 |
| 2021 | Robotic transcranial magnetic stimulation motor maps and hand function in adolescents | 10.14814/PHY2.14801 |
| 2021 | Effect of gait training using Hybrid Assistive Limb on gait ability and the risk for overwork weakness in the lower limb muscles in patients with neuromuscular disease: A proof-of-concept study | 10.23736/S1973-9087.21.06387-5 |
| 2021 | Using a bimanual lever-driven wheelchair for arm movement practice early after stroke: A pilot, randomized, controlled, single-blind trial | 10.1177/02692155211014362 |
| 2021 | Adjustment effect during shoulder abduction training with the Hybrid Assistive Limb in a patient with postoperative C5 palsy | 10.1016/j.jocn.2021.03.042 |
| 2021 | A cost benefit analysis of increasing surgical technology in lumbar spine fusion | 10.1016/J.SPINEE.2020.10.012 |
| 2021 | Robot-Assisted Arm Training in Stroke Individuals With Unilateral Spatial Neglect: A Pilot Study | 10.3389/FNEUR.2021.691444 |
| 2021 | ATLAS2030 Pediatric Gait Exoskeleton: Changes on Range of Motion, Strength and Spasticity in Children With Cerebral Palsy. A Case Series Study | 10.3389/FPED.2021.753226 |
| 2021 | The effect of pelvic movements of a gait training system for stroke patients: a single blind, randomized, parallel study | 10.1186/S12984-021-00964-7 |
| 2021 | Primary Robot-assisted Retroperitoneal Lymph Node Dissection for Men with Nonseminomatous Germ Cell Tumor: Experience from a Multi-institutional Cohort | 10.1016/J.EUF.2020.06.014 |
| 2021 | Efficacy of a Novel Exoskeletal Robot for Locomotor Rehabilitation in Stroke Patients: A Multi-center, Non-inferiority, Randomized Controlled Trial | 10.3389/FNAGI.2021.706569 |
| 2021 | Effects on the motor function, proprioception, balance, and gait ability of the end-effector robot-assisted gait training for spinal cord injury patients | 10.3390/BRAINSCI11101281 |
| 2021 | Effects of Wearable Powered Exoskeletal Training on Functional Mobility, Physiological Health and Quality of Life in Non-ambulatory Spinal Cord Injury Patients | 10.3346/jkms.2021.36.e80 |
| 2021 | Gait Training after Stroke with a Wearable Robotic Device: A Case Report of Further Improvements in Walking Ability after a Recovery Plateau | 10.2490/PRM.20210037 |
| 2021 | In-Bed Sensorimotor Rehabilitation in Early and Late Subacute Stroke Using a Wearable Elbow Robot: A Pilot Study | 10.3389/FNHUM.2021.669059 |
| 2021 | Robotic Shoulder Rehabilitation With the Hybrid Assistive Limb in a Patient With Delayed Recovery After Postoperative C5 Palsy: A Case Report | 10.3389/FNEUR.2021.676352 |
| 2021 | Robotic Exoskeleton Gait Training in Stroke: An Electromyography-Based Evaluation | 10.3389/FNBOT.2021.733738 |
| 2021 | Immune profiling after minimally invasive lobectomy | 10.1093/ICVTS/IVAA296 |
| 2021 | Evaluation of a novel three-dimensional robotic digital microscope (Aeos) in neurosurgery | 10.3390/CANCERS13174273 |
| 2021 | Comparative assessment of robotic versus classical physical therapy using muscle strength and ranges of motion testing in neurological diseases | 10.3390/JPM11100953 |
| 2021 | Impact of Intensive Gait Training With and Without Electromechanical Assistance in the Chronic Phase After Stroke–A Multi-Arm Randomized Controlled Trial With a 6 and 12 Months Follow Up | 10.3389/FNINS.2021.660726 |
| 2021 | Robotic Pharmacy Implementation and Outcomes in Saudi Arabia: A 21-Month Usability Study. | 10.2196/28381 |
| 2021 | Hybrid assistive limb functional treatment for a patient with chronic incomplete cervical spinal cord injury | 10.2147/IMCRJ.S306558 |
| 2021 | Gait recovery with an overground powered exoskeleton: A randomized controlled trial on subacute stroke subjects | 10.3390/BRAINSCI11010104 |
| 2021 | Robotic Assisted Upper Limb Training Post Stroke: A Randomized Control Trial Using Combinatory Approach Toward Reducing Workforce Demands | 10.3389/FNEUR.2021.622014 |
| 2021 | Hybrid Assistive Limb Intervention for Hemiplegic Shoulder Dysfunction Due to Stroke | 10.7759/CUREUS.19827 |
| 2021 | Effects of Robot-Aided Rehabilitation on the Ankle Joint Properties and Balance Function in Stroke Survivors: A Randomized Controlled Trial | 10.3389/FNEUR.2021.719305 |
| 2021 | Robotic Versus Laparoscopic Colorectal Resection: Are We There Yet? | 10.7759/CUREUS.19698 |
| 2021 | Cardiorespiratory responses to 10 weeks of exoskeleton-assisted overground walking training in chronic nonambulatory patients with spinal cord injury | 10.3390/S21155022 |
| 2021 | A Comparative Efficacy Study of Robotic Priming of Bilateral Approach in Stroke Rehabilitation. | 10.3389/fneur.2021.658567 |
| 2021 | Effect of Training With the Hybrid Assistive Limb on Gait Cycle Kinematics After Total Knee Arthroplasty. | 10.1177/21514593211049075 |
| 2021 | Improved Physiological Gait in Acute and Chronic SCI Patients After Training With Wearable Cyborg Hybrid Assistive Limb | 10.3389/FNBOT.2021.723206 |
| 2021 | Mobile robot-based gait training after total hip arthroplasty (Tha) improves walking in biomechanical gait analysis | 10.3390/JCM10112416 |
| 2021 | Robot-assisted training for upper limb in stroke (ROBOTAS): An observational, multicenter study to identify determinants of efficacy | 10.3390/JCM10225245 |
| 2021 | Functional and ambulatory benefits of robotic-assisted gait training during early subacute inpatient rehabilitation following severe stroke | 10.11622/SMEDJ.2021219 |
| 2021 | Influence of Locomotion Therapy With the Wearable Cyborg HAL on Bladder and Bowel Function in Acute and Chronic SCI Patients | 10.1177/21925682211003851 |
| 2021 | The case report of surgical and medical co-management in a triple organs resection surgery | 10.1016/J.AMSU.2021.102669 |
| 2021 | Electroencephalography as a Biomarker for Functional Recovery in Spinal Cord Injury Patients. | 10.3389/fnhum.2021.548558 |
| 2021 | Effect of using of a lower-extremity exoskeleton on disability of people with multiple sclerosis | 10.1080/17483107.2021.1874064 |
| 2021 | Evaluation of a lower-extremity robotic exoskeleton for people with knee osteoarthritis | 10.1080/10400435.2021.1887400 |
| 2021 | Outcomes of a Multicenter Safety and Efficacy Study of the SuitX Phoenix Powered Exoskeleton for Ambulation by Patients With Spinal Cord Injury | 10.3389/FNEUR.2021.689751 |
| 2021 | Stereotactic body radiation therapy for the treatment of localized prostate cancer in men with underlying inflammatory bowel disease | 10.1186/S13014-021-01850-1 |
| 2021 | Surgeon satisfaction and outcomes of tele-proctoring for robotic gynecologic surgery | 10.1007/S11701-021-01280-X |
| 2021 | Analysis of Gait Motion Changes by Intervention Using Robot Suit Hybrid Assistive Limb (HAL) in Myelopathy Patients After Decompression Surgery for Ossification of Posterior Longitudinal Ligament | 10.3389/FNBOT.2021.650118 |
| 2021 | Impact of Robotic Assisted Surgery on Outcomes in Total Hip Arthroplasty | 10.1016/J.ARTD.2021.04.003 |
| 2021 | Robot-Assisted Electrode Array Insertion Becomes Available in Pediatric Cochlear Implant Recipients: First Report and an Intra-Individual Study | 10.3389/FSURG.2021.695728 |
| 2021 | Single-incision versus multiport robotic myomectomy: A propensity score matched analysis of surgical outcomes and surgical tips | 10.3390/JCM10173957 |
| 2021 | Feasibility of robot-assisted surgery in elderly patients with rectal cancer | 10.4103/JMAS.JMAS_154_19 |
| 2021 | A rare cause of lower gastrointestinal bleeding treated with robotic colorectal surgery | 10.1186/S40792-021-01207-6 |
| 2021 | Situs inversus totalis patients with gastric cancer: Robotic surgery the standard of treatment?-A case report | 10.1016/J.IJSCR.2021.105818 |
| 2021 | Oncological, Surgical and Functional Outcomes of Transoral Robotic Cordectomy for Early Glottic Carcinoma | 10.1016/J.JVOICE.2021.04.024 |
| 2021 | The Route of Motor Recovery in Stroke Patients Driven by Exoskeleton-Robot-Assisted Therapy: A Path-Analysis | 10.3390/MEDSCI9040064 |
| 2021 | Selective Suturing or Sutureless Technique in Robot-assisted Partial Nephrectomy: Results from a Propensity-score Matched Analysis | 10.1016/j.euf.2021.03.019 |
| 2021 | Quality of life after radiation and transoral robotic surgery in advanced oropharyngeal cancer. | 10.1002/lio2.628 |
| 2022 | The pilot study of group robot intervention on pediatric inpatients and their caregivers, using ‘new aibo’ | 10.1007/S00431-021-04285-8 |
| 2022 | The clinical and financial impact of introducing robotic-assisted hysterectomy in a tertiary referral centre: A direct cost analysis of consecutive hysterectomies during a decade | 10.1002/RCS.2343 |
| 2022 | A Case Study of Upper Limb Robotic-Assisted Therapy Using the Track-Hold Device | 10.3390/S22031009 |
| 2022 | Increasing pharmacy productivity and reducing medication turnaround times in an Italian comprehensive cancer center by implementing robotic chemotherapy drugs compounding | 10.1177/1078155221992851 |
| 2022 | Experiences of a “COVID protected” robotic surgical centre for colorectal and urological cancer in the COVID-19 pandemic | 10.1007/S11701-021-01199-3 |
| 2022 | Predictors of complications occurring after open and robot-assisted prostate cancer surgery: a retrospective evaluation of 1062 consecutive patients treated in a tertiary referral high volume center | 10.1007/S11701-021-01192-W |
| 2022 | Outcomes of robotic-assisted vs conventional laparoscopic surgery among patients undergoing resection for rectal cancer: an observational single hospital study of 300 cases | 10.1007/S11701-021-01227-2 |
| 2022 | An observational study of volume–outcome effects for robot-assisted radical prostatectomy in England | 10.1111/bju.15516 |
| 2022 | Robotic-assisted percutaneous coronary intervention in the COVID-19 pandemic | 10.1016/J.JJCC.2021.08.006 |
| 2022 | Risk of chyle leak after robotic versus video-assisted thoracoscopic esophagectomy | 10.1007/S00464-021-08410-4 |
| 2022 | Transitioning a Practice to Robotic Total Knee Arthroplasty Is Correlated with Favorable Short-Term Clinical Outcomes-A Single Surgeon Experience | 10.1055/S-0040-1712984 |
| 2022 | A Robotic Device to Enhance Nursing Home Provider Telepresence During and After the COVID-19 Pandemic | 10.1016/J.JAMDA.2021.11.013 |
| 2022 | Percutaneous microwave ablation versus robot-assisted hepatectomy for early hepatocellular carcinoma: A real-world single-center study | 10.1016/J.DLD.2021.04.008 |
| 2022 | Fertility and Pregnancy Outcomes After Robotic-assisted Laparoscopic Myomectomy in a Canadian Cohort | 10.1016/J.JMIG.2021.06.015 |
| 2022 | Recovery of health-related quality of life in patients undergoing robot-assisted radical cystectomy with intracorporeal diversion | 10.1111/BJU.15505 |
| 2022 | Walking improvement in chronic incomplete spinal cord injury with exoskeleton robotic training (WISE): a randomized controlled trial | 10.1038/S41393-022-00751-8 |
| 2022 | Effects of Balance Exercise Assist Robot training for patients with hemiparetic stroke: a randomized controlled trial | 10.1186/S12984-022-00989-6 |
| 2022 | A randomized clinical control study on the efficacy of three-dimensional upper limb robotic exoskeleton training in chronic stroke | 10.1186/S12984-022-00991-Y |
| 2022 | Comparative effects of EMG-driven robot-assisted therapy versus task-oriented training on motor and daily function in patients with stroke: a randomized cross-over trial | 10.1186/S12984-021-00961-W |
| 2022 | Neurorehabilitation using a voluntary driven exoskeletal robot improves trunk function in patients with chronic spinal cord injury: A single-Arm study | 10.4103/1673-5374.317983 |
| 2022 | Finding the Way to Improve Motor Recovery of Patients with Spinal Cord Lesions: A Case-Control Pilot Study on a Novel Neuromodulation Approach | 10.3390/BRAINSCI12010119 |
| 2022 | Clinical utility of a pediatric hand exoskeleton: identifying users, practicability, and acceptance, and recommendations for design improvement | 10.1186/S12984-022-00994-9 |
| 2022 | MAKO robotic assisted total hip replacement (THR) for patients with fused hips | 10.1002/RCS.2369 |
|  |  |  |
