# Supplementary material for: Robots in Healthcare: a Scoping Review
Source: Curr Robot Rep. 2022 Oct 22;3(4):271–80. doi: 10.1007/s43154-022-00095-4 (PMC9589563; doi:10.1007/s43154-022-00095-4)
Supplement: Supplementary file 3 — Supplementary file3 (DOCX 35 KB) Supplement C. List of all named robots identified, a brief description, the category of robot, and number of publications that explored the robot. Sorted by most to least commonly studied. Excluding robots that were not clearly named in the study. [file 43154_2022_95_MOESM3_ESM.docx]

Supplement C

Title: List of all named robots identified, a brief description, the category of robot, and number of publications that explored the robot. Sorted by most to least commonly studied. Excluding robots that were not clearly named in the study.

| Robot Name | Robot Category | Brief Robot Description | Number of Studies Involving Robot |
| --- | --- | --- | --- |
| da Vinci Surgical System (Intuitive Surgical, USA) | Surgical | Robotic surgery system | 291 |
| Lokomat® (Hocoma, Switzerland) | Rehabilitation and Mobility | Weight assisted treadmill with exoskeleton support | 72 |
| Hybrid Assistive Limb (HAL®) (Cyberdyne, Japan) | Rehabilitation and Mobility | Wearable lower limb exoskeleton | 46 |
| CyberKnife® (Accuray, USA) | Radiotherapy | Robotic radiotherapy device | 18 |
| InMotion® (Bionik, USA) | Rehabilitation and Mobility | Robot assisted upper limb therapy | 18 |
| Armeo®Spring (Hocoma, Switzerland) | Rehabilitation and Mobility | Wearable gait training system | 14 |
| Ekso GT (Ekso Bionics, USA) | Rehabilitation and Mobility | Wearable gait training system | 12 |
| ReoGo (Motorika Medical, Israel) | Rehabilitation and Mobility | Robotic system for upper limb rehabilitation | 12 |
| Bi-Manu-Track (RehaStim, Germany) | Rehabilitation and Mobility | Hand rehabilitation robotic system | 10 |
| MIT-Manus (MIT, USA) | Rehabilitation and Mobility | Upper limb rehabilitation system | 10 |
| Walkbot (Walkbot, Korea) | Rehabilitation and Mobility | Lower limb rehabilitation exoskeleton robot | 7 |
| Amadeo Robotic Sysmte (Tyromotion GmbH, Austria) | Rehabilitation and Mobility | Hand rehabilitation robotic system | 6 |
| Ekso (Ekso Bionics, USA) | Rehabilitation and Mobility | Wearable gait training system | 6 |
| Gait Exercise Assist Robot (GEAR) (Japan) | Rehabilitation and Mobility | Gait training system | 6 |
| PARO (AIST, Japan) | Socially assistive | Therapeutic robotic baby harp seal | 6 |
| Hansen Sensei Robotic Catheter System (Hansen Medical, USA) | Interventional | Remote robotic catheter system | 5 |
| Niobe system (Stereotaxis, USA) | Interventional | Robotically controlled magnetic navigation system for magnetic catheter control | 5 |
| Erigo® (Hocoma, Switzerland) | Rehabilitation and Mobility | Automated tilt table | 5 |
| Gait Trainer (GT) (Rehastim, Germany) | Rehabilitation and Mobility | Gait training system | 5 |
| G-EO (Reha Technology, Switzerland) | Rehabilitation and Mobility | Gait training system | 5 |
| ReWalk (ReWalk Robotics, Israel) | Rehabilitation and Mobility | Wearable powered robotic exoskeleton for hip and knee motion | 5 |
| BD Rowa™ Vmax (BD Rowa, Germany) | Pharmacy | Automated medication dispensing system | 4 |
| Angel Legs (Angel Robotics, Korea) | Rehabilitation and Mobility | Wearable gait training system | 4 |
| Hybrid Assistive Limb Single Joint (HAL-SJ) (Cyberdyne, Japan) | Rehabilitation and Mobility | Wearable single joint training system | 4 |
| Morning Walk® (Curexo, South Korea) | Rehabilitation and Mobility | End-effector gait rehabilitation robot | 4 |
| MOTORE (Humanware, Italy) | Rehabilitation and Mobility | Hand rehabilitation robotic system | 4 |
| Zeus Robotic Surgical System (Computer Motion, USA) | Surgical | Master-slave robotic surgery system with robotic arms | 4 |
| APOTECA Chemo (Loccioni Humancare, Italy) | Telepresence | Automated chemotherapeutic compound preparation | 4 |
| Armeo®Power (Hocoma, Switzerland) | Rehabilitation and Mobility | Robotic exoskeletons for upper extremity rehabilitation | 3 |
| ARMin III (Swiss Federal Institute of Technology Zurich, Switzerland) | Rehabilitation and Mobility | Arm therapy exoskeleton with ergonomic shoulder actuation | 3 |
| Armotion (Reha Technology, Switzerland) | Rehabilitation and Mobility | Mobile upper limb rehabilitation system | 3 |
| Bilateral Exoskeletonal Assistive Robot (Bear-H1) (Milebot Robotics, China) | Rehabilitation and Mobility | Wearable gait training system | 3 |
| Braccio di Ferro (Italy) | Rehabilitation and Mobility | Robotic workstation for arm neurological rehabilitation | 3 |
| Gloreha (Idrogenet, Italy) | Rehabilitation and Mobility | Hand rehabilitation robotic glove | 3 |
| H-Man (Articares, Singapore) | Rehabilitation and Mobility | Portable arm rehabilitation system | 3 |
| Honda Walking Assist (HWA) Device® (Honda Motor Corporation, Japan) | Rehabilitation and Mobility | Wearable gait training system | 3 |
| MAKO (Stryker, USA) | Rehabilitation and Mobility | Joint replacement surgery system | 3 |
| Neuro-Rehabilitation-Robot (NeReBot) (Italy) | Rehabilitation and Mobility | Cable-suspended upper limb rehabilitation system | 3 |
| ROBODOC (Curexo, USA) | Surgical | Robotic arm for joint replacement surgery | 3 |
| Remote Presence (RP) (InTouch Technologies, USA) | Telepresence | Remote telepresence robot controlled via webcam and joystick | 3 |
| Freehand® (Freehand, UK) | Imaging Assistance | Robotic camera control system | 2 |
| Soloassist® (AKTORmed, Germany) | Imaging Assistance | Robotic arm system for laparascope/endoscope control | 2 |
| Amigo™ Remote Catheter System (Catheter Precision, USA) | Interventional | Remote robotic catheter system | 2 |
| CorPath 200 System (Corindus, USA) | Interventional | Robotic percutaneous coronary intervention robot | 2 |
| CorPath GRX System (Corindus, USA) | Interventional | Robotic percutaneous coronary intervention robot | 2 |
| CytoCare™ (Health-Robotics, Italy) | Pharmacy | Chemotherapy compounding robot | 2 |
| Automatic Recovery Arm Motility Integrated System (ARAMIS) (S. Anna Institute, Italy) | Rehabilitation and Mobility | Master-slave exoskeleton robot for paretic upper limb rehabilitation after stroke | 2 |
| CPWalker (Spain) | Rehabilitation and Mobility | Robotic smart walker and exoskeleton for gait rehabilitation | 2 |
| Fourier M2 (Fourier Intelligence, China) | Rehabilitation and Mobility | Height adjustable workstation that provides upper limb rehabilitation | 2 |
| Hyundai Medical Exoskeleton (H-MEX) (Hyundai Motor Company, Korea) | Rehabilitation and Mobility | Wearable exoskeleton gait training system | 2 |
| Hunova (Movendo Technology, Italy) | Rehabilitation and Mobility | Robotic device for rehabilitation and the sensorimotor assessment of the lower limbs and trunk | 2 |
| Indego® (Parker Hannifin Corporation, USA) | Rehabilitation and Mobility | Adjustable lower-limb exoskeleton | 2 |
| Keeogo™ (B-Temia, Canada) | Rehabilitation and Mobility | Lower body, powered knee exoskeleton | 2 |
| KINARM robotic exoskeleton (Kinarm, Canada) | Rehabilitation and Mobility | Robotic device used to assess sensorimotor and cognitive function | 2 |
| Mirror Image Movement Enbaler (MIME) (USA) | Rehabilitation and Mobility | Robotic system for upper limb rehabilitation (incorporating PUMA 560 robot) | 2 |
| SUBAR (Cretem, South Korea) | Rehabilitation and Mobility | Wearable robot with footplate to simulate normal gait | 2 |
| Tibion Bionic Leg (Tibion, USA) | Rehabilitation and Mobility | Battery powered wearable leg orthosis | 2 |
| NAO (SoftBank Robotics, Japan) | Socially assistive | Small autonomous humanoid socially assistive robot | 2 |
| Pepper (SoftBank Robotics, Japan) | Socially assistive | Mobile humanoid socially assistive robot | 2 |
| RoboGait® (Bama Technology, Turkey) | Surgical | Weight assisted treadmill with exoskeletal support | 2 |
| Zeus-TS (Computer Motion, USA) | Surgical | Remote telesurgery application of the Zeus Robotic Surgical System | 2 |
| Apollo-Corabi System (Apollo; Corabi International, USA) | Telepresence | Telepathology and teleconferencing system | 2 |
| Double (Double Robotics, USA) | Telepresence | Self-driving, two-wheeled videoconferencing robot | 2 |
| TUG Automated Delivery System (Aethon, USA) | Delivery and transport | Autonomous mobile delivery robot | 1 |
| Ultra Violet Disinfection Robot® (UVD-Robot) (Clean Room Solutions) | Disinfection | Autonomous ultraviolet disinfection robot | 1 |
| LightStrike™ (Xenex, USA) | Disinfection | Ultraviolet pulse based room disinfection system | 1 |
| Aesculap Aeos® Robotic Digital Microscope (Aesculap, USA) | Imaging Assistance | Robotic arm-mounted 3D imaging and microscopy system with UV and IR fluorescence | 1 |
| AutoLap™ (Medical Surgery Technologies, Israel) | Imaging Assistance | Robotic camera control system | 1 |
| Axilum TMS Robot (Axilum Robotics, France) | Imaging Assistance | Transcranial magnetic stimulation robot | 1 |
| EndoAssist™ (Armstrong Healthcare, UK) | Imaging Assistance | Robotic endoscopic camera control system | 1 |
| Modus V Exoscope (Synaptive Medical, Canada) | Imaging Assistance | Robotic arm-mounted digital microscope | 1 |
| proART™ transducer (BK, USA) | Imaging Assistance | Robotic transducer designed for intraoperative imaging | 1 |
| Carto™ RMT (Biosense Webster, USA) | Interventional | Remote robotic catheter system | 1 |
| Helios® II Catheter (Stereotaxis, USA) | Interventional | Remote magnetic robotic catheter system | 1 |
| Low-intensity collimated ultrasound system (LICU) (VytronUS, USA) | Interventional | Ultrasound guided robotic ablation system | 1 |
| Vdrive (Stereotaxis, USA) | Interventional | Robotically controlled magnetic navigation system for magnetic catheter control | 1 |
| OmniRx (Omnicell, USA) | Pharmacy | Automated medication dispensing system | 1 |
| Parata Max (McKesson, USA) | Pharmacy | High speed vial filling and organising robot | 1 |
| ROWA Box system (BD Rowa, Germany) | Pharmacy | Automated medication dispensing system | 1 |
| ROWA Prolog (BD Rowa, Germany) | Pharmacy | Automated medication dispensing system | 1 |
| ROWA Select (BD Rowa, Germany) | Pharmacy | Automated medication dispensing system | 1 |
| ROWA Speedcase (BD Rowa, Germany) | Pharmacy | Automated medication dispensing system | 1 |
| ScriptPro SP-200 (ScriptPro, USA) | Pharmacy | Automated medication dispensing system | 1 |
| STATION ONCO (Omnicell, USA) | Pharmacy | Automated drug compounding system | 1 |
| AssIstive DEvice for paRalyzed patient (AIDER) (China) | Rehabilitation and Mobility | Powered robotic exoskeleton for walking | 1 |
| AiWalker (China) | Rehabilitation and Mobility | Robotic gait training system | 1 |
| AlterG (Fremont, USA) | Rehabilitation and Mobility | Weight assisted treadmill | 1 |
| Anklebot (MIT, USA) | Rehabilitation and Mobility | Wearable ankle rehabilitation system | 1 |
| ARKE (Bionik Laboratories, Canada) | Rehabilitation and Mobility | Wearable exoskeleton gait training system | 1 |
| Armeo®Boom (Hocoma, Switzerland) | Rehabilitation and Mobility | Overhead sling suspension arm rehabilitation system | 1 |
| Armule® (Intelbot intelligent machine, China) | Rehabilitation and Mobility | Upper limb rehabilitation exoskeleton | 1 |
| ASPIRE (Romania) | Rehabilitation and Mobility | Upper limb rehabilitation system | 1 |
| ATLAS2030 (Marsi Bionics, Spain) | Rehabilitation and Mobility | Wearable gait training system | 1 |
| Autoambulator (HealthSouth, USA) | Rehabilitation and Mobility | Weight assisted treadmill | 1 |
| AVATAR-M (Shanghai Zhanghe Corporation, China) | Rehabilitation and Mobility | Automated lower limb rehabilitation robot | 1 |
| Cable-driven robotic gait training system (CaLT) (USA) | Rehabilitation and Mobility | Weight assisted treadmill | 1 |
| Curara® (Japan) | Rehabilitation and Mobility | Gait rehabilitation robot | 1 |
| Exoskeleton-assisted anthropomorphic movement training (EAMT) (China) | Rehabilitation and Mobility | Robotic exoskeleton for upper limb rehabilitation | 1 |
| ITRI-EXO (Taiwan) | Rehabilitation and Mobility | Lower limb exoskeleton | 1 |
| Exoskeleton ankle robot (Hong Kong) | Rehabilitation and Mobility | Wearable ankle rehabilitation system | 1 |
| FLEXO-Arm1 (Shanghai Electric GeniKIT Medical Science and Technology Co, China) | Rehabilitation and Mobility | Upper limb rehabilitation system | 1 |
| Gait Training and Evaluation System A3 (NX, China) | Rehabilitation and Mobility | Gait training system | 1 |
| Hand of Hope (Rehab-Robotics Co, China) | Rehabilitation and Mobility | Hand rehabilitation robotic system | 1 |
| Haptic Master (FCS Control Systems, Netherlands) | Rehabilitation and Mobility | Haptic robot designed to give users a realistic sense of touch in a virtual or remote world | 1 |
| Healbot T (South Korea) | Rehabilitation and Mobility | Gait training system with weight assisted treadmill and exoskeleton | 1 |
| Hand Exoskeleton Rehabilitation Robot (HEXORR) (USA) | Rehabilitation and Mobility | Wearable hand rehabilitation robotic system | 1 |
| Lever-Actuated Rehabilitation and Ambulation (LARA) (USA) | Rehabilitation and Mobility | Robotic wheelchair for ambulation and upper limb therapy | 1 |
| Light-Exoskeleton (L-EXOS) (Percro, Italy) | Rehabilitation and Mobility | Exoskeleton upper limb rehabilitation system | 1 |
| Linear shoulder robot (IMT, USA) | Rehabilitation and Mobility | Shoulder rehabilitation robot | 1 |
| MAHI Exo-II (USA) | Rehabilitation and Mobility | Upper limb powered exoskeleton | 1 |
| ManBuZhe ([Tian Jin] Rehabilitation Equipment, China) | Rehabilitation and Mobility | Gait training system | 1 |
| Marsi Active Knee (MAK) (Marsi Bionics, Spain) | Rehabilitation and Mobility | Robotic knee orthosis that provides walking assistance | 1 |
| MOBOT (USA) | Rehabilitation and Mobility | Robotic rollator mobility assistant | 1 |
| MRG-P100 Robotic Gait Training System (Hiwin Technologies, Taiwan) | Rehabilitation and Mobility | Weight assisted treadmill | 1 |
| NEUROExos Elbow Module (Italy) | Rehabilitation and Mobility | Powered robotic elbow exoskeleton | 1 |
| Neuro-X system (Apsun, South Korea) | Rehabilitation and Mobility | Upper limb rehabilitation system | 1 |
| Pa10-7 (Mitsubishi, Japan) | Rehabilitation and Mobility | End-effector based industrial robotic arm customised for rehabilitation | 1 |
| ParReEx (Romania) | Rehabilitation and Mobility | Upper limb rehabilitation system | 1 |
| ParReEx Elbow (Romania) | Rehabilitation and Mobility | Upper limb rehabilitation system | 1 |
| Pedi-ICARE (USA) | Rehabilitation and Mobility | Robotic elliptical machine modified for children | 1 |
| Pediatric Hand Exoskeleton (PEXO) (Switzerland) | Rehabilitation and Mobility | Pediatric whole-hand exoskeleton | 1 |
| Phoenix (SuitX, USA) | Rehabilitation and Mobility | Lower limb powered exoskeleton | 1 |
| PKU-RARS (Peking University, China) | Rehabilitation and Mobility | Platform-based ankle–foot device for robotic rehabilitation | 1 |
| Personal transport assistance robot (PTAR) (Toyota, Japan) | Rehabilitation and Mobility | Two wheel transport robot and mobility device | 1 |
| Puma 560 robot manipulator (Unimation, USA) | Rehabilitation and Mobility | End-effector based industrial robotic arm customised for rehabilitation | 1 |
| Reaching Robot (Yasukawa Electric, Japan) | Rehabilitation and Mobility | Arm weight-bearing device combined with upper-limb reaching apparatus to facilitate motor paralysis recovery | 1 |
| REAplan® (Axinesis, Belgium) | Rehabilitation and Mobility | End-effector robot for upper limb rehabilitation | 1 |
| REHABOT (Japan EM, Japan) | Rehabilitation and Mobility | Robotic exoskeletal gait training system | 1 |
| Rehabtek wearable elbow robot (Rehabtek, USA) | Rehabilitation and Mobility | Robotic wearable joint rehabilitation system | 1 |
| Reha-Digit (RehaStim, Germany) | Rehabilitation and Mobility | Upper limb rehabilitation system | 1 |
| ReHapticKnob (Rehabilitation Engineering Laboratory, Switzerland) | Rehabilitation and Mobility | End-effector-based hand rehabilitation robot | 1 |
| Reha-Slide (RehaStim, Germany) | Rehabilitation and Mobility | Upper limb rehabilitation system | 1 |
| Reha-Slide duo (RehaStim, Germany) | Rehabilitation and Mobility | Upper limb rehabilitation system | 1 |
| REX (Rex Bionics, UK) | Rehabilitation and Mobility | Robotic lower limb mobility and rehabilitation device | 1 |
| Robowalk (h/p/cosmos, Germany) | Rehabilitation and Mobility | A robotic cable driven treadmill system for gait rehabilitation | 1 |
| RT600 (Restorative Therapies, USA) | Rehabilitation and Mobility | Stepped ergometer with footplates and surface muscle electrical stimulation | 1 |
| SportsArt ICARE (SportsArt, USA) | Rehabilitation and Mobility | Robotic assistive rehabilitation elliptical trainer | 1 |
| SpringWear (USA) | Rehabilitation and Mobility | Exoskeleton for upper limb rehabilitation | 1 |
| Stride Management Assist (SMA) (Honda, Japan) | Rehabilitation and Mobility | Hip only powered exoskeleton for walking support | 1 |
| Tory platform (Metralabs, Germany) | Rehabilitation and Mobility | Robotic platform for gait training | 1 |
| TrackHold device (Wearable Robotics, Italy) | Rehabilitation and Mobility | Upper limb passive training system with motion tracking support | 1 |
| Trunk Stability Rehabilitation Robot Trainer (3DBT-33) (Man&Tel Co, Korea) | Rehabilitation and Mobility | Rehabilitation robot that can strengthen muscles for trunk stabilization and sit-to-stand training | 1 |
| Wearable ankle robot (Rehabtek, USA) | Rehabilitation and Mobility | Robot ankle rehabilitation device | 1 |
| Wearable Power-Assist Locomotor (WPAL) (Japan) | Rehabilitation and Mobility | Motorised wearable orthosis for walking assistance | 1 |
| WelWalk (Toyota, Japan) | Rehabilitation and Mobility | Robot designed to provide rehabilitation support to patients with lower limb paralysis | 1 |
| Aibo (Sony, Japan) | Socially assistive | Socially assistive robotic dog | 1 |
| Calmer (University of British Columbia, USA) | Socially assistive | Neonatal bed with texture, heartbeat and movement to simulate touch | 1 |
| Huggable (USA) | Socially assistive | Robotic companion bear | 1 |
| MEDi Robot (Rx Robots; Softbank Robotics, USA) | Socially assistive | 2-feet tall human-interactive robot (based on Nao robot) | 1 |
| Zenbo (Asus, Taiwan) | Socially assistive | Mobile socially assistive robot | 1 |
| Zora (Zorabots, Belgium) | Socially assistive | Humanoid socially assistive robots | 1 |
| Robio® Ex (Perfint Healthcare, India) | Surgical | Image guided robotic positioning system | 1 |
| Craniofacial-plastic surgical robot (CPSR-I) (Shanghai Jiao Tong University, China) | Surgical | Craniofacial-plastic surgical robot | 1 |
| da Vinci Table Motion (Intuitive Surgical, USA) | Surgical | Adjustable operating room table for use with da Vinci Surgical System | 1 |
| iotaSOFT (IotaMotion, USA) | Surgical | Cochlear implant robotic surgical system | 1 |
| iSR'obot MonaLisa (Biobiot Surgical, Singapore) | Surgical | Robotic transperineal prostate biopsy system with MRI-ultrasound fusion capability | 1 |
| NAVIO (Smith and Nephew, USA) | Surgical | Hand-held robotic surgical system | 1 |
| NeuroBlate (Monteris Medical, USA) | Surgical | Minimally invasive robotic laser ablation system | 1 |
| PathFinder™ (Prosurgics, UK) | Surgical | Image-guided surgical robot | 1 |
| RIO (Mako, USA) | Surgical | Robotic arm system for surgery | 1 |
| RobOtol (Collin Medical, France) | Surgical | Multi-axis robotic arm for otological surgery | 1 |
| ROSA® Knee System (Zimmer Biomet, USA) | Surgical | Robotic assistant system designed for precise bone resection and alignment during surgery | 1 |
| TIANJI Robot (Tinavi Medical Technology, China) | Surgical | Orthopedic robot with mechanical arm and 3D imaging support | 1 |
| Versius (CMR Surgical, UK) | Surgical | Robotic surgery system | 1 |
| ViKY (Endocontrol, France) | Surgical | Robotic endoscope camera positioning system | 1 |
| Aperio ScanScope CS (Aperio, Canada) | Telepresence | Digital pathology scanner with remote ability | 1 |
| Baylor’s Extra Specialist Seeing You (BESSY) (Baylor Medical Center, USA) | Telepresence | Remote telepresence robot with 2-way audio/video | 1 |
| Comstation (Zydacron, UK) | Telepresence | Remote telepresence robot with 2-way audio/video | 1 |
| Leica TPS2 RM (Lie Microsystems, Canada) | Telepresence | Slide scanner, digital pathology scanner | 1 |
| Temi (Temi, USA) | Telepresence | video-oriented, autonomous personal AI assistant robot | 1 |
| VISITOR1 (Karl Storz, USA) | Telepresence | Internet-based telementoring and telepresence device | 1 |
